# The Effects of a Community-Based Sodium Reduction Program in Rural China – A Cluster-Randomized Trial

**DOI:** 10.1371/journal.pone.0166620

**Published:** 2016-12-09

**Authors:** Nicole Li, Lijing L. Yan, Wenyi Niu, Chen Yao, Xiangxian Feng, Jianxin Zhang, Jingpu Shi, Yuhong Zhang, Ruijuan Zhang, Zhixin Hao, Hongling Chu, Jing Zhang, Xian Li, Jianhong Pan, Zhifang Li, Jixin Sun, Bo Zhou, Yi Zhao, Yan Yu, Michael Engelgau, Darwin Labarthe, Jixiang Ma, Stephen MacMahon, Paul Elliott, Yangfeng Wu, Bruce Neal

**Affiliations:** 1 The George Institute for Global Health at Peking University Health Science Center, Beijing, China; 2 The George Institute for Global Health, Sydney, Australia; 3 Sydney Medical School, the University of Sydney, Sydney, Australia; 4 Department of Preventive Medicine, Feinberg School of Medicine, Northwestern University, Chicago, Illinois, United States of America; 5 School of Public Health, Peking University Health Science Center, Beijing, China; 6 Peking University Clinical Research Institute, Beijing, China; 7 Changzhi Medical College, Changzhi, Shanxi, China; 8 Hebei Province Center for Disease Prevention and Control, Shijiazhuang, Hebei, China; 9 First Hospital of China Medical University, Shenyang, Liaoning, China; 10 Ningxia Medical University, Yinchuan, Ningxia, China; 11 Xi’an Jiaotong University, Xi’an, Shaanxi, China; 12 United States Centers for Disease Control and Prevention, Beijing, China; 13 Chinese Center for Disease Control and Prevention, Beijing, China; 14 Imperial College London, United Kingdom; 15 Royal Prince Alfred Hospital, Sydney; Hunter College, UNITED STATES

## Abstract

**Background:**

Average sodium intake and stroke mortality in northern China are both among the highest in the world. An effective, low-cost strategy to reduce sodium intake in this population is urgently needed.

**Objective:**

We sought to determine the effects of a community-based sodium reduction program on salt consumption in rural northern China.

**Design:**

This study was a cluster-randomized trial done over 18 months in 120 townships (one village from each township) from five provinces. Sixty control villages were compared to 60 intervention villages that were given access to a reduced-sodium, added-potassium salt substitute in conjunction with a community-based health education program focusing on sodium reduction. The primary outcome was the difference in 24-hour urinary sodium excretion between randomized groups.

**Results:**

Among 1,903 people with valid 24-hour urine collections, mean urinary sodium excretion in intervention compared with control villages was reduced by 5.5% (-14mmol/day, 95% confidence interval -26 to -1; p = 0.03), potassium excretion was increased by 16% (+7mmol/day, +4 to +10; p<0.001), and sodium to potassium ratio declined by 15% (-0.9, -1.2 to -0.5; p<0.001). Mean blood pressure differences were -1.1 mm Hg systolic (-3.3 to +1.1; p = 0.33) and -0.7 mm Hg diastolic (-2.2 to +0.8, p = 0.35) and the difference in the proportion with hypertension was -1.3% (-5.1 to 2.5, p = 0.56).

**Conclusion:**

There were clear differences in population sodium and potassium intake between villages that were most likely a consequence of increased use of salt substitute. The absence of effects on blood pressure reflects the moderate changes in sodium and potassium intake achieved.

**Trial Registration:**

Clinicaltrials.gov identifier: NCT01259700.

## Introduction

Stroke is the leading cause of death in China, responsible for about 1.7 million deaths each year [1,2]. Excess sodium intake is a key determinant of high blood pressure [3], the leading cause of stroke [4]. The magnitude of the effect of sodium on blood pressure is such that each 75mmol difference in daily salt intake translates into an approximate 5.4mmHg difference in systolic blood pressure amongst individuals with hypertension and 2.4mmHg amongst individuals with non-hypertensive starting levels of blood pressure[3]. China, especially in the rural areas, has one of the highest sodium intake levels in the world [5,6]. In western populations most dietary sodium derives from processed and restaurant foods but in rural China the majority comes from salt added in cooking and condiments at home [7]. In this situation, the substitution of salt with an alternate product lower in sodium may provide a particular opportunity to deliver a large public health benefit at low cost [8]. Beneficial effects of salt substitution on urinary electrolytes and blood pressure have been achieved in randomised trials done in selected populations including those in rural China [9–14] but whether effects can be achieved in the general community is unknown. We therefore undertook a trial of a pragmatic intervention using a reduced-sodium, added-potassium salt substitute to determine whether this could reduce average population sodium intake in rural China.

## Subjects and Methods

**Fig 1:**
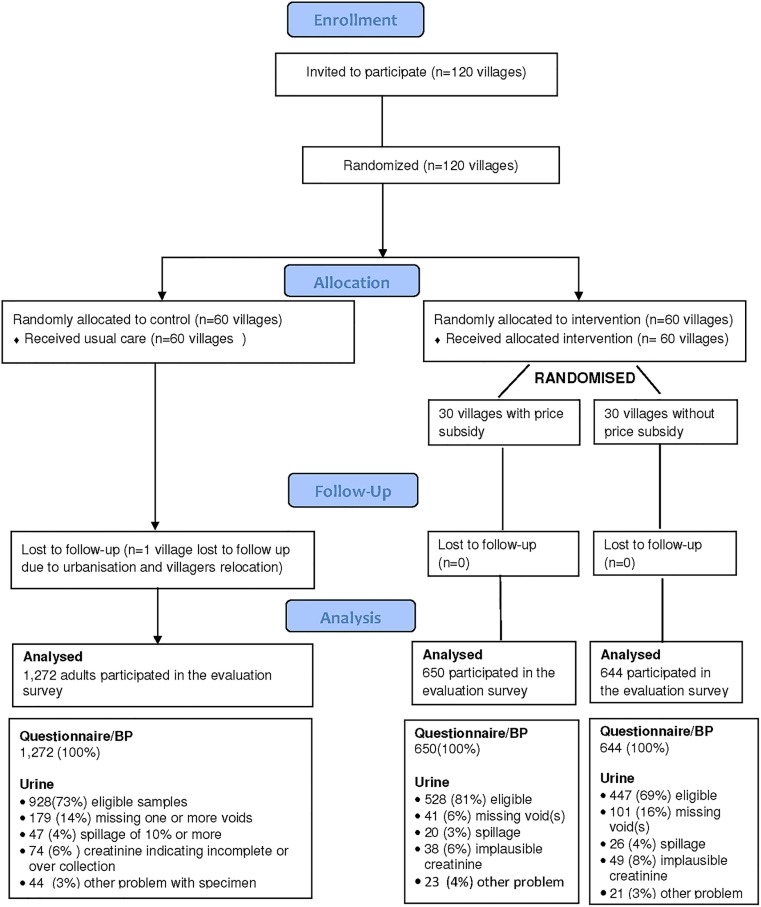
Study CONSORT Flow Diagram.

Details of the rationale and design of the China Rural Health Initiative Sodium Reduction Study have been described previously [15]. In brief, the sodium reduction study is one of the two parallel cluster-randomized controlled trial of the China Rural Health Initiative Project conducted in northern rural China between May 2011 and November 2012. It was done in collaboration with local academic institutions and governments in Hebei, Liaoning, Shanxi and Shaanxi provinces and the Ningxia Autonomous Region. The China Rural Health Initiative was registered with clinicaltrial.gov (NCT01259700). The study design is summarized in Fig 1.

### Ethics

The project was reviewed and approved by the Institutional Review Boards of the Peking University Health Science Center in Beijing, China and of the Duke University Health System in Durham, USA. We have obtained verbal group consent from the chief of each of the study villages to implement the study intervention and obtained written consent from all individuals who participated in the end of the study survey. Verbal group consent from the village chiefs have been approved by the above mentioned ethics committees.

### Provinces, townships and villages included

Two counties were selected from each province/autonomous region and 12 townships from each county, making a total of 120 townships. Within each township one village was selected for participation. The risk of contamination between adjacent intervention and control villages was minimized by the organizational separation of the townships and a geographic separation of 10 kilometers or more between participating villages. A typical township in the study comprised about 17 villages and included 25,000 people with at least one village doctor providing basic health services in each village. The average population size of included villages was 1,867 individuals living in 512 households with an annual per capita income of US$884.

### Randomization

Townships were randomized in a 1:1 ratio to either the sodium reduction program or continued usual practices with stratification by county. Intervention villages were then further assigned at random to receive subsidization of the price of salt substitute, or not, using the same approach to stratification.

### Intervention and control

*Intervention villages*—the salt reduction program comprised community-based health education and availability of reduced-sodium, added-potassium salt substitute at village shops. The health education component was delivered by the township health educators with assistance from the village council and the village doctors through public lectures, the display and distribution of promotional materials, and special interactive education sessions targeted towards individuals at elevated risk of vascular diseases [15]. The salt substitute was made available for purchase in all intervention villages and promoted through the health education component of the intervention. Residents in villages randomized to the price subsidy were provided with coupons that enabled the purchase of salt substitute at the same price as usual salt. *Control villages—*continued their usual practices and were exposed to little by way of efforts to achieve individual or population-wide salt reduction.

### Evaluation

A population survey was done at the end of the intervention period amongst an age- and sex-stratified random sample of 20 or more consenting adults drawn from each of 119 villages, resulting in 2,566 survey participants. One village in the control group was lost to follow-up with the site urbanized and the villagers relocated. The survey included a brief interviewer-administered questionnaire, measurement of blood pressure, height and weight, and the collection of a 24-hour urine sample. Blood pressure was measured in duplicate with the participant seated after 5 minutes rest, using an automated electronic sphygmomanometer (Omron Intellisense HEM 7301 IT) with measurements made at least two minutes apart. Materials and instructions for collecting a timed 24-hour urine sample were provided. On the day of collection, participants were asked to urinate and discard that sample, and then collect all urine in the following 24 hours. At the same time the next day they were asked to urinate and collect that void before completing the 24 hour urine collection. The samples were collected, the volume was recorded, and an aliquot was shipped to a central lab for assay of sodium, potassium, and creatinine.^9^ Individuals that were pregnant, breastfeeding, menstruating, vomiting, had diarrhea, excessive sweating, or symptoms of a urinary tract infection did not provide a urine collection. Urine samples were excluded if participants reported missing the first morning void, missing more than one void, a collection period less than 22 hours or longer than 26 hours, or spilling more than 10% of the total volume. Samples contaminated with faeces were also excluded as were collections that had a volume of less than 500mL or greater than 6000mL, or a 24-hour creatinine excretion of less than 4mmol or greater than 25mmol in women and less than 6mmol or greater than 30mmol in men [16–19].

For each individual, the 24-hour sodium excretion value (mmol/day) was calculated as the concentration of sodium in the urine (mmol/L) multiplied by the urinary volume (L/day). The conversion from sodium (Na) to salt (NaCl) was made by multiplying the sodium value in mmol by 23 to obtain the sodium value in milligrams and then multiplying that figure by 2.54 to obtain the value for salt (NaCl) in milligrams.

### Outcomes

The primary outcome was 24-hour urinary sodium excretion measured as a proxy for salt intake. The secondary outcomes were 24-hour urinary potassium excretion, urinary sodium to potassium ratio, systolic blood pressure, diastolic blood pressure, and the proportion with hypertension (defined as a measured systolic blood pressure of 140mmHg or above, a measured diastolic blood pressure of 90mmHg or above, or the use of blood pressure lowering therapy in the last two weeks). A series of process outcomes measuring knowledge and behaviors relating to salt and salt substitute were also recorded as were spontaneously reported adverse events.

### Statistical power

The study was designed to provide at least 90% power (with a two-sided alpha = 0.05) to detect a difference in mean 24-hour excretion of sodium of at least 11mmol/day (0.65g/day salt) between intervention and control clusters. This estimate assumed a standard deviation of 24-hour sodium excretion of 60mmol/day (observed in study 95mmol/day), an intra-cluster correlation coefficient of 0.05 (observed in study 0.07), and a sample of 2,400 individuals drawn from 120 clusters randomized equally between intervention and control.

### Analysis

Statistical analyses were done using the SAS system version 9.2 (Chinese simplified).

Analysis of treatment effects was by intention to treat with between group comparisons for the study outcomes made using generalized estimating equations (GEE) with clusters accounted for as random effects for continuous outcomes and using GEE logistic regression model with clusters accounted for as fixed effects for categorical outcomes. All statistical analyses were adjusted for the effects of clustering and additional adjustment were not done. Where data were missing, the number of observation was reported and missing values were not imputed. A derived variable was considered missing (for example, 24 hour urinary sodium excretion) when one or more of the related variables was missing (urinary sodium concentration and/or 24 hour urine volume). Generalized estimating equations (GEE) accounting for clustering effects were used to obtain effect estimates, 95% confidence intervals, and p-values for all outcomes. The primary comparison was between intervention clusters (n = 60) and control clusters (n = 59) with outcome data. In addition, the effects of subsidizing the price of the salt substitute were explored by estimating effects in the intervention clusters with price subsidy (n = 30) vs. those without price subsidy (n = 30). Finally, analyses were repeated in participant subgroups defined by median age (58 years), sex, years of education, median BMI (24kg/m^2^), smoking status, and alcohol consumption. A p-value of less than 0.05 was considered significant for the primary comparison but results for secondary analyses and tests of interaction were interpreted in light of the multiple comparisons made.

## Results

The mean age of the 2,566 survey participants was 55 years, 50% were female, and one third smoked (Table 1). Survey participants provided 1,903 eligible urine samples (975 from intervention villages and 928 from control villages) with 663 samples missing entirely or deemed ineligible for another reason. The main reasons for ineligible samples were failure to collect all urine voids (321 individuals) and reported spillage (93 individuals) (Fig 1). There were no differences in the reasons for missing samples between intervention and control villages. Individuals with missing urine samples were noted to have on average lower levels of BMI (23.8 vs. 24.5kg/m^2^; p<0.001) but there were no differences in age and gender.

**Table 1:** Characteristics of survey participants.

	60 intervention villages (n = 1,294)	59 control villages (n = 1,272)
Female (%)	50	50
Age (years) ± SD	55±15	55±14
Formal education (%)*		
≤6 years	54	56
6 to 9 years	38	35
>9 years	8	9
Current smoker (%)	33	30
Drinks alcohol^&^ (%)	25	25
Height (cm) ± SD	161 ±8.5	161±8.9
Weight (kg) ± SD	63 ± 11	63 ±11
Body Mass Index (kg/m^2^) ± SD	24 ±3.5	25 ±3.6

*As reported by participant. SD = standard deviation.

^&^Drinks alcohol is defined as having consumed any type of alcohol in the three months prior to the survey.

Among the 1,903 people with eligible 24-hour urine collections, mean urinary sodium excretion in intervention compared with control villages was 5.5% lower (-14mmol/day, 95% confidence interval -26 to -1; p = 0.03), potassium excretion was 16% higher (+7mmol/day, +4 to +10; p<0.001), and sodium to potassium ratio 15% lower (-0.9, -1.2 to -0.5; p<0.001) (Table 2). The results were similar if analyses were done including urines from 468 additional individuals with urine samples that were not entirely missing but deemed ineligible for the primary analyses (ST1). Mean blood pressure differences were -1.1 mm Hg systolic (-3.3 to +1.1; p = 0.33), -0.7 mm Hg diastolic (-2.2 to +0.8 p = 0.35), and percentage with hypertension was 1.

**Table 2:** Estimated effects of sodium reduction strategy for 60 intervention compared to 59 control villages.

	n	Intervention	Control	Difference between intervention and control (95% confidence interval)	p-value
**Primary outcome**					
Urinary sodium (mmol/day)*	1903	237±97	251±94	-14 (-26 to -1)	0.03
**Secondary outcomes**					
Urinary potassium (mmol/day)*	1903	53±25	45±19	7 (4 to 10)	<0.001
Urinary sodium:potassium ratio*	1903	5.2±3.1	6.1±2.5	-0.9 (-1.2 to -0.5)	<0.001
Systolic blood pressure (mmHg)*	2564	141±22	142±23	-1.1 (-3.3 to 1.*1*)	0.33
Diastolic blood pressure (mmHg)*	2565	86±14	86±14	-0.7 (-2.2 to 0.8)	0.35
Percentage with hypertension^&^ (%)	2566	56	58	-1.3 (-5.1 to 2.5)	0.56
**Process outcomes**					
Know excess salt intake is harmful (%)	2562	84	76	8.3 (5.2 to 11)	<0.001
Know daily limit is ≤6g/day (%)	2564	53	16	37 (33 to 40)	<0.001
Know reducing salt lowers BP (%)	2564	67	52	15 (12 to 19)	<0.001
Concerned about salt in diet (%)	2545	76	62	14 (11 to 18)	<0.001
Household uses salt substitute (%)	2562	62	6	56 (53 to 59)	<0.001
**Other outcomes**					
Antihypertensive drug use (%)	2566	19	21	-2.1 (-5.2 to 1.0)	0.18

*Numbers reported after ± are standard deviations unless otherwise stated

^&^Hypertension defined as having systolic blood pressure at or above 140 mm Hg and/or a diastolic blood pressure at or above 90 mm Hg and/or reports use of blood pressure lowering medication in the last two weeks.

3% lower (-5.1 to 2.5%, p = 0.56) (Table 2). There was no heterogeneity of effects of intervention versus control on sodium excretion in the subgroups studied (all p>0.4).

There was a numerically, but not statistically significant, greater effect of the intervention on the primary outcome with subsidization of the price of salt substitute (p = 0.20) (Table 3). The same was true for all the secondary outcomes (all p>0.06) and the findings were again comparable if analyses included individuals with urine specimens collected but deemed ineligible on the basis of the pre-specified urine inclusion criteria (*ST 2*).

**Table 3:** Estimated effects of sodium reduction strategy for 30 intervention villages *with* price subsidy for salt substitute compared to 30 intervention villages *without* price subsidy for salt substitute.

	With price subsidy		Without price subsidy		Difference between price subsidy and no price subsidy (95% confidence interval)	p-value
**Primary outcome**	n	Mean ± SD	n	Mean ± SD		
Urinary sodium (mmol/day)*	528	232±95	447	243±98	-11 (-29 to 6)	0.20
**Secondary outcomes**	n	Mean ± SD*	n	Mean ± SD*		
Urinary potassium (mmol/day)*	528	55±26	447	51±25	4 (-1 to 9)	0.13
Urinary sodium:potassium ratio*	528	5.0±3.4	447	5.5±2.7	-0.5 (-1.0 to 0.0)	0.06
Systolic blood pressure (mmHg)*	650	140±22	643	141±22	-1.4(-4.6 to 1.8)	0.40
Diastolic blood pressure (mmHg)*	649	86±13	644	86±14	-0.3 (-2.5 to 1.9)	0.80
Percentage with hypertension^&^ (%)	650	54	644	59	-4.4 (-9.7 to 1.0)	0.18
**Process outcomes**	n	%	n	%		
Know excess salt intake is harmful (%)	650	83	643	85	-1.7 (-5.7 to 2.3)	0.52
Know daily limit is ±6g/day (%)	650	56	643	50	5.2 (-0.20 to 11)	0.30
Know reducing salt lowers BP (%)	650	67	643	68	-1.7 (-6.8 to 3.4)	0.71
Concerned about salt in diet (%)	647	78	641	74	4.0 (-0.69 to 8.7)	0.23
Household uses salt substitute (%)	650	78	643	44	35 (30 to 40)	<0.001
**Other outcomes**	n	%	n	%		
Antihypertensive drug use (%)	650	18	644	20	-1.9 (-6.2 to 2.4)	0.40

*Mean ± standard deviations unless otherwise stated.

^&^Hypertension defined as having systolic blood pressure at or above 140 mm Hg and/or a diastolic blood pressure at or above 90 mm Hg and/or reports use of blood pressure lowering medication in the last two weeks.

There was greater knowledge about the adverse effects of salt, the harm caused by high blood pressure, concern about the levels of salt in the diet, and the recommended upper limit for daily salt consumption in intervention compared to control villages (all p<0.001) (Table 2). Use of salt substitute was ten-fold higher in the intervention villages and among these villages, the reported use of salt substitute was double in the villages with the price subsidy compared to the villages without the price subsidy (Table 3).

The most commonly reported adverse events occurring during the study period were dizziness, headache, weakness, stomach ache, hypotension, and fall which occurred at similar rates in the two randomised groups (all p>0.41). Serious vascular events were reported by few of those surveyed with no significant differences between randomised groups for stroke, coronary heart disease, heart failure, kidney disease or hyperkalaemia (all p>0.15).

## Discussion

The salt reduction program achieved the intended lower urinary sodium excretion with a reduction of about three quarters of a gram of salt per day against a background daily consumption of approximately fourteen grams. The increase in urinary potassium, fall in the urinary sodium to potassium ratio, and ten-fold higher reported use of salt substitute among intervention compared to control villages, suggest that the fall in urinary sodium consumption was achieved primarily through use of salt substitute. Effects on blood pressure were modest and non-significant but consistent in magnitude with the fall in sodium reduction achieved. A larger study would be required to reliably detect any effect on blood pressure of the observed changes in sodium consumption.

Salt substitute costs about twice as much as usual salt (4CNY [US$0.65] vs. 2CNY [US$0.33] per kg). While salt substitute is still a low cost condiment, we anticipated that this would be a disincentive to use. Accordingly, we investigated the effect of price subsidy and found the uptake of salt substitute in villages with a subsidy to be almost twice that of villages with no price subsidy. If the price differential could be removed, or salt substitute could in some other way replace usual salt as the staple commodity, then average population sodium consumption could likely be reduced by considerably more than the 5.5% reduction achieved in this trial. A larger sodium reduction, in conjunction with a rise in potassium, would be expected to translate into major public health benefits resulting from reductions in blood pressure levels, the incidence of stroke, and other blood pressure-related diseases [3]. Given that effects of salt substitutes are larger amongst individuals with higher baseline blood pressure levels the magnitude of protection would be expected to be greater amongst hypertensives.

The trial has a number of strengths. It was carried out in a rural setting in China where most sodium in the diet is from cooking or condiment use and where salt substitute has the greatest potential to reduce sodium intake [20]. The trial benefitted from its large size and robust randomized design, and included gold standard assessment of dietary sodium consumption using 24-hour urine collections. The exclusion of individuals with suspected inaccurate or incomplete 24hr urine samples maximized the precision of the estimates in each group and our capacity to detect changes. It was, however, an open rather than a blinded study and to avoid bias considerable efforts were required to ensure that the population surveys were done identically in intervention and control villages [15]. The study was powered to detect effects on urinary markers of sodium and potassium consumption but was not designed to detect effects on blood pressure and hypertension which were specified only as secondary outcomes. While the intended sample size was not achieved the study still had reasonable power to detect the primary effect under investigation. Baseline survey data were not available because of resource constraints. Although an assessment based upon follow-up surveys alone was an entirely valid approach to evaluation of the intervention, the lack of baseline data reduced the statistical power and made the treatment effect comparison between two treatment groups less robust. It is also possible that there were chance baseline imbalances between groups that this design would not have controlled for. Antihypertensive drug use can influence urinary electrolyte excretion but seems unlikely to have substantively influenced trial results given the comparable rates of usage in each randomised group. Salt substitutes have been available in multiple markets around the world including China for many years and are considered safe and well-tolerated.[21–24] Accordingly we did not systematically collect tolerability data during this study and advised use in the intervention communities according to established practices in China.

There are eight randomized trials of salt substitute published to date with six reporting significant falls in systolic blood pressure [9–14] and the remainder trends toward blood pressure reduction[21,22,25,26]. These trials all provided salt substitute to intervention participants at no cost and showed much larger reductions in sodium excretion and blood pressure than were achieved with the strategy used in the present study. Our study demonstrates that a low-intensity community-based sodium reduction strategy can lower average sodium intake but also highlights the rather limited impact that can be achieved by this approach. The full potential of salt substitution in rural China will only be delivered by a policy that makes salt substitute the standard condiment for the entire population. Were this polity to be promoted throughout rural China, it should result in a population wide decline in blood pressure and the prevention of many tens of thousands of strokes and heart attacks each year. [3,20]

## Conclusion

Population sodium intake was reduced by our intervention, primarily through increased use of salt substitute. Larger effects could be achieved in rural China by a wholesale switch from salt to salt substitute, with the potential for major public health benefit in this population which is at very high risk of stroke.

## Supporting Information

S1 Table Supplementary Table 1. Estimated effects of sodium reduction strategy for 60 intervention compared to 59 control villages on urinary outcomes (with all urine samples)
(DOCX)

S2 Table Supplementary Table 2. Estimated effects of sodium reduction strategy for 30 intervention villages with price subsidy for salt substitute compared to 30 intervention villages without price subsidy for salt substitute on urinary outcomes (with all urine samples)
(DOCX)

S1 File Final Follow Up Survey Questionnaire Chinese version (original). (DOC)

S2 File Final Follow Up Survey Questionnaire English version (translated). (DOC)

S3 File China Rural Health Initiative Protocol Chinese version (original). (DOC)

S4 File China Rural Health Initiative Protocol English version (translated). (DOC)

S5 File CONSORT Checklist. (DOC)
