# Supplementary material for: The Effects of a Community-Based Sodium Reduction Program in Rural China – A Cluster-Randomized Trial
Source: PLoS One. 2016 Dec 9;11(12):e0166620. doi: 10.1371/journal.pone.0166620 (PMC5147834; doi:10.1371/journal.pone.0166620)
Supplement: S1 Table — Estimated effects of sodium reduction strategy for 60 intervention compared to 59 control villages on urinary outcomes (with all urine samples) (DOCX) [file pone.0166620.s001.docx]

**S1 Table. Supplementary Table 1. Estimated effects of sodium reduction strategy for 60 intervention compared to 59 control villages on urinary outcomes (with all urine samples)**

|  | n | Intervention | Control | Difference between intervention and control (95% confidence interval) | p-value |
| --- | --- | --- | --- | --- | --- |
| **Primary outcome** |  |  |  |  |  |
| Urinary sodium (mmol/day)* | 2371 | 227±101 | 238±98 | *-11 (-23 to 1)* | 0*·*08 |
| **Secondary outcomes** |  |  |  |  |  |
| Urinary potassium (mmol/day)* | 2371 | 50±26 | 44±20 | *6 (3 to 9)* | 0.0003 |
| Urinary sodium:potassium ratio* | 2371 | 5*·*3±3*·*0 | 6*·*0±2*·*6 | *-0.7(-1.1 to -0.3)* | 0.0002 |

*Numbers reported after ± are standard deviations
