# Supplementary material for: The Effects of a Community-Based Sodium Reduction Program in Rural China – A Cluster-Randomized Trial
Source: PLoS One. 2016 Dec 9;11(12):e0166620. doi: 10.1371/journal.pone.0166620 (PMC5147834; doi:10.1371/journal.pone.0166620)
Supplement: S2 Table — Estimated effects of sodium reduction strategy for 30 intervention villages with price subsidy for salt substitute compared to 30 intervention villages without price subsidy for salt substitute on urinary outcomes (with all urine samples) (DOCX) [file pone.0166620.s002.docx]

**S2 Table. Table 2. Estimated effects of sodium reduction strategy for 30 intervention villages *with* price subsidy for salt substitute compared to 30 intervention villages *without* price subsidy for salt substitute on urinary outcomes (with all urine samples)**

|  | With  price subsidy | | Without  price subsidy | | Difference between price subsidy and no price subsidy (95% confidence interval) | p-value |
| --- | --- | --- | --- | --- | --- | --- |
| **Primary outcome** | n | Mean + SD | n | Mean + SD |  |  |
| Urinary sodium (mmol/day)* | 608 | 223±98 | 599 | 231±103 | -6 (-23 to 11) | 0.51 |
| **Secondary outcomes** | n | Mean + SD* | n | Mean + SD* |  |  |
| Urinary potassium (mmol/day)* | 608 | 52±26 | 599 | 47±25 | 5 (-0.01 to 10) | 0.0505 |
| Urinary sodium:potassium ratio* | 608 | 5.0±3.3 | 599 | 5.6±2.7 | -0.6 (-1.1 to -0.04) | 0.035 |

*Numbers reported after ± are standard deviations
