# Supplementary material for: The Effects of a Community-Based Sodium Reduction Program in Rural China – A Cluster-Randomized Trial
Source: PLoS One. 2016 Dec 9;11(12):e0166620. doi: 10.1371/journal.pone.0166620 (PMC5147834; doi:10.1371/journal.pone.0166620)
Supplement: S1 File — (DOC) [file pone.0166620.s003.doc]

中国农村健康行动调查问卷（2012）

致被访者的话，参考调查培训手册 。询问受访者是否携带身份证，药盒药瓶，并满足测量要求。

开始日期与时间**2012**年**□□**月**□□**日**□□:□□**

（小时 ： 分钟）

| **A. 基本信息** | | | | | | | | |
| --- | --- | --- | --- | --- | --- | --- | --- | --- |
| A.1 姓名： A.2 性别： □_1._男性 □_0._女性  A.3 身份证号**□□□□□□□□□□□□□□□□□□**  A.4 您今年多大年龄？ 岁 A.5 您上过几年学？　年  A.6 您一年在村里住多长时间？ 月 | | | | | | | | |
| **B. 近3个月的生活饮食习惯** | | | | | | | | |
| B.1 最近3个月里，您抽烟吗？ （可多选）  □_0_.不抽烟  □_1._抽香烟 B.1.1您平均每天抽多少支？ 支  □_2_.抽烟叶 B.1.2您平均每周抽多少两？ 两  □_3_.其他 | | | | | | | | |
| B.2 最近3个月里，您喝过酒吗？  □_1_. 喝酒 □_0_. 不喝酒(转B.3)   | B.2.1大约多久喝一次？ B.2.2 一次喝酒折合超过半斤白酒一共有 次?  □_1._<每周1次  □_2._每周1-2次  □_3._每周3-5次  □_4._每天1-2次  □5.>每天2次 | | --- | | | | | | | | | |
| B.3最近3个月里，您平时做体力活（含农活，不含家务）吗？  **（一次 > 30分钟）**  □_0._基本不干 □_1._每周1-2天  □_2._每周3-5天 □_3._几乎每天 | | | B.4最近3个月里，您平时锻炼身体吗？ （一次 > 30分钟）  (如散步、跑步、做操、跳舞、练气功、打太极等） | | | | | |
|  |  |  | □_0._基本不锻炼 □_1._每周1-2天  □_2._每周3-5天 □_3._几乎每天 | | | | | |
| **体检一(PE.1).第一次血压与心率测量** | | | | | | | | |
| PE.1.1 血压水平（收缩压/舒张压）：  PE.1.1.1**□□□**/ PE.1.1.2**□□□** mmHg | | | | | PE1.2 心率：**□□□**次/分钟 | | | |
| B.5 最近3个月，您多长时间吃一次咸菜、榨菜、腌菜之类的食品？  □_1_几乎不吃  □_2_偶尔吃  □_3_每周1-2次  □_4_每周3-6次  □_5_每天至少吃一次 | | | | | B.6 最近3个月，您平时吃饭碰到菜口味淡了的时候，您会怎么办？  □_1_总是加咸菜来吃/加酱油/加盐之类  □_2_有时加咸菜来吃/加酱油/加盐之类  □_3_不介意，照吃 | | | |
| B.7 您认为多吃盐会  □_1_损害健康  □_2_促进健康  □_3_没有影响  □_4_不清楚 | B.8 您认为下面哪些方法能降压 （逐一念）  B.8.1 吃药 □_1._能 □_0._不能 □_9._不知道  B.8.2 吸烟 □_1._能 □_0._不能 □_9._不知道  B.8.3 少吃盐 □_1._能 □_0._不能 □_9._不知道  B.8.4 多吃饭 □_1._能 □_0._不能 □_9._不知道 | | | | | | B.9 您知道成人每天吃多少盐合适？  □_1_ 不吃盐  □_2_ ≤6 克  □_3_ ＞6 克  □_4_ 不清楚 | |
| B.10您日常饮食是否注意少吃盐？  □_1_特别注意(完全不吃咸菜、炒菜完全不用味精且注意少放盐、菜咸了就吃不下)  □_2_比较注意(注意少吃咸菜、炒菜少放盐、碰到菜炒咸了能注意少吃些)  □_3_不注意(对咸菜、家里或餐馆炒菜里的盐无任何减少的要求) | | | | | | | | |
| B.11你们家现在吃低钠盐吗？（*调查员向受访者出示当地包装的低钠盐一袋。对于是否吃低钠盐这一问题，如果受访者不清楚，需要通过电话等方式询问家中的知情人得到答案。*）  □_1_ 吃   | B11.1您家吃低钠盐大概占吃盐总量的多少？%  *（由调查员详细询问家庭用盐情况后估算，具体询问技巧和估算方法参考调查培训手册）*  B11.2 什么时候开始吃低钠盐的？  □_1_ 年 月 □_9_说不清  B11.2.1 从那个时候到现在一直在吃低钠盐吗？  □_1_ 是  □_2_ 不是，累计多长时间没吃低钠盐？ 月（记不清的填999）  □_9_ 不知道 | | --- |   □_2_ 不吃   | B11.3 为什么不吃低钠盐？（可多选）  □_1_附近买不到 □_2_太贵 □_3_不好吃 □_4_吃了没作用 □_5_担心有副作用 □_6_吃了有副作用 (*请注明*)  □_7_对低钠盐不感兴趣 □_8_没听说过低钠盐 □_9_不知道（*受访者本人对家庭用盐情况不掌握*）□_10_其它 (*请注明*) | | --- |   □_3_ 不知道现在吃的是什么盐 | | | | | | | | |
| **C. 现病史** | | | | | | | | |
| C.1 您有冠心病吗？  □_1._有 □_0._没有 □_9._不知道(如没有或不知道，转C.2)   | C.1.1 是县级或以上医院诊断的吗？  □_1._是 □_0._不是 □_9._不知道  C.1.2 医生作诊断的时候，是否告诉您心电图不正常？  □_1._是 □_2._否 □_3._没做过 □_9._不知道，不记得了  C.1.3 最早什么时候诊断的？  _ _ _ _ 年 _ _月 （实在不记得填００００年００月）  C.1.4 最近一次发病是什么时候？  _ _ _ _ 年 _ _月 （实在不记得填００００年００月） | | --- | | | | | C.2 您得过中风吗？  □_1._有 □_0._没有 □_9._不知道(如没有或不知道，转C.3)   | C.2.1 是县级或以上医院诊断的吗？  □_1._是 □_0._不是 □_9._不知道  C.2.2 您得的是脑梗塞还是脑出血？  □_1._脑梗塞 □_2._脑出血 □_9._不知道，不清楚  C.2.3 最早什么时候诊断的？  _ _ _ _年 _ _月 （实在不记得填００００年００月）C.2.4 最近一次发病是什么时候？  _ _ _ _年 _ _月 （实在不记得填００００年００月） | | --- | | | | | |
| C.3 您有糖尿病吗？  □_1._有 □_0._没有 □_9._不知道(如没有或不知道，转C.4)   | C.3.1 是县级或以上医院诊断的吗？  □_1._是 □_0._不是 □_9._不知道  C.3.2 什么时候诊断的？  _ _ _ _年 _ _月 （实在不记得填００００年００月） | | --- | | | | | C.4 您有高血压吗？  □_1._有 □_0._没有 □_9._不知道(如没有或不知道，转C.5)   | C.4.1 患高血压多少年了？ 年 □_9._不知道  C.4.2 是2010年10月前发现的吗？  □_1._是 □_0._不是 □_9._不记得 | | --- | | | | | |
| C.5 您在**50岁（男）/60岁**（女）之后是否测量过血压？  □_1._有 □_0._没有 □_9._不知道，不记得了 (如没有或不知道，不记得，转C.6)   | C.5.1 请问您在**50岁（男）/ 60岁**（女）之后，收缩压（高压）的**最高值**为多少**□□□**mmHg  **（不知道，填999）** | | --- | | | | | | | | | |
| C.6 您**最近两周内**是否在服用降压药？  □_1._是 □_0._不是 □_9._不知道 | | | | | | | | |
| C.7 您在过去的一年内出现过以下症状或病症吗？（逐一读出，有则打叉，没有填“０”，不要留空白）  □_1_头晕 □_2._头疼 □_3._乏力 □_4._跌倒 □_5._牙龈出血或皮下出血 □_6_ 胃疼 □_7_高血钾症 □_8._医生确认的胃出血  □_9_医生确认的肾功能不全 □_10_医生确认的心衰 □_11._医生确认的脑出血  □_12_医生确认的低血压（最低血压值____/____mmHg） □_13_ 医生确认的低血糖（最低血糖值_______mmol/L）  □_14_出现危及生命、 需要住院或延长住院时间的情况（请填写具体情况________________）  □_15_医生确认的其它疾病（请填写具体病名______________________） | | | | | | | | |
| C.8 现在问您一些关于您去找村里村大夫/村医看病的问题。在过去**12个月**里，您是否**每月**去看一次村医？  □_1_. 是  □_0_. 否 | | | | C.9 给您看病时，村医**每次**都量血压吗？  □_1_. 不去看村医  □_2_. 从没量过  □_3_. 有时(偶尔)量  □_4_. 几乎每次量  □_5_. 每次量 | | | | |
| **体检二(PE.2).第二次血压与心率测量** | | | | | | | | |
| PE.2.1 血压水平（收缩压/舒张压）：  PE.2.1.1**□□□**/PE.2.1.2**□□□** mmHg | | | | | PE.2.2 心率：**□□□**次/分钟 | | | |
| |  | | | --- | --- | | **CL．心血管高危个体确认表（由调查员根据前面数据判断填写）** | | | CL.1确诊有冠心病（C.1.1与C.1.2 同时回答是） | □_1._是 □_0._否 | | CL.2确诊得过中风（C.2.1 回答是） | □_1._是 □_0._否 | | CL.3确诊有糖尿病（C.3.1 回答是） | □_1._是 □_0._否 | | CL.4自报50岁（男）/60岁（女）之后曾经收缩压≧160mmHg （C.5.1） | □_1._是 □_0._否 | | CL.5两次测量收缩压读数都大于或等于160mmHg （PE.1和PE.2） | □_1._是 □_0._否 | | **如以上任意一项选择是，请继续调查；**  **否则转到最后PE.3进行身高体重测量。** | | | | | | | | | | |
| **D. 高危个体的用药与诊疗情况** | | | | | | | | |
| D.1 村医**最近一次**给您看心脏病、中风、糖尿病或高血压是多少天前？ 天前 （没找村医看过这类病，填0）  最近**这次**看病时，村医给过您以下建议吗？  D.1.1 少吃盐 □_1_.给过 □_0._没给过  D.1.2 戒烟 □_1_.给过 □_0._没给过 □_9._不适用  D.1.3 不要大量饮酒 □_1_.给过 □_0._没给过 □_9._不适用  D.1.4 适量运动 □_1_.给过 □_0._没给过  D.1.5 控制体重 □_1_.给过 □_0._没给过 □_9._不适用 | | | | | | | | |
| D.2 在过去**12个月**里，您有没有因为心脏病，中风，糖尿病或高血压住过院（因其他原因住院，算没住过）？  □_1._有 □_0._没有 (如没有转D.3)   | D.2.1 一共住了几次医院？ 次  D.2.2 一共住了多少天？ 天  D.2.3 住院**自己**一共花了多少钱？ 元  （如果只知道总额，在总额前加“00”；不知道，填“-999”） | | --- | | | | | | | | | |
| D.3 您过去**12个月里**有多少个月吃**降压药**?  □_0_ 不吃降压药  □_1._不知道吃的是否降压药  □_2._＜2个月  □_3._2至8个月  □_4._9至11个月  □_5._=12个月  （如不吃或不知道吃的是否降压药，转D.4） | | D.3.1 您吃的降压药**主要**是谁开的？  □_1_. 自购  □_2_. 中医  □_3_. 专家（镇或以上）  □_4_. 专家与村医  □_5_. 村医 | | | | | | D.3.3 降压药**主要**在哪里买的？  □_1_. 村诊所  □_2_. 乡、镇卫生服务中心  □_3_. 县医院  □_4_. 药店 |
|  |  | D.3.2.村医有没有给您调过药（包括开新药和剂量的增减）  □_1._有 □_0._没有 □_9._不知道 | | | | | | |
| D.4 您过去**12个月里**有多少个月吃**阿司匹林**?  □_0_ 不吃阿司匹林  □_1._不知道吃的是否阿司匹林  □_2._＜2个月  □_3._2至8个月  □_4._9至11个月  □_5._=12个月  （如不吃或不知道吃的是否阿司匹林，转D.5） | | D.4.1 谁开的阿司匹林？  □_1_. 自购  □_2_. 中医  □_3_. 专家（镇或以上）  □_4_. 专家与村医  □_5_. 村医 | | | | | | D.4.2 阿司匹林**主要**在哪里买的？  □_1_. 村诊所  □_2_. 乡、镇卫生服务中心  □_3_. 县医院  □_4_. 药店 |
| D.5 您**最近一个月内**是否在服用治疗心脏病、中风、或高血压的药物？   | □_1._是 □_9._不知道 | □_0._否 (如否，进行PE.3 身高体重测量) | | --- | --- |  | D.5.1 药物 (商品名) | D.5.2 是否天天吃药？  （过去30天中≥25天服药） | D.5.3 是否村医开的？ | | --- | --- | --- | | 1. | □_1._是 □_0._否 | □_1._是 □_0._否 | | 2. | □_1._是 □_0._否 | □_1._是 □_0._否 | | 3. | □_1._是 □_0._否 | □_1._是 □_0._否 | | 4. | □_1._是 □_0._否 | □_1._是 □_0._否 | | 5. | □_1._是 □_0._否 | □_1._是 □_0._否 | | 如果超过5个，且自己和受访者都不能确定药物种类，就把超过5个的药物都抄在下面空白处，并标出D5.2 和D5.3 的数字答案（1为是或0为否）。 | | | | | | | | | | | |
| **体检三(PE.3).（所有受访者，含非高危个体，都进行身高与体重测量）** | | | | | | | | |
| PE.3.1 身高 **□□□.□**cm 躯干不直 □_1._是 □_0._否 | | | | | | PE.3.2 体重**□□□.□**kg | | |
| **（结束调查）** | | | | | | | | |
| 调查员印象：  □_1_受访者理解问题良好 □_2_受访者理解问题一般 □_3_受访者理解问题较差 | | | | | | | | |
| **调查员自检问卷，确认无漏填问题 □**  结束时间：**□□:□□**  调查员签名： | | | | | | | | |

审核员审核日期：**2012**年**□□**月**□□**日，时间**□□:□□**

审核员签字： ______________________
