# Supplementary material for: The Effects of a Community-Based Sodium Reduction Program in Rural China – A Cluster-Randomized Trial
Source: PLoS One. 2016 Dec 9;11(12):e0166620. doi: 10.1371/journal.pone.0166620 (PMC5147834; doi:10.1371/journal.pone.0166620)
Supplement: S2 File — (DOC) [file pone.0166620.s004.doc]

**CRHI Survey Questionnaire（2012）**

Address to the interviewee (refer to the Survey Training Manual). Ask Interviewee if they have brought their ID and medication containers, and if they satisfy the measurement requirements.

Date (2012/mm/dd) **2012/□□/□□** Time (hour: minutes) **□□:□□**

| **A. General information** | | | | | | | |
| --- | --- | --- | --- | --- | --- | --- | --- |
| A.1 Name:______________________ A.2 Sex: □_1._Male □_0._Female  A.3 ID**□□□□□□□□□□□□□□□□□□**  A.4 Age in years______________ A.5 Years of schooling ________________  A.6 During the past one year, how long have you lived in the village? ______________months | | | | | | | |
| **B. Lifestyle information in the past 3 months** | | | | | | | |
| B.1 During the past 3 months, have you smoked? (multiple choice)  □_1._Yes, cigarette B.1.1 On average, how many cigarettes per day __________  □_2_.Yes, tobacco leaf B.1.2 On average, how many “liangs (equivalent to 50g)” per day ____________  □_3._Other (go to B.2)  □_0_ No (go to B.2) | | | | | | | |
| B.2 During the past 3 months, have you drunk any alcohol? (multiple choice) | | | | | | | |
| □_1_. Yes | | | | | □_0_. No (go to B.3) | | |
| B.2.1How often?  □_1_. <Once/week  □_2_. 1-2times/week  □_3_. 3-5times/week  □_4_. 1-2times/day  □_5_. >Twice/day | | | | |  | | |
| B.2.2 During the past 3 months, how many times did you drink alcohol exceeding more than 5 “liangs”? __________ (note:1 liang=50g≈50ml ) | | | | |  |  |  |
| B.3 During the past 3 months, have you done any physical activities? (farming included, housework excluded) （minimum 30 minutes each time） | | | | B.4 During the past 3 months, have you performed any exercise? （minimum 30 minutes each time）  (e.g., walking、running、dancing, Taichi etc.） | | | |
| □_0._Hardly ever  □_1._1-2days/week  □_2 ._3-5days/week  □_3_. Almost daily | | | | □_0._Hardly ever  □_1._1-2days/week  □_2 ._3-5days/week  □_3_. Almost daily | | | |
| **Physical examination (PE.1). Blood pressure and heart rate measurement (1st)** | | | | | | | |
| PE.1.1 Blood pressure（Systolic/Diastolic）:  PE.1.1.1**□□□**/ PE.1.1.2**□□□** mmHg | | | | PE.1.2 Heart rate**□□□**/minute | | | |
| B.5 During the past 3 months, how often have you eaten pickled foods?  □_1_Hardly ever  □_2_Occasionally  □_3_1-2 times/week  □_4_3-6 times/week  □_5_ At least once a day | | | | B.6 During the past 3 months, what did you do when you found the food was not salty enough to your taste?  □_1_ Always eat pickles/add soy sauce/add salt  □_2_ Sometimes eat pickles/add soy sauce/add salt  □_3_ Did not eat pickles/add soy sauce/add salt | | | |
| B.7 High salt intake will:  □_1_ worsen your health  □_2_ improve your health  □_3_ have no affect on  your health  □_4_ Not sure | B.8 Which of the following can help reduce blood pressure? (read out one by one)  B.8.1Medicine: □_1_ Yes □_0_ No□_9_Don’t know  B.8.2Smoking: □_1_ Yes □_0_ No□_9_Don’t know  B.8.3Eating less salt: □_1_Yes □_0_ No □_9_Don’t know  B.8.4Eating more food: □_1_Yes □_0_ No □_9_Don’t know | | | | B.9 What is the daily recommended salt intake for adults?  □_1_No salt  □_2_<6g salt  □_3_>6 g salt  □_4_ Not sure | | |
| B.10 Do you pay any attention to reducing your salt intake in your daily diet?  □_1_ A lot of attention (have eliminated pickled vegetables from diet, add less salt and no MSG to cooking, if foods are too salty will not continue to eat)  □_2_ Some attention (decreased intake of pickled vegetables, add less salt during cooking, if foods are too salty will make sure to eat less)  □_3_ No attention (have not decreased the intake of pickled vegetables or salt in any way) | | | | | | | |
| B.11 Does your household use low-sodium salt? *(Investigator should show interviewee one packet of low-sodium salt .that is available in local stores If the interviewee does not know the answer, the investigator should make every effort to obtain the following information from another member in the same household before ticking ‘Don’t know’)*  □_1_ Yes   | B11.1 What is the proportion of low-sodium salt consumption among total salt intake in your household?%  *(The investigator should make an estimate after detailed inquiry of the household’s salt intake, for instructions on inquiry and estimation, please refer to the Instruction Manual on how to complete the questionnaire.)*  B11.2 When did your household start using low-sodium salt?  □_1_ year month □_9_ Don’t know  B11.2.1 Since then, have you been consistently using low-sodium salt up to now?  □_1_ Yes  □_2_ No; total time not using low-sodium salt? Months  *(If don’t remember, then fill in the blank with “999”.)*  □_9_ Don’t know | | --- |   □_2_ No   | B11.3 Why do you not use a low-sodium salt?（can choose more than one answer）  □_1_Can’t buy it nearby □_2_ Too expensive □_3_ Not very tasty □_4_ Feel not effective  □_5_Had side-effects after eating *（please explain）*□_6_ Don’t know  □_7_Worried about side-effects □_8_No interest □_9_Never heard of “low-sodium salt” □_10_Other *（please explain）* | | --- |   □_3_ I don’t know what kind of salt is used in my household. | | | | | | | |
| **C. Disease history** | | | | | | | |
| C.1 Do you have coronary heart disease?  □_1_. Yes □_0_. No □_9_. Don’t know (if No or Don’t know, go to C.4)  C.1.1 Diagnosed at a county or higher level hospital?  □_1_. Yes □_0_. No □_9_. Don’t know  C.1.2 Did the doctor tell you that your ECG was abnormal?  □_1_. Yes □_0_. No □_3_. Did not have ECG done □_9_. Don’t know  C.1.3 When was it first diagnosed?  ________Year_______Month  C.1.4 When was the most recent onset?  ________Year_______Month  C.2 Have you ever had a stroke?  □_1_. Yes □_0._No □_9_. Don’t know (if No or Don’t know, go to C.3)  C.2.1 Diagnosed at a county or higher level hospital?  □_1_. Yes □_0_. No □_9_. Don’t know  C.2.2 Was it an ischemic stroke or hemorrhagic stroke?  □_1_. Ischemic stroke □_2_. Hemorrhagic stroke □_3_. Don’t know  C.2.3 When was it first diagnosed?  ________Year_______Month  C.2.4 When was the most recent onset?  ________Year_______Month  C.3 Do you have diabetes?  □_1_. Yes □_0_.No □_9._Don’t know (if No or Don’t know, go to C.2)    C3.1 Diagnosed at a county or higher level hospital?  □_1_. Yes □_0_. No □_9_. Don’t know  C3.2 When was it diagnosed?  ________Year_______Month  C.4 Do you have hypertension (HTN)?  □_1_. Yes □_0_. No □_9_. Don’t know (if No or Don’t know, go to C.5)  C.4.1 How many years have you had hypertension (HTN)? ________ Years □_9_. Don’t know  C.4.2 Was the HTN diagnosed before 10/2010? □_1_. Yes □_0_. No □_9_. Don’t know  C.5 Have you had your blood pressure measured after age 50 (male)/60 (female)?  □_1_. Yes □_0._No □_9_. Don’t know (if No or Don’t know, go to C.6)  C.5.1 Please provide the highest systolic blood pressure measured **□□□**mmHg (If Don’t know, fill as 999) | | | | | | | |
| C.6 During the past two weeks, have you been taking any anti-hypertensive medication?  □_1_. Yes □_0_. No □_9_. Don’t know | | | | | | | |
| C.7 During the past one year, have you had any of the following symptoms or illnesses? (read out one by one, can choose more than one answer)  □_1_ Dizziness □_2_. Headache □_3_. Weakness □_4_. Fall □_4_. Gingival bleeding or subcutaneous hemorrhage □_5_ Stomachache □_6_. Gastrointestinal bleeding confirmed by a doctor □_7_. Cerebral hemorrhage confirmed by a doctor□_8_. Hyperkalemia □_9_. Hypoglycemia confirmed by a doctor (Lowest blood glucose level _______mmol/L) □_10_ Hypotension confirmed by a doctor (Lowest blood pressure value ___/___mmHg) □_11_Heart failure confirmed by a doctor □_12_ Renal insufficiency confirmed by a doctor □_13_ Any life-threatening condition requiring hospitalization or prolonging hospitalization (describe condition ____________________) □_14_Other diseases confirmed by the doctor (describe______________________） | | | | | | | |
| C.8 During the past 12 months, did you see your village doctor once a month?  □_1_. Yes  □_0_. No | | C.9 Did your village doctor measure your blood pressure at each visit?  □_1_. Never saw village doctor □_2_. Never measured  □_3_. Sometimes(occasionally) □_4_. Almost every time  □_5_. Every time | | | | | |
| **Physical examination (PE.2). Blood pressure and heart rate measurement (2nd)** | | | | | | | |
| PE.2.1 Blood pressure（Systolic/Diastolic）  PE.2.1.1**□□□**/ PE.2.1.2**□□□** mmHg | | | | | PE.2.2 Heart rate:  **□□□**/minute | | |
| | **CL．CVD high-risk patient confirmation checklist (CL) for the interviewer** | | | --- | --- | | CL.1 Has reported a history of diabetes（C.1.1 answer Yes） | □_1._Yes □_0._No | | CL.2 Has reported a history of stroke（C.2.1 answer Yes） | □_1._Yes □_0._No | | CL.3 Has reported a history of CHD（Both C.3.1 and C.3.2 answer Yes） | □_1._Yes □_0._No | | CL.4 Has reported a history of SBP ≧ 160mmHg with older age （C.5.1） | □_1._Yes □_0._No | | CL.5 Systolic BP measurement ≧160mmHg **twice** （PE.1.1 and PE.2.1） | □_1._Yes □_0._No | | | | | | | | |
| **If the answer to any of the above questions is Yes, continue the survey; otherwise go to PE.3 to measure participant’s height and weight** | | | | | | | |
| **D. Medication use and medical care (high-risk patients ONLY)** | | | | | | | |
| D.1 When was the last time that you saw your village doctor for heart disease, stroke, diabetes, or HTN? _______days ago  During that visit, did the village doctor provide any of the following recommendations?  D.1.1 Reduce salt intake □_1._Yes □_0._No  D.1.2 Quit smoking □_1._Yes □_0._No □_9._Not applicable  D.1.3 Avoid binge drinking □_1._Yes □_0._No □_9._Not applicable  D.1.4 Be physically active □_1._Yes □_0._No  D.1.5 Control weight □_1._Yes □_0._No □_9._Not applicable | | | | | | | |
| D.2 During the past 12 months, have you been hospitalized due to heart disease, stroke, diabetes or hypertension?  (exclude hospitalization for other reasons) □_1._Yes □_0._No (If no, go to D.3)  D.2.1 How many times have you been in the hospital? __________（if no，go to D.5）  D.2.2 How many days have you been in the hospital? __________  D.2.3 How much did you spend in out of pocket expenses for the hospitalization? __________ RMB | | | | | | | |
| D.3 During the past 12 months, how many months have you taken anti-hypertensive medication (AHM)?  □_0._None  □_1._Not sure if the medication taken was AHM  □_2._＜2 months  □_3_ 2-8 months  □_4_ 9-11 months  □_5_ =12 months  （if none or not sure, go to D.4） | | | D.3.1Who prescribed the AHM for you?  □_1_. Self prescribed/bought  □_2_. TCM doctor  □_3_. Specialist (town or higher level)  □_4_. Village doctor and specialist  □_5_. Village doctor | | | | D.3.3 Where do you buy AHM?  □_1_. Village clinic  □_2_. Township center  □_3_. County hospitals  □_4_. Pharmacies |
|  |  |  | D.3.2. Has the village doctor ever adjusted the treatments, either titrating the dosage or changing the prescription for you?  □_1._Yes □_0._No □_9._Dont’ know | | | | |
| D.4 During the past 12 months, how many months have you taken aspirin?  □_0._None  □_1_.Not sure if the medication taken was Aspirin  □_2_.＜2 months  □_3_. 2-8 months  □_4_ 9-11 months  □_5_ =12 months  （if not a long-term user or don’t know, go to D.5） | | | D.4.1 Who prescribed the aspirin?  □_1_ Self prescribed/bought  □_2_. TCM doctor  □_3_. Specialist  □_4_. Village doctor and specialist  □_5_. Village doctor | | | D.4.2 Where do you buy aspirin?  □_1_. Village clinic  □_2_. Township center  □_3_. County hospitals  □_4_. Pharmacies | |
| D.5 During the past month, have you taken any medications for your heart disease, stroke, diabetes or hypertension?   | □_1._Yes □_9._Don’t know | □_0._No (if No, go to PE.3 to measure height and weight) | | --- | --- |  | D.5.1 Medicine (brand name) | D.5.2 Taken daily?  (>25days in the last 30 days) | D.5.3 Prescribed by a village doctor? | | --- | --- | --- | | 1. | □_1._Yes □_0._No | □_1._Yes □_0._No | | 2. | □_1._Yes □_0._No | □_1._Yes □_0._No | | 3. | □_1._Yes □_0._No | □_1._Yes □_0._No | | 4. | □_1._Yes □_0._No | □_1._Yes □_0._No | | 5. | □_1._Yes □_0._No | □_1._Yes □_0._No | | If more than 5, and if the drug category is uncertain, copy the names of the remaining medications in the blank field below and give the numerical answers for D5.2 and D5.3. 1=Yes, 0=No | | |  | **Physical examination (PE.3). Body height and weight measurement for all survey participants** | | | --- | --- | | PE.3.1 Height **□□□.□** cm  Body straight? □_1._Yes □_0._No | PE.3.2 Weight **□□□.□**kg |  | **（End of survey）** | | --- | | | | | | | | |
| Interviewer comments/impressions:  □Interviewee understands questions well  □Interviewee understands questions partly  □Interviewee understands questions poorly | | | | | | | |
| Time of completion：**□□:□□**  Interviewer double check and sign  Interviewer signature： | | | | | | | |

Date of quality control review: 2012 __ month__ day time: **□□:□□**

Quality control reviewer signature: _____________________
