# Supplementary material for: The Effects of a Community-Based Sodium Reduction Program in Rural China – A Cluster-Randomized Trial
Source: PLoS One. 2016 Dec 9;11(12):e0166620. doi: 10.1371/journal.pone.0166620 (PMC5147834; doi:10.1371/journal.pone.0166620)
Supplement: S3 File — (DOC) [file pone.0166620.s005.doc]

**中国农村健康行动**

——中国农村心脑血管疾病防控方案效果评价研究

**研究方案**

### 中国国际慢性病预防中心

### 该中心由以下机构通力合作

| **中方合作机构：** | **国际合作机构：** |
| --- | --- |
| **中国乔治中心**  **北京大学医学部**  **中国医科大学**  **西安交通大学医学院**  **河北省疾病预防控制中心**  **宁夏医学院**  **长治医学院** | **美国杜克大学**  **澳大利亚乔治全球健康研究中心**  **澳大利亚悉尼大学**  **澳大利亚昆士兰大学**  **英国伦敦帝国学院** |

### 由以下机构联合资助：

**美国国立卫生研究院心肺血研究所**

**美国联合健康集团**

2010年5月20日

**目录**

[1. 研究目的 4](#__RefHeading___Toc262471350)

[**1.1** **主要目的：** 4](#__RefHeading___Toc262471351)

[**1.2** **具体目的：** 4](#__RefHeading___Toc262471352)

[2. 背景和意义 4](#__RefHeading___Toc262471353)

[**2.1** **背景** 4](#__RefHeading___Toc262471354)

[2.1.1 中国农村地区的心脑血管疾病负担 4](#__RefHeading___Toc262471355)

[2.1.2 中国农村医疗保健体系 5](#__RefHeading___Toc262471356)

[2.1.3 中国高血压流行现状 5](#__RefHeading___Toc262471357)

[2.1.4 中国农村地区目前的心脑血管疾病管理模式 6](#__RefHeading___Toc262471358)

[2.1.5 培训村医的效果 6](#__RefHeading___Toc262471359)

[2.1.6 药物干预措施 6](#__RefHeading___Toc262471360)

[2.1.7 限盐措施 7](#__RefHeading___Toc262471361)

[**2.2** **以前的工作基础** 7](#__RefHeading___Toc262471362)

[2.2.1 以社区为基础的心脑血管疾病简化管理模式研究 7](#__RefHeading___Toc262471363)

[2.2.2 《高血压防治基层实用规范》的制订与应用效果评价 7](#__RefHeading___Toc262471364)

[2.2.3 限盐项目的可行性研究 8](#__RefHeading___Toc262471365)

[**2.3** **本研究的意义** 12](#__RefHeading___Toc262471366)

[3. 研究设计 12](#__RefHeading___Toc262471367)

[3.1 省，县，乡镇和村的选取 12](#__RefHeading___Toc262471368)

[3.2 随机方法 13](#__RefHeading___Toc262471369)

[3.3 干预和对照 13](#__RefHeading___Toc262471370)

[3.4 评价指标 15](#__RefHeading___Toc262471371)

[3.5 评价方案 16](#__RefHeading___Toc262471372)

[3.6 统计功效 18](#__RefHeading___Toc262471373)

[3.7 分析计划 18](#__RefHeading___Toc262471374)

[4. 时间安排 18](#__RefHeading___Toc262471375)

[5. 伦理和知情同意 19](#__RefHeading___Toc262471376)

[6. 参考文献 20](#__RefHeading___Toc262471377)

[7. 附件 22](#__RefHeading___Toc262471378)

[7.1 附件1：以村医为基础的心脑血管疾病防控技术方案详细内容及心脑血管疾病筛检、管理、与随访流程图（见单独附图） 22](#__RefHeading___Toc262471379)

[7.2 附件2：心脑血管病高危人群病例管理记录表 26](#__RefHeading___Toc262471380)

[7.3 附件3：效果评价（基线/复查）调查表 29](#__RefHeading___Toc262471381)

[7.4 附件4：中国农村健康行动以村医为基础的心脑血管病防治干预计划知情同意书 31](#__RefHeading___Toc262471382)

[7.5 附件5：心血管高危患者病例记录管理知情同意书 35](#__RefHeading___Toc262471383)

[7.6 附件6：效果评价（基线/复查）被访对象知情同意书 38](#__RefHeading___Toc262471384)

# 研究目的

- 1. **主要目的：**

本项研究的**主要目的**是制定、实施并评估一种有效、低成本、可负担，且适宜中国广大农村地区的心脑血管疾病预防管理方案。

- 1. **具体目的：**

1.2.1制定并实施一种简单有效、低成本，并由村医执行的心脑血管疾病预防管理技术方案（此后称为PCP），探索该技术方案是否能够有效提高心脑血管疾病高危患者（此后称为“高危患者”）的管理率和血压控制状况。

1.2.2开展一项以乡镇健康教育专员为依托、以降低高危人群及普通人群钠摄入量为目标的健康教育和健康促进方案（此后称为HE），探索该方案是否能够有效降低高危患者平均食盐摄入量。

# 背景和意义

- 1. **背景**

无论在发达国家还是在发展中国家，心脑血管疾病都已成为居民的最主要死亡原因之一^1^。2005年，全世界死亡人数约为5800万人，其中约30%死于心脑血管疾病^1^。世界卫生组织1999年报告指出约85%的心脑血管疾病负担来自于发展中国家^2^，且发展中国家接近一半的心脑血管疾病死亡都发生在70岁以下，而在发达国家该比例为1/4^3^。由此可见，心脑血管疾病已给发展中国家造成沉重的疾病负担，探索适用于发展中国家的心脑血管疾病预防管理策略措施是目前所急需解决的世界卫生问题。

## 中国农村地区的心脑血管疾病负担

心脑血管疾病是中国居民的首要死因，2008年全国死于心脑血管疾病的人数约为350万人，占总死亡人口的38%^4^。我国的心脑血管疾病模式与西方国家不同，脑卒中的发病率和患病率要高于冠心病^5^。另外，心脑血管疾病发病率存在地理差异，北方地区的发病率要高于南方地区^5^。心脑血管疾病有众多的危险因素，其中，高血压是我国居民最重要的危险因素^6^。我国北方农村地区居民的高血压患病率，食盐消费量以及脑卒中发病率均处于较高水平。

## 中国农村医疗保健体系

中国约有8亿居民生活在农村地区。在广大农村地区，医疗卫生保健体系一般分为3级：县级、镇级和村级。县级包括县医院和县疾病预防控制中心，镇级为乡镇社区卫生服务中心，村级为村诊室。村诊室由乡镇社区卫生服务中心直接管理培训，同时，乡镇社区卫生服务中心由县卫生局直接管理，并接受县医院和县疾病预防控制中心的技术培训和指导。在中国农村地区，每1000名村民中，平均仅有1.2名医疗卫生人员为其提供医疗卫生服务^4^。

在中国农村地区，每个村至少有一名具有行医执照的村医，大部分村医都具有国家基本药物目录中药物的处方权。村诊室一般由1-2间屋子构成，具备少量的基本医疗器械。在有些村，经常会有一名女性卫生保健员协助村医，主要负责妇幼保健方面的工作。一般情况下，村医和卫生保健员也都居住在村里。

## 中国高血压流行现状

“2002年中国居民营养与健康状况调查”结果显示，中国农村地区高血压患病率接近于城市地区，分别为17%和21%，然而农村地区的高血压知晓率、治疗率和控制率则显著低于城市地区^7^。有证据表明，利用低成本的降压药物（如利尿剂）能够有效地控制大多数高血压患者的血压水平。同时，健康的生活饮食习惯也有益于帮助高血压患者控制血压水平。

中国已经制订了《高血压防治指南》，为高血压的诊断、治疗和管理提供了详细指导。然而，如同其他国家一样，该指南并没有在全国范围内尤其是在农村地区得到应用，我们还需要提取其中的关键部分并进一步推广。在农村地区，对指南的简化和提炼更为重要，因为村医的能力和乡村医疗卫生的条件都十分有限，让他们掌握或实践指南中的所有内容是不现实的。

现有指南的另一个作用是能够确保农村地区稀缺的医疗资源能够得到最有效的利用。指南中提出了心脑血管疾病发病风险的评估方法，根据评估结果决定哪些高危人群需要优先得到治疗。一系列的研究表明，依据多个危险因素计算出心脑血管疾病发病风险，并依据此结果确定需要降压的高危人群，此方法要比仅依据个体血压水平而决定是否需要降压治疗更为有效。这就意味着，有的高血压患者其血压水平刚过临界值（140/90 mmHg），且没有其他危险因素，其心脑血管疾病发病风险可能要低于那些未患高血压但具有其他危险因素的患者（如糖尿病患者血压应控制在130/80mmHg以下），在医疗资源有限的情况下，应该优先治疗后者。

## 中国农村地区目前的心脑血管疾病管理模式

目前，在村级水平，几乎没有关于心脑血管疾病的管理策略措施，国家制定的临床高血压和心脑血管疾病防治指南也未能推广到农村地区。

农村地区心脑血管疾病最重要的危险因素是高血压和吸烟。另外需要考虑的是，中国目前的乡村-城市的迁徙流，越来越多的农民来到城市寻找工作机会，而一旦患心脑血管疾病，他们又将回到乡村接受长期治疗。农村地区医疗人员能力有限，而心脑血管疾病发病率和死亡率却又不断上升，目前急需对医疗人员和患者进行双重干预以遏制该趋势的发展。

## 培训村医的效果

有研究指出，对村医进行培训能够提高他们的行医能力和技巧，并能够增强村医对于提高自身能力的积极性^24^。但是，对基层医生进行高血压相关知识的培训，是否能降低其所诊治的患者平均血压水平尚无明确的结论。大部分研究认为对基层医生进行培训并不能够降低患者的平均血压水平，需同时对高血压患者进行健康教育，方能有效地降低平均血压水平^25-27^。我们自己的前期工作表明，针对中国农村村医的知识水平和专业受训水平，制定适宜的技术规范，在技术培训的基础上辅以必要的管理，能够显著提升被管理患者的高血压控制率。

## 药物干预措施

来自于大规模临床随机试验的结果表明，许多药物干预措施可以用于脑卒中的预防，如降压和抗血小板治疗，这些干预措施对于高危患者的效果更加显著。另外，目前中国的医疗保障体系已能够支付得起一些广泛应用的非专利药物，这些药物疗法具有降低中国农村地区心脑血管疾病发病率、死亡率和经济负担的潜力。

## 限盐措施

观察性研究^8^ 和随机临床试验^9,10^均已证实减少食盐的摄入能够降低血压水平^10, 11^。 同时，血压的下降又能降低心脑血管事件的发病风险^12, 13,14^。然而，还没有直接的证据证明限盐能够降低心脑血管疾病的发病率和死亡率^8, 15-19^。最近，世界卫生组织发表了一篇技术报告，推荐所有会员国制定全国性的限盐方案^20^。

在中国北方农村地区，限盐措施的效果可能要高于世界其他任何地方。因为，生活在我国北方农村地区的居民食盐摄入量非常高^21, 22^，且绝大部分食盐摄入来自于家庭烹饪，这使得限盐项目的开展更加容易、便宜，也更值得去做^23^。

- 1. **以前的工作基础**

## 以社区为基础的心脑血管疾病简化管理模式研究

2009年，我们与加拿大多伦多大学合作开展了一项预研究，目的是了解中国社区初级医疗卫生服务机构心脑血管疾病的管理质量，提出社区心脑血管疾病管理的简化模式。研究地点为4所社区卫生服务中心，其中北京市2所（1城市，1农村），河北省保定市2所（1城市，1农村）。采用定量调查法和定性调查法收集数据。城市地区心脑血管病高危人群高血压控制率为29.3% ，农村地区仅为13.9%。城市和农村地区降压药物（包括中药）的服用率分别为30.2%和30.6%，阿司匹林服用率分别为36.9%和31.4%。利尿剂的服用率也很低，城市和农村地区分别为6.9%和3.2%。

## 《高血压防治基层实用规范》的制订与应用效果评价

2001年，本项目负责人曾牵头组织一个专门的全国专家委员会，以中国高血压和心脑血管疾病临床指南为基础，起草了《高血压防治基层实用规范》。《规范》的起草工作得到了中华医学会心血管病分会、北京市高血压防治协会、全国心血管病防治研究办公室、世界卫生组织心血管病研究与培训合作中心及中国疾病控制中心的大力支持，其终稿以上述机构的名义于2002年10月在北京召开的“国际高血压及相关疾病学术会议”上向社会正式发布。之后，我们在北京4个社区（2个农村和2个城市）进行的为期1年的随机干预试验（560名受试患者），干预1年后结果表明，城市干预组患者血压控制率（<140/90mmHg）为75.2%，对照组患者控制率为40.3%（p<0.01）。农村干预组患者血压控制率为77.9%，对照组患者控制率为26.4%（p<0.01）（表）。在1年的干预期内，高血压患者用于购买降压药物的年平均花费，在城市干预组为569.4元，明显低于对照组766.5元，（p<0.05）；在农村干预组为295.6元，也低于对照组354.1元，（p>0.05）但差异未达到统计显著性。

2003年，卫生部疾病控制司批准在全国推广使用《高血压防治基层实用规范》，并将文件下发至各省卫生厅。推广一年内即有十余家省市级卫生行政部门包括疾控中心、卫生局、初级保健基金会、心血管病防治办公室等购买《规范》用于推广，并在7座城市成功举办培训班，累计培训学员1800余人。截至目前，《规范》推广和受训基层医生累计已超过3万人。

表1 北京市城乡社区患者不同随访时期血压控制率（%）情况

血压控制情况城市社区农村社区干预组对照组干预组对照组入选0000随访3个月49.445.835.118.4随访6个月64.350.9*40.323.9*随访9个月70.452.1*62.022.0**随访12个月75.240.3**77.926.4**与干预组相比：* p<0.05；** p<0.01。

## 限盐项目的可行性研究

1988年，本项目负责人曾在中国陕西汉中农村的16户（共38人）农民中开展了一项旨在评价减盐措施在农民中实施的可行性及效果的小型干预试验。结果表明：经过12周的干预，膳食盐摄入量由干预前的平均11.1±3.7g／日下降到6.1±1.7g／日，平均下降了5.0g／日。其中来自高盐食物（如咸菜、泡菜、豆豉等）的盐平均下降了3.9g。8小时夜尿钠及钠／钾比值也相应下降，但无统计学显著性。干预第4周后，血压出现显著性下降，第8周、第9周达最低点。与干预前相比，干预后收缩压平均下降1.7kPa（12.5mmHg），舒张压平均下降1.0kPa（7.8mmHg），P值均＜0.01）。上述结果说明：在足够的干预强度下，简单、明确、具体的膳食减盐指导可以有效帮助农民减少食盐摄入。遗憾的是，当时条件所限未能设立平行对照。

2004年，我们从中国北方6个农村地区的乡镇中选取了608名心脑血管病高危患者。这608名患者被随机分配至代用盐组（低钠高钾盐）和普通盐组。干预12个月后，代用盐组患者的收缩压平均水平比普通盐组净降低5.4mmHg（95%置信区间为2.3-8.5mmHg），且两组平均血压差值随着干预时间的延长而增加（*P*=0.001）。在研究期间，代用盐组中有98%的患者坚持使用代用盐作为他们平时的食用盐。

图1 血压平均值随时间的变化情况

2005年，我们在北京农村的220名高血压指示病例及其348名家庭成员中再次进行了随机双盲代用盐干预试验。干预1年后，高血压病人的门诊收缩压、家庭收缩压和动态收缩压分别下降4.1（0.8-7.4）mmHg、3.1（0.4-5.8）mmHg和2.4（-2.4~5.7）mmHg；2级高血压降压幅度大于1级高血压。家庭成员家庭收缩压下降1.5（-0.4~3.4）mmHg，其中高血压病人收缩压下降2.7（-1.9~7.2）mmHg，血压正常者下降0.5 (-1.2~2.1) mmHg；并且年龄越大，降压幅度越大。进一步分析发现，代用盐组服药率下降程度大于对照组，第3个月差别显著（p<0.05）。试验期间，试验组100%“全部”或“多半”食用代用盐，对照组98.2%“全部”或“多半”食用普通盐，两组的依从性没有显著差异（p>0.05）。

2009年，我们在中国西藏地区的牧民中开展了为期3个月的随机单盲代用盐干预试验，入选284名高血压患者。结果表明：

在为期3个月的干预后，与对照组相比代用盐组平均收缩压和舒张压水平都有显著下降。在调整年龄、性别、BMI、基线和随访的药物应用情况、基线收缩压或舒张压后，收缩压和舒张压的净差别代用盐组为-8.8 mmHg (95% CI: -19.6 to -1.0)，对照组为-4.5 mmHg (95% CI: -8.5 to -0.6)。

基线

随访

血压水平 (mmHg)

80

90

100

110

120

130

140

150

160

170

180

190

200

对照组

代用盐组

△: -15.5 mmHg

(95% CI: -19.2 to -11.7)

△: -6.9 mmHg

(95% CI: -10.9 to -3.0)

△: -6.3 mmHg

(95% CI: -8.3 to -4.2)

△: -3.0 mmHg

(95% CI: -5.0 to -0.9)

净差别：-8.8 mmHg

(95% CI: -16.9 to -1.0)

净差别：-4.5 mmHg

(95% CI: -8.5 to -0.6)

SBP*

DBP*

图2 经过多因素调整后，对照组与代用盐组平均收缩压和舒张压水平情况

- 1. **本研究的意义**

中国农村地区正遭受着严重的心脑血管疾病负担，而许多由心脑血管疾病所造成的死亡、残疾本是可以避免的。许多行之有效的高危人群干预措施如降压、减少食盐摄入、给予阿司匹林、定期随访管理等已得到大量科学研究的证实，然而这些干预措施的综合运用效果，以及在用于缺医少药的中国农村地区的心脑血管疾病防治时的效果如何，能否大范围推广，尚需大型科学研究来获取相关证据。这项大规模整群随机对照试验将准确评价这些干预措施的效果，其结果将为未来相关政策的制定提供科学可靠的证据。

# 研究设计

本研究将是一项大规模整群随机对照试验，采用2x2析因设计。所评价的2个措施分别为：1）PCP：以村医为基础的心脑血管病高危人群预防管理技术方案，和2）HE：以乡镇健康教育专员为依托、以降低高危人群及普通人群钠摄入量为目标的健康教育和健康促进方案。

## 省，县，乡镇和村的选取

选取与我们有合作基础的河北，辽宁，宁夏，山西和陕西5省为研究地点。每个省辖13-136个县，每个县辖12-22个乡镇。这些省均有较高的心脑血管疾病发病率和高血压患病率。根据各县相关领导和工作人员参与本项目的积极性、与各地项目负责人的亲密关系以及经济发展水平，从每个省中选取2个县参与到本研究中。每个县选取12个乡镇参与本研究。乡镇的选择将根据选定县卫生行政当局的意愿以及交通便利情况等因素最终确定。从每个县选取的12乡镇将被随机分配进入干预组和对照组。

每个乡镇将选取一个村进入本研究，位于乡镇中心位置的村是我们的首选，但不能选择乡镇卫生院所在的村。这样做可以防止相邻的村被同时选中且进入不同的干预组或对照组的情况发生，从而可以避免临近村落之间产生污染。同时避免因乡村规模大小和卫生资源不同导致的组间不平衡，以及避免对乡镇卫生院众多医生的复杂管理。

在整个入选过程中，乡镇和其中选出的村不能决定其进入干预组还是对照组。这要由随机方案来确定。但如果被选取村的村医不愿参加本研究，可放弃其所在的村，邀请另外一个最接近乡镇中心位置的村参加。

## 随机方法

本研究采取分层区组随机，以乡镇将为随机单位，按照县级进行分层。将所有入选的120个乡镇先按照10个县分成10组，每组12个乡镇，再按照相邻关系将12个乡镇分为3个区组，每个区组4个乡镇。采用完全随机方案将4个乡镇分配分别进入4个不同干预组：PCP+HE组、单独PCP组、单独HE组和完全常规干预组。如此，将有60个乡镇最终得到PCP干预，60个乡镇无PCP干预；60个乡镇最终得到HE干预，60个乡镇无HE干预；30个乡镇最终得到联合干预，30个乡镇完全常规干预。

选择乡镇而不是乡村作为随机单位的主要考虑是：同一个乡镇的所有村医在技术上（甚至是行政上），特别是公共卫生和健康教育受同一乡镇卫生院的管理和指导，他们经常会见面并互相交流。如若在同一个乡镇纳入多个村庄进入不同的干预组和对照组，将会增加组间污染的发生风险。

仅选择乡镇中的1个村而不是所有村进行研究主要是受到研究经费和资源的限制。未来资源的进一步投入将会使研究的规模很容易在上述抽样和随机框架里扩展。

## 干预和对照

INTERVENTION

整个干预时间为2年。首先开展的是PCP干预，HE干预有待相关技术方案明确后开始，估计大约在PCP开始6个月后启动。

### PCP干预

随机分配到PCP干预组的乡村中的所有村医将接受PCP方案技术培训，并在培训后按照PCP方案的要求检出、分类、治疗和随访管理心脑血管病高危人群。符合以下3种情况之一者，定义为心脑血管疾病高危患者（不考虑目前用药情况）：

- 具有明确的冠心病或脑卒中病史者；或
- 年龄大（男性≥50岁，女性≥60岁）并且患有II型或I型糖尿病
- 年龄大（男性≥50岁，女性≥60岁）并且收缩压≥160mmHg。

PCP干预将包含以下内容：

- 村医接受PCP方案技术培训。我们将从每个县的县医院中邀请一名心内科专科医生作为本研究的技术培训专家，这些专家将在北京接受PCP相关技术培训以及学习如何培训村医的有关技巧。这些专家将负责培训本县被纳入PCP干预组的所有村医。所有村医须在通过考核后方可正式开始干预。
- 村医按照PCP方案要求检出、分类、治疗和随访管理心脑血管病高危人群。关于PCP的详细情况请见附件1。
- 村医为每一位高危患者建立“病例管理记录册（CMR）”。在高危患者首次就诊及之后的每次随访时，村医都需要填写CMR，记录患者的一般情况，症状，体征，诊断以及治疗情况（请见附件2）。
- 电子化中心数据库及PCP管理质量反馈。我们将在北京大学临床研究所数据管理中心建立一个用于本项目所有被干预高危人群的中心数据库及管理系统。每个县将聘请1名独立的监察员，除承担本研究的质量监查任务外，每2个月1次到村诊所把CMR的信息更新录入到中心数据库。中心数据管理系统除了对所有数据开展必要的质疑和质量控制外，每隔6个月对村医的医疗行为和PCP管理质量指标进行一次反馈，以提醒和促进村医对PCP的依从性。
- 经济激励。同时，我们将根据中心数据库和管理系统提供的村医医疗行为和PCP管理质量指标，给予村医小额经济激励。激励的项目主要是村医目前无法从现行规定中获益的预防性服务项目。如血压测量、患者定期随访、生活方式咨询、血压控制水平等。经济激励将与质量反馈同步，每半年一付。

### PCP对照

被随机分配至PCP对照组的村，村医将继续往常的医疗服务，也不接受由本项目组提供的任何与干预有关的技术培训，PCP干预不会被引入。

### HE干预

HE干预内容将是多方面的，可能包括的措施有：

1. 利用多种渠道和条件广泛传播心脑血管病的严重危害和少吃盐等科学防治知识。

- 利用村镇2级的广播电视系统
- 利用村里各处设立的宣传栏
- 利用村诊室内的宣传栏
- 发放宣传手册

1. 组织心脑血管病高危人群定期开展健康教育讲座和健康生活方式咨询。包括如何做出低盐好吃的饭菜。
2. 增加低钠高钾代用盐（65%氯化钠，25%氯化钾及10%硫酸镁）的可获得性。努力使村民有机会能够购买到代用盐。
3. 视经费情况采取对销售或购买代用盐补贴，促进和扩大代用盐的销售。

### 上述措施的有效实施将有赖于项目所在县的CDC、各乡镇健康教育专员、各村村医的密切合作以及县、乡、村三级政府的大力支持。详细的技术方案尚有待开发和完善。待此部分干预技术方案开发完善后，我们将再次提交伦理申请及相关的修改申请。

### HE对照

被随机分配至HE对照组的村，村医将继续往常的医疗服务，上述各项HE干预不会被引入对照村所在的乡镇。

## 评价指标

### 主要评价指标

两种干预的主要指标有所不同。

PCP干预的主要评价指标有2个：

1）被村医定期管理的高危患者比例；

2）高危患者的平均血压水平。

HE干预的主要指标为1个：

1）高危患者平均钠摄入量。

### 次要评价指标

PCP干预评价指标有：

- 在过去12个月内来过村医处测量血压及接受药物治疗的高危患者占所有高危患者的比例；
- 高危患者检出率；
- 高危患者服用降压药物和/或阿司匹林的比例；
- 高危患者采取任何生活方式治疗措施的比例；
- 高危患者血压控制率；

HE干预措施的评价指标有：

- 血压平均值；
- 全村人均每天食盐销售量。

### 相关定义

3.4.3.1效果评估调查中的高危患者定义为男性≥50岁，女性≥60岁且符合以下三种情况之一者：

①具有明确的冠心病、脑卒中、糖尿病病史者，或

②经测量收缩压≥160mmHg，或

③自报男性在50岁后，女性在60岁后曾经收缩压≥160mmHg。

3.4.3.2被村医管理：过去一年中，到村医处就诊达到9月次（指每个月至少1次，共9次）以上，且每次均测量血压并取与心脑血管疾病相关药物。

3.4.3.3平均钠摄入量：采用24小时尿钠反映钠盐摄入量。

3.4.3.4血压控制：测量时平均收缩压低于140mmHg。

## 评价方案

### 基线和干预结束后的随机抽样调查

基线和干预后将在所有120个干预和对照村中进行独立的随机抽样调查。抽样将由位于北京的中国国际慢性病预防中心负责进行。利用各村提前上报的居民花名册，首先找出所有50岁及以上男性和60岁及以上女性的名单。然后，分别给予每一位符合年龄的村民一个随机号码。将随机号码从小到大排列，从随机数字为1的村民开始进行知情同意，直至分别获得20名男性和20名女性适龄村民参加调查。调查内容将包括个人疾病史、用药史、生活饮食习惯以及体格测量（血压，身高，体重，心率等）。详见附件3。除调查样本各自独立外，干预前和干预后调查的抽样方案、调查表和调查测量方法将保持完全一致。

### 小样本24小时尿钠含量测定

为了评价HE干预对食盐摄入量的效果，我们将在上述参加基线和干预结束后的随机抽样调查的样本中再随机抽选20%的小样本（每个村8人，其中高危人群4人），收集其24小时尿样并运至位于北京的中心实验室测定尿钠、钾、及肌酐的含量。24小时尿样的采集、保存和运送方法及中心实验室的尿钠、钾、肌酐的测定方法另见“操作手册”。

### 发病率和死亡率监测

所有参加研究的村医（包括对照组的村医）都将要记录和上报本村的所有死亡及心血管病发病和死亡情况。有本研究聘请的监察员每季度收集1次，用“Verbal Autopsy”法确定死因。对发病资料采用改良的WHO-MONICA冠心病、脑卒中急性事件报表收集和记录相关资料。所有资料上报北京中心。由项目独立的事件诊断委员会作出最终诊断。县医院急诊和住院病历，以及县公安局的居民死亡登记信息均将被用来确定发病和死亡的原因。

### 高危人群病例管理记录（CMR）

60个PCP干预村将采用CMR管理高危患者，以帮助村医更好地理解并执行技术方案中的重要部分。CMR同时还将提供大量的第一手临床诊治信息，可以用来从各个角度评价干预组的效果，更全面地理解干预组起效的可能机制和有效环节。这对于今后推广和完善干预方案有十分重要的意义。

### 过程评价

过程评价对于复杂结局评价的重要性越来越得到认可^28^。通过分析以下几方面内容，过程评价能够更好地评估一项研究的结果及其价值：项目是否按照原计划进行，是否有其他因素影响项目的进行，各研究地点之间的干预实施情况是否存在差异，以及研究结果的推广价值如何等^29^。在本研究的过程评价中，研究人员将在基线和干预后收集所有村医的定性和定量数据，包括医疗保健知识和行医方式。在干预结束后，我们将对PCP干预组中村医的干预措施执行情况进行更深入的调查，同时，我们会对PCP干预组和HE干预组中相关的关键人员进行访谈，如县级卫生官员、乡镇卫生服务中心领导、培训专家、村医以及村民，以评价干预的执行情况。访谈内容将主要包括执行干预措施的经验介绍以及认为会对干预措施造成影响的因素。

### 经济学评价

经济学评价能够提供干预措施的成本效益分析，采用的方法将包括：

- 政府建立并运行该项目（包括支付给村医的经济激励）所需要的花费。相关数据将从本机构及合作单位的项目经费报告中获得。与研究相关的间接费用（如交通、餐饮等费用）将不纳入经济学评价。
- 由个人和由政府支出的医疗费用。患者的医疗保健费用数据将从每次随访记录中获得。考虑到各地医疗项目的收费情况存在差异，我们将根据市场标准价格对这些数据进行调整，计算出每名患者随访期间的平均花费，作为我们的主要分析变量。

我们将用“合理治疗每名患者所需要的费用”来进行成本效益分析。除此之外，我们还用以下方法进行分析：

- 对项目的初始成本进行合理分摊后，再做出不同的成本核算的敏感度分析。
- 基于以上成本效益分析结果，估算出节省每寿命年的费用和节省每伤残调整寿命年的费用。这些估算都将基于已有的关于疾病进展和长期治疗的证据。

根据以上分析结果，我们将能检验本研究中干预的成本效益是否符合世界卫生组织所制定的标准，即节省每伤残调整寿命年的费用应低于3倍的人均国民生产总值^30^。另外，我们还会进行灵敏度分析以评估研究结果的不确定性（来自于研究参数的变异）。

## 统计功效

### PCP干预

主要假设：α=0.05，入选120个群（干预组和对照组各60），群内相关系数为0.05，每个群内入选40名高龄（男50、女60岁以上）者，其中高危患者估计约为8名，收缩压标准差为15mmHg。在这些假设条件下，发现干预组和对照组平均收缩压5 mmHg的差异的统计功效>90%。

### HE

主要假设：α=0.05，入选120个群（干预组和对照组各60），群内相关系数为0.05，每个群内入选4人，24小时尿钠标准差为90mmol。在这些假设条件下，发现干预组和对照组平均24小时尿钠有50mmol的差异的统计功效 > 95%。如果每群内入选1名高危患者，则对于高危患者的统计功效为85%。

统计功效的计算建立在两种干预措施没有交互作用的假设前提之下。

## 分析计划

本研究主要分析干预组高危人群管理率、平均血压水平及每日盐摄入量与对照组的的差异，单独评价两种干预措施的效果。所有的分析都将调整群效应，并遵从Intention to Treat原则。

# 时间安排

两种干预措施将分两阶段实施，PCP项目的时间安排如下，HE项目的时间安排将视项目进展而定。

- 2010年05月 – 2010年07月：申请并通过伦理审批
- 2010年05月 – 2010年08月：制定标准操作手册
- 2010年09月 – 2010年10月：基线现场调查，培训村医
- 2010年10月 – 2012年10月：干预
- 2012年11月 – 2013年01月：干预结束后现场调查
- 2013年02月：项目结束并评估
- 2013年03月 – 2014年05月：数据整理，分析和报告撰写

# 伦理和知情同意

本项目将由北京大学和杜克大学伦理委员会联合审查。

由于本项目的干预措施的复杂性，需要分别获得个人知情同意和所在地区卫生行政部门的知情同意。为了取得各级卫生行政部门的知情同意研究小组将通过在各省的合作伙伴，首先向省级卫生行政机构说明并咨询。在取得其同意和支持后，再向县级卫生行政部门进行说明和咨询，并在后者的指导下确定参加本项目的乡镇名单。以此类推，直至村一级政府。知情同意的内容将包括对本项目的介绍和讨论。

调查中所使用的个人知情同意书将按照通常的模板来制定（详见知情同意书部分，共有三份，分别是中国农村健康行动以村医为基础的心脑血管病防治干预计划知情同意书—附件4，心血管高危患者病例记录管理知情同意书—附件5，及效果评价（基线/复查）被访对象知情同意书—附件6）。

# 参考文献

# 附件

## 附件1：以村医为基础的心脑血管疾病防控技术方案详细内容及心脑血管疾病筛检、管理、与随访流程图（见单独附图）

PCP干预是在以目前中国临床所使用的高血压和心脑血管疾病诊疗指南为基础，并进行简化提炼以使得该技术方案适用于中国农村地区。村医根据该技术方案筛检出一批心脑血管疾病高危患者，并根据PCP治疗和管理这些高危患者，并对其进行定期随访。我们将对干预组中的村医进行培训，并配备必要的医疗器械，如电子血压计等。该技术方案还包括医疗行为评估和反馈机制，并根据村医的表现给予相应的经济激励，以达到鼓励村医按照技术方案去治疗管理患者目的。

***筛检高危患者***

符合以下3种情况之一者，定义为心脑血管疾病高危患者（不考虑目前用药情况）：

- 具有明确的冠心病或脑卒中病史者；或
- 年龄大（男性≥50岁，女性≥60岁）并且患有II型或I型糖尿病
- 年龄大（男性≥50岁，女性≥60岁）并且收缩压≥160mmHg。

村医将通过测量血压以及询问病史和年龄的方式来筛检高危患者，所有来村诊室就诊的适龄患者都将接受筛检。另外，我们还会鼓励村医利用他们的关系以及对村民的了解，去主动寻找那些既往心脑血管疾病患者并纳入管理。

在筛检过程中，将可能会发现部分1级高血压患者（140≤收缩压＜160mmHg，或90≤舒张压＜100mmHg），我们将告知他们目前的病情并建议其去就医治疗，但这部分患者将不被纳入本研究的高危患者管理中去。

***高危患者的管理*** *–* 为了更好地实施干预措施，我们对村医进行培训以使得他们能够完成以下方面的工作：

- 向所有高危患者提供健康生活方式建议，其中主要关注降低食盐摄入和戒烟；
- 向高危患者提供合理正确（符合循证医学）的药物治疗，特别是积极降压和应用阿司匹林。
- 对这些高危患者进行定期随访和测量血压，以确保他们对干预措施的长期依从性和及时调整。

村医在遇到自身无法治疗的急重病人时，需立即转诊至上级医疗机构。我们将对村医进行培训，以使得他们能够合理正确地管理、随访和转诊高危患者，另外我们将会制作培训手册提供给村医以帮助他们强化和复习培训内容。（具体流程图请见另外提供的附件1-A和附件1-B）。流程图会以彩色海报的样式印制，将分发给各个参与干预方案的村卫生诊所，并贴在村医诊所办公室的墙上供其参考。流程图的相关解释：流程图之所以显得复杂的原因是因为1）为了利用危险评分公式从各种疾病患者中筛检和识别高危心血管病患者；2）为了清楚地区分哪些高危患者需要记录临床管理记录；3）为了强调高危患者需要被定期随访（循环管理）从而代替一次性就诊；4）为了能够让村医容易理解与掌握流程图从而帮助作出正确的临床决定。我们希望一旦村医接受了干预方案的培训，他们就能够根据所提供的流程图与技术操作手册解决大部分在临床治疗心血管病过程中遇到的问题。

作为PCP干预的一部分，高危患者病历管理记录将用于记录每名高危患者首诊、药物治疗、生活方式干预以及随访等数据，我们也将对村医进行相关培训以使得他们能够正确填写该病历管理记录。村医将为每名高危患者填写（手填）一份病历管理记录并保存在村诊室，该病历管理记录是13页开的册子，第一页为首诊记录表，后十二页为随访记录表（如果有患者的随访次数超过12次，则启用新的册子）。该病历管理记录不但能够标准化村医的医疗行为和模式，提高村医的医疗服务质量，还可以以此为依据对村医的医疗行为进行反馈。另外，在研究结束后，我们可以利用病历管理记录中的数据和信息来评价PCP干预措施的实际效果。

整个PCP干预将包含以下三大部分：技术培训，医疗行为反馈和经济激励。

- **技术培训**

所有参与干预项目的村医将会接受如何采用与实施简易心脑血管疾病防治干预措施的培训。 具体培训步骤如下：从各个县级医院里挑选至少一名心血管专家，统一安排在北京接受由项目组专家提供的为期2天的培训。培训内容主要除围绕干预措施的采用与实施外，还将涉及如何为其管辖的村医提供正确有效的培训。当县级心血管专家完成培训后，他们将负责为其所的参与干预方案的村医提供正确采用与实施心脑血管病防治干预措施的培训。

经过培训的县级医院的心血管专家可以自主地安排对村医的培训，我们鼓励专家积极采用病例讨论与角色扮演的培训模式。为村医开展的培训形式为一天的全天培训与不超过1天的回顾与复习。如果有必要，在培训后的一个月，研究组织方会为村医提供额外的培训复习与巩固。村医受训结束时，需要完成一份针对培训内容设计的标准化问卷。如果村医的测试结果未达到培训标准，研究组织者会为村医安排额外培训。县级心血管专家需要每年为县下属受训的村医提供2次的现场技术指导，并且，如果已接受培训的村医辞职或离岗，县级心血管专家将有责任为新上任的村医提供相应的干预方案培训。这种“手把手”对村医的培训方式（或称为培训师培训）为干预方案的成功开展提出了最佳方案。

- **医疗行为反馈**

本研究将会在当地招募一名全职的研究监查员，该监查员需要熟悉当地文化背景与精通当地方言。监查员将会每两个月对参加干预的村级卫生诊所进行访视。在访视过程中，监查员将使用笔记本电脑收集卫生诊所的临床管理记录。所有信息将会保存到由北京大学医学部临床研究所管理的网络中心数据库中。该中心数据库将会根据收集的信息，为负责各村卫生所的村医计算关键医疗行为指标，这些指标将基于最终版本的技术培训手册。

提供给村医的医疗行为评估报告中将包括：村医所达到的医疗行为指标，该村卫生诊所所属县的平均医疗行为水平以及作为基准的最好的10% 的医疗行为水平。每隔6个月，各村的研究监查员或县级医生/公共卫生研究员将会给各村医提供过去半年的医疗行为评估报告。在其余的时间里，任何医疗行为评估中发现的问题将会由该县级心血管专家（培训导师）或县级临床医师与发生问题的村医进行及时沟通。此定期与非定期的及时医疗行为评估报告机制是本干预方案的一个重要组成部分。通过此机制，能够让村医更好地理解与执行干预方案中的培训内容；并且经济激励的分配方案也是通过此机制制定的。

- **经济激励**

当村医的医疗行为评估指标达到干预方案中规定的标准时，研究组织方将会按照村医的医疗行为评估报告在回馈年度医疗行为评估报告的同时给予经济激励。经济激励的上限被定为每成功管理一位病人，每年将得到10元人民币的激励。经济激励每年支付一次（约在完成首次培训后的第9和第21个月）。如果情况允许，我们将会请求县级医师代劳将医疗行为评估报告以及相应的经济激励交给各村村医。

由于近期中国正在推行医疗体制改革，政府非常重视中国农村地区的健康问题，因此，国家专门划拨了针对改善农村地区卫生健康的经费。我们将会尝试联系县级政府有关部门，商讨其是否同意向中央政府申请专门解决农村地区慢性病管理的专项经费，经费将会用于此PCP干预的经济激励。如果成功申请到经费，将会严格按照经济激励分配的方案分配给参加干预的村落，而未参与干预的同县村落将不会收到任何此项经费的资助。县级政府通过为该PCP干预项目提供由政府设立的专门针对农村地区慢性病管理的经费，能够更好、更有效、更科学地运用国家资源改善农村地区人民的健康水平。如果县级政府不同意使用由国家提供的经费，所有在干预组中村医的经济激励将会由本研究经费支付。所有参加干预组村医的经费将会按照各自业绩考核水平，由县级政府或本研究组织支付。

## 附件2：心脑血管病高危人群病例管理记录表

**心脑血管病高危人群病例管理记录表**

填表说明：

1. 本表仅供课题研究评估使用，版权所有，不得挪作他用！
2. 本表由“技术方案”干预组的村医填写，请每名村医根据就诊患者的真实情况如实填写。
3. 在填写选择题时，请在每一所选答案前的空格“□”中打“√”，表示选择该项答案，不符合的答案不需打“√”。例如在填写问题“性别：□男性 □女性”时，如患者为男性，则在男性前的□中打“√”，即“性别：男性 □女性”。
4. 在标注“可多选”的问题中，如患者的回答或状况同时符合多个答案，则可在这些答案前面的空格□中均打上“√”，不符合的答案不需打“√”，如无任何符合的答案则跳过该题。未标注“可多选”的问题均为单选。
5. 在填写填空题时，请在横线“ ”处填写患者的真实信息。谢谢合作！

**患者首诊记录表**

| 1. **一般资料** | |
| --- | --- |
| - 1. 姓名： | |
| - 1. 性别： □男性 □女性 | |
| - 1. 出生日期： 年 月 （如出生日期不详，请填实足年龄 岁） | |
| - 1. 文化程度：□未上学 □小学 □初中 □高中及以上 | |
| - 1. 家庭住址： 联系电话： | |
| 1. **现病史** | |
| - 1. 主诉： | |
|  | |
|  | |
| - 1. 有无以下症状（可多选）： | |
| □剧烈头痛 □视物模糊 □剧烈呕吐 □意识不清 □胸痛 | |
| □心慌 □气短 □不能平卧 □尿量减少 □肢体麻木或活动障碍 | |
| - 1. 其他症状： | |
|  | |
|  | |
| 1. **心脑血管基础疾病及个人史** | |
| - 1. 基础疾病：您是否曾由县级及以上医院诊断为下列疾病？**（可多选）** | |
| □高血压 □冠心病 □出血性脑卒中 □缺血性脑卒中 □糖尿病 | |
| - 1. 个人用药史：在过去的一个月内您是否规律服用以下药物？**（可多选）** | |
| □降压药 □阿司匹林 □他汀类 □降血糖药 | |
| - 1. 吸烟史：您现在吸烟吗？ □吸烟 □不吸烟**（转至问题3.4）** | |
| 如果您现在吸烟，请问您平均每天吸多少支烟： 支/天 | |
| - 1. 饮酒史：您现在是否有大量饮酒的嗜好？ □有 □没有**（转至问题3.5）** | |
| 如果有，请问您平均每周有多少天会大量饮酒： 天 | |
| **（备注：“大量饮酒”指的是每次饮白酒量超过3两）** | |
| - 1. 您家平均每个月需要用掉多少包食盐： 包 | |
| 每包食盐有多少斤： 斤，您家一般有几口人经常在家吃饭： 人 | |
| - 1. 您目前经常锻炼身体吗？（如农活、散步、做操、爬山等） | |
| □几乎每天都锻炼 □每周3-5次 □每周1-2次 □基本不锻炼 | |
| 1. **体格检查** | |
| - 1. 身高： cm 体重： kg | |
| - 1. 血压水平（收缩压/舒张压）： | |
| 第一次测量： / mmHg | |
| 第二次测量： / mmHg | |
| - 1. 其他检查： | |
|  | |
|  | |
| 1. **药物治疗措施** | |
| - 1. 本次就诊是否开出以下药物处方**（可多选）**： | |
| □降压药 | 如有，请填写商品名： ，和剂量： |
| □阿司匹林 | 如有，请填写商品名： ，和剂量： |
| □他汀类 | 如有，请填写商品名： ，和剂量： |
| □降血糖药 | 如有，请填写商品名： ，和剂量： |
| - 1. 其他药物治疗措施： | |
|  | |
|  | |
| 1. **生活饮食习惯建议** | |
| - 1. 本次就诊是否有以下建议**（可多选）**： | |
| □戒烟 □限盐 □降低体重 □避免大量饮酒 □每天锻炼 | |
| - 1. 其他建议： | |
|  | |
|  | |
|  | |
| 首诊日期： 年 月 日 | |
| 村医签名： | |

**第** 次随访表

| 1. 体重： kg | |
| --- | --- |
| 1. 血压水平（收缩压/舒张压） 第一次测量： / mmHg | |
| 第二次测量： / mmHg | |
| 1. 从上次就诊到现在，您是否由县级及以上医院诊断为以下疾病？**（可多选）** | |
| □冠心病 | 如是，请详细填写发病日期： 年 月 日 |
| □出血性脑卒中 | 如是，请详细填写发病日期： 年 月 日 |
| □缺血性脑卒中 | 如是，请详细填写发病日期： 年 月 日 |
| 1. 本次就诊，需要中止使用的药物有**（可多选）**： | |
| □降压药 □阿司匹林 □他汀类 □降血糖药 | |
| 停用原因： | |
|  | |
| 1. 本次就诊，新开出的药物有（可多选）： | |
| □降压药 | 如有，请填写商品名： ，和剂量： |
| □阿司匹林 | 如有，请填写商品名： ，和剂量： |
| □他汀类 | 如有，请填写商品名： ，和剂量： |
| □降血糖药 | 如有，请填写商品名： ，和剂量： |
| 备注: | |
|  | |
| 本次随访日期： 年 月 日 | |
| 村医签名： | |

**第** 次随访表

| 1. 体重： kg | |
| --- | --- |
| 1. 血压水平（收缩压/舒张压） 第一次测量： / mmHg | |
| 第二次测量： / mmHg | |
| 1. 从上次就诊到现在，您是否由县级及以上医院诊断为以下疾病？**（可多选）** | |
| □冠心病 | 如是，请详细填写发病日期： 年 月 日 |
| □出血性脑卒中 | 如是，请详细填写发病日期： 年 月 日 |
| □缺血性脑卒中 | 如是，请详细填写发病日期： 年 月 日 |
| 1. 本次就诊，需要中止使用的药物有**（可多选）**： | |
| □降压药 □阿司匹林 □他汀类 □降血糖药 | |
| 停用原因： | |
|  | |
| 1. 本次就诊，新开出的药物有（可多选）： | |
| □降压药 | 如有，请填写商品名： ，和剂量： |
| □阿司匹林 | 如有，请填写商品名： ，和剂量： |
| □他汀类 | 如有，请填写商品名： ，和剂量： |
| □降血糖药 | 如有，请填写商品名： ，和剂量： |
| 备注: | |
|  | |
| 本次随访日期： 年 月 日 | |
| 村医签名： | |

## 附件3：效果评价（基线/复查）调查表

**效果评价（基线/复查）调查表**

填表说明：

1. 本表仅供课题研究评估使用，版权所有，不得挪作他用！
2. 横断面调查将在本研究的干预开始前和干预结束后分别进行一次，本表由项目调查员填写，请每名调查员根据受访者的真实情况如实填写。
3. 在填写选择题时，请在每一所选答案前的空格“□”中打“√”，表示选择该项答案，不符合的答案不需打“√”。例如在填写问题“性别：□男性 □女性”时，如患者为男性，则在男性前的□中打“√”，即“性别：男性 □女性”。
4. 在标注“可多选”的问题中，如患者的回答或状况同时符合多个答案，则可在这些答案前面的空格□中均打上“√”，不符合的答案不需打“√”，如无任何符合的答案则跳过该题。未标注“可多选”的问题均为单选。
5. 在填写填空题时，请在横线“ ”处填写患者的真实信息。谢谢合作！

| - 1. **基本信息** | | |
| --- | --- | --- |
| - 1. 姓名： | | |
| - 1. 性别： □男性 □女性 | | |
| - 1. 出生日期： 年 月 （如出生日期不详，请填实足年龄 岁） | | |
| - 1. 文化程度：□未上学 □小学 □初中 □高中及以上 | | |
| - 1. **心脑血管基础疾病史** | | |
| - 1. 基础疾病：您是否曾由县级及以上医院诊断为下列疾病？**（可多选）** | | |
| □冠心病 □出血性脑卒中 □缺血性脑卒中 □糖尿病 | | |
| - 1. 请问您在50岁（男）/60岁（女）之后是否测量过血压？ □是 □否**（转至问题3.1）** | | |
| 如选是，请问您在50岁（男）/60岁（女）之后，收缩压的最高值为 mmHg | | |
| - 1. **体格检查** | | |
| - 1. 身高： cm 体重： kg | | |
| - 1. 血压水平（收缩压/舒张压）： | | - 1. 心率： |
| 第一次测量： / mmHg | | 第一次测量： 次/分钟 |
| 第二次测量： / mmHg | | 第二次测量： 次/分钟 |
| - 1. **生活饮食习惯** | | |
| - 1. 吸烟史：您现在吸烟吗？ □吸烟 □不吸烟**（转至问题4.2）** | | |
| 如果您现在吸烟，请问您平均每天吸多少支烟： 支/天 | | |
| - 1. 饮酒史：您现在是否有大量饮酒的嗜好？ □有 □没有**（转至问题4.3）** | | |
| 如果有，请问您平均每周有多少天会大量饮酒： 天 | | |
| **（备注：“大量饮酒”指的是每次饮白酒量超过3两）** | | |
| - 1. 您家平均每个月需要用掉多少包食盐： 包 | | |
| 每包食盐有多少斤： 斤，您家一般有几口人经常在家吃饭： 人 | | |
| - 1. 您目前经常锻炼身体吗？（如农活、散步、做操、爬山等） | | |
| □几乎每天都锻炼 □每周3-5次 □每周1-2次 □基本不锻炼 | | |
| - 1. **诊疗情况** | | |
| - 1. 在过去的12个月中，您是否因心脑血管疾病到村医处就诊？ | | |
| □是 □否**（转至问题5.2）** | | |
| 如果选是，请问过去12个月中，您总共去过的次数为： 次 | | |
| - 1. 在您每次就诊时，村医是否给您测量血压？ □是 □否 | | |
| - 1. 在过去的12个月中，您是否服用药物？ □是 □否**（转至问题5.4）** | | |
| 如选是，是否服用以下药物？**（可多选）** | | |
| □降压药 | 如有服用，请问该药是否由村医所开？ □是 □否 | |
| □阿司匹林 | 如有服用，请问该药是否由村医所开？ □是 □否 | |
| □他汀类 | 如有服用，请问该药是否由村医所开？ □是 □否 | |
| □降血糖药 | 如有服用，请问该药是否由村医所开？ □是 □否 | |
| - 1. 在过去的12个月中，您是否从村医处获得以下建议？**（可多选）** | | |
| □减少食盐摄入 □戒烟 □避免大量饮酒 □降低体重 □加强锻炼 | | |
| - 1. 您对自己目前疾病的治疗及控制状况是否满意？ □是 □否 | | |
| 调查日期： 年 月 日 | | |
| 调查员签名： | | |

## 附件4：中国农村健康行动以村医为基础的心脑血管病防治干预计划知情同意书

（一式两份。调查员保留一份，被调查者保留一份）

**中国农村健康行动以村医为基础的心脑血管病防治干预计划知情同意书**

尊敬的先生/女士：

“中国农村健康行动” 正在你所在的地区开展一项由村医执行的“心脑血管病高危人群规范化管理”计划。为了科学评价此计划的实施效果，将有1/2的乡村被随即分配到干预组，另1/2的乡村则被分配到对照组。干预组的村医将要接受由项目提供的统一技术培训，并要按照项目要求检出、分类、治疗和随访管理所服务村庄的心血管病高危人群。对照组的医生则继续按照自己的平常诊疗习惯行医。

当您阅读这份知情同意书时，您所在的村已经被上级卫生行政部门选定作为干预村/对照村。接下来，我们邀请您加入“心脑血管病高危人群规范化管理”干预计划。在您决定是否参加之前，请仔细阅读以下内容。

**干预组村医需要做的事情**

1）接受技术培训

干预组的村医将接受由项目指定的的心血管专家提供的“基于村医的心血管病规范化管理方案”技术培训，为期2天。培训内容主要包括：心血管高危人群的有效检出方法；廉价、简单、有效的心血管病风险控制方案；以及如何正确记录病人的临床管理资料。培训结束时，您需要完成一份针对培训内容设计的测试问卷。如果测试结果未达预期标准，研究组织方会为您安排额外培训。

2）按照项目提供的技术方案，开展心血管病高危人群规范化管理

通过培训考核后，村医需按照项目所提供的“基于村医的心脑血管病规范化管理方案”，通过自己的日常医疗工作，筛查确认未来10年发生心血管病机会较大的高危人群，为患者建立“心脑血管病高危人群规范化管理病历记录册”，开展定期的科学管理和治疗，以期达到有效预防心血管病发生的目的。

3）接受监查

在项目开展期间，项目监察员将会定期到您所在的诊所查看“心血管病高危人群病历管理记录册”，收集并上传在册患者的临床管理记录；每六个月您将会收到临床管理质量与效果的评估反馈。

4）配合进行相关调查，以科学评价干预效果

干预计划实施前后，我们将在您所在的乡村开展小规模的抽样调查，以评价干预的效果。届时需要您的大力配合，以便及时找到并召集需调查人员。

**对照组村医需要做的事情**

1）按照自己的平常诊疗习惯行医

在对照组的村医，项目不会对其进行疾病诊疗方面的任何特别要求和干预。村以可根据自己对于各种疾病的认识和所掌握的知识和技能决定自己的诊疗行为。

4）配合进行相关调查，以科学评价干预效果

干预计划实施前后，我们将在您所在的乡村开展小规模的抽样调查，以了解本村村民心脑血管病的防治状况和自然变迁。届时需要您的大力配合，以便及时找到并召集需调查人员。

**信息保密**

上述“心脑血管病高危人群规范化管理（电子）病历”中所有调查资料将加密保存在北京大学临床研究所的中心数据库内，得到严密保管，并且只有您和得到授权的研究人员可以查阅。这些资料仅用于学术研究，绝对不会以任何形式公布或泄漏给第三方。

**调查的组织者和实施者**

此项目是由中国国际慢性病预防中心与北京大学医学部、中国医科大学、西安交通大学医学院、河北省疾病控制预防中心、宁夏医科大学和山西省长治医学院共同组织和实施。此项目得到了国家卫生部和所在各省市、自治区卫生厅的支持。

**利益与风险**

干预组的村医将得到一次难得的技术培训，其开展心脑血管病防治的技能将得到明显提高。根据您所管理患者的数量和临床控制效果，我们将对您的努力给予适当的经济激励。对照组的村医因不实施干预计划，不能得到相应的培训和经济激励。

干预组的村医需要为项目实施投入额外的时间和精力，需要脱产2天参加技术培训，因此也可能影响自己的业务收入。

此项计划的干预效果一经证实，不仅干预组的患者会直接获益，而且项目推广后将使我国千千万万的农民最终获益。无论干预组还是对照组的村医，对这项科学研究的成果都有相同的贡献。

**自愿参加**

您的参加对于从科学上明确上述干预方案的防治效果有重要价值，但是否参加此项研究完全依从您的个人意愿。如果有需要，您可以与朋友或亲人讨论。您也可以在任何时间要求退出研究。

**联系方式**

如果有与此项研究有关的问题，您可以直接与在北京的项目协调员郝志新女士联系。

具体联系方式为：

电话: 010-8280-0577 转 310

传真: 010 8280-0177

电子邮件: [jhao@george.org.cn](mailto:jhao@george.org.cn)

地址: 北京市海淀区知春路6号锦秋国际大厦B1302室

或者您可以致电您所在地的项目协调员。联系方式分别是：.

辽宁 024-83282632，河北 0311-86573288，山西 0355-3033238，

陕西 029-82655107，宁夏 0951-6980144。

**~~~ 十分感谢您的合作 ~~~**

**村医信息：**:

姓名

地址

个人编号

- 我自愿同意参加此研究项目。
- 我已经询问与此研究项目有关的问题，并且所有问题都已得到满意的回答。
- 我完全明白此研究项目的意义、目的、持续时间、程序以及由于参与此项目可能带来的不便。
- 我已经完整阅读过此研究项目的相关资料，并且同意参与此研究项目。我明白我可以选择在任何时候通知研究者要求退出研究。我已经收到此知情同意书的副本。

**村医签名：____________________ 日期：____________________**

**监察员签名：____________________ 日期：____________________**

## 附件5：心血管高危患者病例记录管理知情同意书

（一式两份。调查员保留一份，被调查者保留一份）

**中国农村健康行动心血管高危患者病例记录管理知情同意书**

尊敬的先生/女士：

“中国农村健康行动”正在你所居住的乡村开展一项由村医执行的心脑血管疾病防治计划。此计划的一项重要干预手段是筛查确认未来10年发生心血管病机会较大的高危人群，并开展定期的科学管理和治疗，以期达到有效预防心血管病发生的目的。当您阅读这份知情同意书时，您已经被村医诊断并确认属于未来容易发生心脑血管病的高危人群，这包括：

1）曾经得过明确的冠心病和脑卒中的人；

2）年龄大于50岁的男人和年龄大于60岁的女人，且具有以下任意条件：

- 糖尿病
- 收缩压>=160mmHg

接下来，我们邀请您加入“心脑血管病高危人群规范化管理”干预计划。在您决定是否参加之前，请仔细阅读以下内容。

**您需要做的事情**

如果您同意参加此干预计划，村医将按照我们统一制定的标准化技术方案对您开展定期的检查和治疗，以及健康生活方式指导等。您需要定期回到诊所（或由村医定期上门）进行血压测量及了解预防治疗效果和最新病情。同时，我们将为您建立一份专门的“心脑血管病高危人群规范化管理（电子）病历”，记录您的个人基本信息、生活方式/习惯、疾病史、家族史、药物使用情况、最新症状体征、血压、心率、体重等。这份病历将有效地帮助医生了解您的病情长期变化和治疗效果。

**信息保密**

上述“心脑血管病高危人群规范化管理（电子）病历”中所有调查资料将加密保存在北京大学临床研究所的中心数据库内，得到严密保管，并且只有您的医生和得到授权的研究人员可以查阅。这些资料仅用于学术研究，绝对不会以任何形式公布或泄漏给第三方。

**调查的组织者和实施者**

此项目是由中国国际慢性病预防中心与北京大学医学部、中国医科大学、西安交通大学医学院、河北省疾病控制预防中心、宁夏医科大学和山西省长治医学院共同组织和实施。此项目得到了国家卫生部和所在各省市、自治区卫生厅的支持。

**利益与风险**

参加此项干预计划将使您得到及时、有效、科学的医学照顾，您未来发生心脑血管病的机会也会因此大大降低。此项干预方案不涉及任何有创性检查或治疗，但将包括一些必要的药物治疗。这些药物的使用都依据了当前国内外的相关指南。总的来说，发生药物不良反应的机会不大，但并不能完全排除。

**自愿参加**

您的参加对于从科学上明确上述干预方案的防治效果有重要价值，但是否参加此项研究完全依从您的个人意愿。如果有需要，您可以与朋友或亲人讨论。您也可以在任何时间要求退出研究。

**联系方式**

如果有与此项研究有关的问题，您可以直接与在北京的项目协调员郝志新女士联系。

具体联系方式为：

电话: 010-8280-0577 转 310

传真: 010 8280-0177

电子邮件: [jhao@george.org.cn](mailto:jhao@george.org.cn)

地址: 北京市海淀区知春路6号锦秋国际大厦B1302室

或者您可以致电您所在地的项目协调员。联系方式分别是：.

辽宁 024-83282632，河北 0311-86573288，山西 0355-3033238，

陕西 029-82655107，宁夏 0951-6980144。

**~~~ 十分感谢您的合作 ~~~**

**被管理者**:

姓名

地址

个人编号

- 我自愿同意参加此研究项目。
- 我已经询问与此研究项目有关的问题，并且所有问题都已得到满意的回答。
- 我完全明白此研究项目的意义、目的、持续时间、程序以及由于参与此项目可能带来的不便。
- 我已经完整阅读过此研究项目的相关资料，并且同意参与此研究项目。我明白我可以选择在任何时候通知研究者要求退出研究。我已经收到此知情同意书的副本。

**患者签名：____________________ 日期：____________________**

**村医签名：____________________ 日期：____________________**

## 附件6：效果评价（基线/复查）被访对象知情同意书

（一式两份。调查员保留一份，被调查者保留一份）

**中国农村健康行动效果评价(基线/复查)被访对象知情同意书**

尊敬的先生/女士：

“中国农村健康行动” **计划/已经** 在你所居住的乡村开展一项由村医执行的心脑血管疾病防治计划。为了准确、客观地评价此干预计划的真实效果，我们需要对您以及村里的其他一些居民做一些调查。在您决定是否参加此调查之前，请仔细阅读以下内容。

**您需要做的事情**

如果您同意参加此调查，您需要对调查员的一些问题做出回答。这些问题包括个人基本信息、生活方式/习惯、疾病史、药物使用情况、求诊习惯；同时我们会为您测量血压、心率、身高以及体重。全过程大约10-30分钟。

**信息保密**

上述所有调查资料将加密保存在北京大学临床研究所的中心数据库内，得到严密保管，并且只有得到授权的研究人员可以查阅。这些资料仅用于学术研究，绝对不会以任何形式公布或泄漏给第三方。

**调查的组织者和实施者**

此调查是由中国国际慢性病预防中心与北京大学医学部、中国医科大学、西安交通大学医学院、河北省疾病控制预防中心、宁夏医科大学和山西省长治医学院共同组织和实施。此项目得到了国家卫生部和所在各省市、自治区卫生厅的支持。

**利益与风险**

为了补偿您为此调查所花费的时间，我们将在调查结束后赠送您一件小礼品。此项调查不涉及任何有创性检查，也不询问任何与健康无关的个人隐私，不会造成对您的任何身体或心理的伤害。

**自愿参加**

是否参加此项研究完全依从您的个人意愿。如果有需要，您可以与朋友或亲人讨论。您也可以在任何时间要求退出研究。

**联系方式**

如果有与此项研究有关的问题，您可以直接与在北京的项目协调员郝志新女士联系。

具体联系方式为：

电话: 010-8280-0577 转 310

传真: 010 8280-0177

电子邮件: [jhao@george.org.cn](mailto:jhao@george.org.cn)

地址: 北京市海淀区知春路6号锦秋国际大厦B1302室

或者您可以致电您所在地的项目协调员。联系方式分别是：.

辽宁 024-83282632，河北 0311-86573288，山西 0355-3033238，

陕西 029-82655107，宁夏 0951-6980144。

**~~~ 十分感谢您的合作 ~~~**

**被调查者**:

姓名

地址

个人编号

- 我自愿同意参加此研究项目。
- 我已经询问与此研究项目有关的问题，并且所有问题都已得到满意的回答。
- 我完全明白此研究项目的意义、目的、持续时间、程序以及由于参与此项目可能带来的不便。
- 我已经完整阅读过此研究项目的相关资料，并且同意参与此研究项目。我明白我可以选择在任何时候通知研究者要求退出研究。我已经收到此知情同意书的副本。

**被调查者签名：____________________ 日期：____________________**

**调查员签名：_________________ ___ 日期：____________________**
