# Supplementary material for: The Effects of a Community-Based Sodium Reduction Program in Rural China – A Cluster-Randomized Trial
Source: PLoS One. 2016 Dec 9;11(12):e0166620. doi: 10.1371/journal.pone.0166620 (PMC5147834; doi:10.1371/journal.pone.0166620)
Supplement: S4 File — (DOC) [file pone.0166620.s006.doc]

**China Rural Health Initiative**

**High cardiovascular risk management and salt reduction in rural villages in China: a factorial cluster-randomized controlled trial**

**PROTOCOL**

TABLE OF CONTENTS

[OVERALL GOAL, SPECIFIC AIMS AND HYPOTHESES 4](#__RefHeading___Toc271588133)

[BACKGROUND AND SIGNIFICANCE 5](#__RefHeading___Toc271588134)

[BURDEN OF CARDIOVASCULAR DISEASE IN RURAL CHINA 5](#__RefHeading___Toc271588135)

[THE CHINESE RURAL POPULATION AND HEALTHCARE SYSTEM 5](#__RefHeading___Toc271588136)

[THE CONTROL OF BLOOD PRESSURE IN CHINA 6](#__RefHeading___Toc271588137)

[CURRENT MANAGEMENT OF CARDIOVASCULAR DISEASE IN RURAL CHINA 7](#__RefHeading___Toc271588138)

[DRUG-BASED INTERVENTIONS FOR STROKE PREVENTION 7](#__RefHeading___Toc271588139)

[SALT REDUCTION FOR STROKE PREVENTION 8](#__RefHeading___Toc271588140)

[THE EFFECTS OF PRIMARY CARE PROVIDER TRAINING 8](#__RefHeading___Toc271588141)

[PILOT WORK UNDER-PINNING THIS PROJECT 8](#__RefHeading___Toc271588142)

[A Simplified Approach to Managing Cardiovascular Disease in Chinese Communities 8](#__RefHeading___Toc271588143)

[Development, promotion, and evaluation of “Practical guidelines for community-based hypertension prevention and control” 9](#__RefHeading___Toc271588144)

[The feasibility of implementing a salt reduction program 10](#__RefHeading___Toc271588145)

[SIGNIFICANCE OF THE PROPOSED STUDY 12](#__RefHeading___Toc271588146)

[STUDY DESIGN 13](#__RefHeading___Toc271588147)

[PROVINCES, TOWNSHIPS AND VILLAGES TO BE INCLUDED 13](#__RefHeading___Toc271588148)

[RANDOMIZATION 14](#__RefHeading___Toc271588149)

[INTERVENTION AND CONTROL 14](#__RefHeading___Toc271588150)

[‘Package’ intervention 14](#__RefHeading___Toc271588151)

[‘Package’ control 15](#__RefHeading___Toc271588152)

[‘Salt reduction’ intervention 16](#__RefHeading___Toc271588153)

[‘Salt reduction’ control 17](#__RefHeading___Toc271588154)

[OUTCOMES 17](#__RefHeading___Toc271588155)

[The primary outcome for the package intervention 17](#__RefHeading___Toc271588156)

[The primary outcome for the salt reduction intervention 17](#__RefHeading___Toc271588157)

[Secondary outcomes for the package intervention 17](#__RefHeading___Toc271588158)

[Secondary outcomes for the salt reduction intervention 17](#__RefHeading___Toc271588159)

[Exploratory outcomes 18](#__RefHeading___Toc271588160)

[OUTCOMES EVALUATION 18](#__RefHeading___Toc271588161)

[Baseline and follow-up surveys 18](#__RefHeading___Toc271588162)

[24-hour urinary sodium 19](#__RefHeading___Toc271588163)

[Mortality and morbidity surveillance system 19](#__RefHeading___Toc271588164)

[Case management records 19](#__RefHeading___Toc271588165)

[Village doctor survey 20](#__RefHeading___Toc271588166)

[PROCESS EVALUATION 20](#__RefHeading___Toc271588167)

[ECONOMIC EVALUATION 20](#__RefHeading___Toc271588168)

[STATISTICAL POWER 21](#__RefHeading___Toc271588169)

[ANALYSIS PLAN 22](#__RefHeading___Toc271588170)

[DATA MONITORING AND SAFETY 23](#__RefHeading___Toc271588171)

[PROJECT TIMELINE 23](#__RefHeading___Toc271588172)

[ETHICS REVIEW AND HUMAN SUBJECT PROTECTION 24](#__RefHeading___Toc271588173)

[Human subject protection certification and training 24](#__RefHeading___Toc271588174)

[Participant informed consent 24](#__RefHeading___Toc271588175)

[Participant risks and benefits 25](#__RefHeading___Toc271588176)

[Inclusion of women, minorities, and children 25](#__RefHeading___Toc271588177)

[Amendments and additional submissions 26](#__RefHeading___Toc271588178)

[REFERENCES 27](#__RefHeading___Toc271588179)

[APPENDICES 31](#__RefHeading___Toc271588180)

[APPENDIX 1 – Detailed Description of the CVD Prevention and Management ‘Package’ 32](#__RefHeading___Toc271588181)

[Appendix 1-A: Flow Chart to Identify, Manage, and Follow-up High-Risk Patients 36](#__RefHeading___Toc271588182)

[Appendix 1-C: Key Performance Indicators and Payment Schemes 41](#__RefHeading___Toc271588183)

[APPENDIX 2 – Questionnaire for baseline and follow-up population surveys 43](#__RefHeading___Toc271588184)

[APPENDIX 3 – Questionnaire for village doctors 47](#__RefHeading___Toc271588185)

[APPENDIX 4-A – Participant Information Sheet and Consent Form to be used for village doctors 48](#__RefHeading___Toc271588186)

[APPENDIX 4-B – Participant Information Sheet and Consent Form to be used at time of baseline and follow-up population surveys and interviews 53](#__RefHeading___Toc271588187)

# OVERALL GOAL, SPECIFIC AIMS AND HYPOTHESES

The **overall goal** of this study is to develop, implement and evaluate effective, low-cost, and sustainable interventions for cardiovascular disease prevention and management suitable for widespread implementation in rural China.

The **specific aims** are to evaluate the effects of:

1. Implementing a simple, low cost high cardiovascular risk management package (“package” hereafter) delivered by primary care providers (village doctors), on mean blood pressure of individuals at high risk of cardiovascular disease (“high-risk individuals” hereafter).
2. Implementing a community-based salt reduction and health promotion program delivered by community health educators on mean blood pressure of high-risk individuals (and other adults).

The corresponding **null** **hypotheses** that will be tested are:

1. Implementing the package will have not have “significant” impact upon mean blood pressure of high-risk individuals.
2. Implementing the community-based salt reduction program will not have “significant” impact upon mean blood pressure of high-risk individuals and other adults.

It is worth noting that the null hypotheses are specified in a non-typical way to include “significant” impact in them. The rationale is that for this project with strong governmental support from its inception and potentially large policy implication if proven effective, we expect a larger than usual effect size for the results to be meaningful to policy makers and for widespread adoption in rural China. If the effect size is only scientifically, statistically or even clinically meaningful, it won’t be considered “effective” or an impact “significant” enough to support national policy decision making.

# BACKGROUND AND SIGNIFICANCE

Cardiovascular disease is the leading cause of death in both developed and developing regions and the great majority of cardiovascular disease occurs in low and middle income countries.[1] In addition, about half of the deaths attributable to cardiovascular disease in developing countries occur below the age of 70 years, in contrast to about a quarter in developed countries. With no end to the rise in cardiovascular disease burden in developing counties in sight,[2] the identification of novel strategies that can address cardiovascular disease burden in developing countries is a global health priority.[3-4]

## BURDEN OF CARDIOVASCULAR DISEASE IN RURAL CHINA

Cardiovascular disease is the leading cause of death in China, responsible for about 3.5 million (38%) deaths in 2008.[1] By contrast to Western countries, stroke is much more prevalent and a greater cause of disease burden than coronary heart disease. There is also marked variation in the pattern of cardiovascular disease within China, with higher rates in Northern regions and lower rates in Southern regions.[5-6] Reflecting the major contribution of cerebrovascular disease to the vascular disease burden in China, high blood pressure is the leading modifiable risk factor for cardiovascular disease[7] and its importance is greatest in rural and northern regions where salt consumption, high blood pressure and the incidence of stroke are both very high.

## THE CHINESE RURAL POPULATION AND HEALTHCARE SYSTEM

The majority of the 1.2 billion Chinese population live in rural areas.[8] China has 33 provincial administrations including 283 cities and 2859 rural counties.[9] Each county is further divided into about 15 townships (average township population size = 20000) and each township, in turn, comprises between 10 and 20 villages (average village population size = 1500). The rural healthcare system has a 3-tiered structure that mirrors the broader administrative organization of rural China - at the county level there are county hospitals and county centers for disease prevention and control, at the township level there are township community healthcare centers, and finally beneath that the village clinics (or healthcare stations).[8] The village clinics are administered and receive technical support from the townships and the townships are, in turn, administered and supported by the county Bureau of Health, comprised of the county hospital and county centers for disease control. On average in rural China there are 1.2 health care workers per 1000 residents, with the village primary care providers comprising a substantial proportion.

In each village, there is usually at least one primary care provider, a licensed village doctor who typically has received a limited professional training after completing high school. Most of the village doctors have prescription rights for basic medicines on the national essential drug list. The village clinics, themselves, are typically one or two dedicated rooms although most will contain only very limited medical equipment. In some villages, the village doctor is assisted by another healthcare worker, usually a woman, who has responsibility for maternal and child health and health education. The primary care providers and other healthcare workers live in the villages and work part-time with a modest salary provided by the county government; many work primarily in private practice although in some developed rural areas they may be full-time employees of the township government. Most consultations and subsequent care will require additional out-of-pocket payments by the patient to the primary care provider.

## THE CONTROL OF BLOOD PRESSURE IN CHINA

In 2002, the prevalence of hypertension in China was broadly similar in rural (17%) and urban areas (21%),[10] although awareness, treatment, and control rates were significantly lower outside of the cities (19%, 13%, and 3% vs. 30%, 24%, and 6% respectively).[10] The gaps between these figures and those achieved in developed countries such as the US (66.5%, 53.7%, and 33.1% respectively in 2003-2004),[11] serve to demonstrate both the lack of adequate intervention and the great potential for improvement in China. There is clear evidence that, for most individuals, blood pressure-related risk can be effectively managed by using low-cost drug treatments.[12] Adopting a healthy lifestyle can also help individuals to control their blood pressure[13] although adherence to the required dietary and behavioral changes is often poor and the real potential for these strategies lies in their population-wide application through centralized approaches to intervention.[14]

China has established guidelines for the control of blood pressure that provide comprehensive guidance on diagnosis, management and monitoring.[15] However, as is the situation for most countries, these guidelines are implemented incompletely in China and there is a need for the key messages to be disseminated more widely and adapted to different settings for not only better understanding of the guidelines but also better achievements of treatment goals in clinical practices especially in the primary care settings. Simplification and abbreviation is particularly important if blood pressure control is to be improved in rural areas where the primary care providers have only minimal training and limited access to resources.

Another reason for the review of existing hypertension guidelines is the need to ensure that the scarce resources available to rural areas are assigned as efficiently as possible. Accordingly, most guidelines for the prevention of blood pressure-related diseases in developed countries now incorporate absolute risk assessment as a key component of the process for deciding which individuals should be treated.[16-18] The rationale for this approach is that the association between blood pressure and vascular risk is continuous, not dichotomous, and the observation that blood pressure alone (unless it is very high) is a rather poor discriminator of risk. A series of studies have shown the much greater efficiency of allocating blood pressure lowering on the basis of an absolute risk assessment rather than a blood pressure measurement alone.[19-20] This approach means that fewer patients can be treated, at lower cost, with more events averted because lower risk patients with borderline hypertension are not treated while high risk patients with ‘normal’ blood pressure are treated.

Since the Framingham risk prediction score does not fit the Chinese population, we used Chinese 10-year fatal and non-fatal ischemic cardiovascular disease risk prediction score to estimate the least risk for our defined high cardiovascular risk individuals.[21] According to these estimations, the 10-year risks are 7.0% for men aged 50-years old and 5.0% for women aged 60 years old who only had SBP≥160 mmHg but had no other risk factors such as smoking, overweight, high cholesterol and diabetes. Considering the high prevalence of those risk factors among the defined high risk population, the estimated 10-year absolute risk of ischemic cardiovascular disease would be at least 8-10% for individuals meeting our high-risk definition: either 1) having a disease history of coronary heart disease, ischemic or hemmorraghic stroke (of any age); or 2) meeting the age criteria (50 or over for men, 60 or over for women) and having SBP > 160 mmHg; or 3) meeting the age criteria (50 or over for men, 60 or over for women) and having diabetes.

## CURRENT MANAGEMENT OF CARDIOVASCULAR DISEASE IN RURAL CHINA

At the village level, strategies for the control of cardiovascular disease are mostly absent. National clinical guidelines for the management of hypertension and cardiovascular disease are rarely disseminated to, or implemented by the village primary care providers. The financial system under which the system operates further mitigates against the uptake of routine preventive screening and care by the community.

Identified priority areas for cardiovascular disease control in rural areas are high blood pressure (driven by very high-salt intakes)[22-26] and smoking (particularly among men).[1, 10] Physical activity levels, by contrast, remain favorable compared to their urban counterparts, with many rural residents engaged in farm work. Another important consideration is that while rural-urban migration has been a dominant feature of recent Chinese demographic changes, most migrant workers that fall ill return to their country homes, where they often require long-term care. The currently very limited capacity of the medical personnel coupled with an increasing prevalence and morbidity caused by cardiovascular disease provides a strong rationale for intervention at both the clinical and population level.

## DRUG-BASED INTERVENTIONS FOR STROKE PREVENTION

Results from large-scale randomized trials have identified a number of highly effective interventions for stroke prevention.[12] Blood pressure lowering regimes and anti-platelet therapy,[27] in particular, are highly effective strategies for prevention amongst high-risk individuals. Furthermore, with the advent of widely available generic medicines, drug-based management can be afforded by the health systems and communities of even developing countries. The wider use of these therapies has great potential to reduce the morbidity, mortality, and economic burdens of cardiovascular disease in rural China.

## SALT REDUCTION FOR STROKE PREVENTION

There is clear evidence from both observational studies[28] and overviews of randomised trials[29],[30] that lower levels of salt consumption are associated with lower levels of blood pressure.[30-31] Similarly clear are the beneficial effects of blood pressure lowering on major vascular events.[32-34] While the data directly linking salt reduction with mortality and morbidity benefits is more limited,[28, 35-39] the totality of evidence relating to the effects of salt on vascular disease is compelling. Most recently, the World Health Organisation has issued a technical report recommending that each of its member states implement a national salt reduction strategy for vascular disease prevention.[40]

Salt reduction has greater potential in rural China than almost anywhere else in the world. Very high levels of salt consumption,[41-42] very little use of processed food and most dietary salt deriving from home cooking makes the removal of salt from the diet easier, cheaper and more worthwhile than in almost any other setting.[20] Interventions that jointly sought to educate the population about the harmful effects of salt and to decrease consumption levels,[43] in conjunction with efforts to make salt substitute[44] more widely available would be expected to have the greatest impact.

## THE EFFECTS OF PRIMARY CARE PROVIDER TRAINING

Previous research on the effects of primary care provider training has been limited. It has been reported that special skills training could help rural physicians upgrade their skills and gain new competencies. In addition, physicians after the initial training are able to pursue more training opportunities on their own in the future and return to practice in their rural communities than in the past.[45] One small study conducted by our research group in rural China demonstrated that the hypertension control rate could be significantly improved when village doctors receive proper training on simplified clinical guidelines tailored to their level and educational background aided by certain management and support to village doctors (see the next section for more details). Some studies investigated both a provider-based approach and a patient-based approach and generally found that the combined approaches are more effective than physician training alone.[4, 46-47] For example, a study conducted in rural Pakistan [46] showed that the multivariable reduction in systolic blood pressure was significantly greater in the combined intervention group (10.8 mmHg, 95% CI: 8.9 to 12.8 mmHg) than in the physician intervention, patient intervention, or control groups (*P*<0.001). These studies indicated that a combined intervention including physician intervention and patient intervention had bigger effects compared with one intervention alone. Therefore, in this trial, both a primary-care based intervention and a community-based intervention targeting high-risk individuals and other rural residents will be evaluated.

## PILOT WORK UNDER-PINNING THIS PROJECT

### A Simplified Approach to Managing Cardiovascular Disease in Chinese Communities

In collaboration with colleagues in Canada we conducted a pilot study to evaluate a simplified approach to managing cardiovascular disease using two community health centers in Beijing (1 urban and 1 rural) and two centers in Baoding City, Hebei Province (1 urban and 1 rural). Both quantitative methods (chart review and questionnaire surveys of 50 patients in each center) and qualitative methods (participatory observation and focus groups with physicians and patients separately) were used. The baseline hypertension control rate was 29.3% in urban centers and 13.9% in rural centers. Use of anti-hypertensive medication (including traditional Chinese medicine) was similarly low in both rural (30.6%) and urban (30.2%) centers as was use of aspirin (36.9% vs. 31.4%). Rates of diuretic use were also very low (6.9% urban and 3.2% rural).

### Development, promotion, and evaluation of “Practical guidelines for community-based hypertension prevention and control”

In 2001, the PI of the present study convened a national 50-member expert committee supported by the Chinese Cardiology Society, Beijing Hypertension Prevention and Control Committee, National Cardiovascular Prevention and Control Institute, WHO cardiovascular research and training collaborative center, and China CDC. On the basis of “Clinical guidelines for hypertension prevention and control in China,” a simplified practical yet evidence-based and validated guideline “The Practical Guidelines for Community-Based Hypertension Prevention and control” was developed by this expert committee through 12 group discussions and the Delphi method. The practical guideline was publicly released in October 2002 in Beijing at the Hypertension and Related Diseases Academic Conference. In 2003, the Bureau of Disease Prevention and Control in the China Ministry of Health approved the promotion of “The Practical Hypertension Prevention and control Guidelines for Grass-root Levels” nationwide. Within a year, over 10 provincial or municipal government agencies including CDC, Bureau of Health, primary care foundation, cardiovascular disease prevention and control office purchased the Guideline for widespread adoption; training classes were held successfully in 7 cities with about 1,800 trainees. The total number of primary care providers trained was over 30,000.

In 2004-5, to evaluate the effect of training village doctors for the practical guidelines, a one-year randomized intervention trial in 2 urban and 2 rural community health centers in Beijing (560 trial patients) showed dramatic improvements in many aspects such as hypertension control rate (26.4% control group vs. 77.9% intervention group in the 2 rural centers; 40.3% vs. 75.2% urban by the end of 12 months) (Table 1). During the intervention period, average pharmaceutical costs of hypertensive patients in urban areas in the intervention group was 569.4 yuan, significantly lower than the control group 766.5 yuan (p<0.05); in rural areas, also lower but not statistically significant (295.6 vs. 354.1, p>0.05).

Table 1 The hypertension control rates at follow-up

Control rateUrbanRuralInterventionControlInterventionControl3 months49.445.835.118.46 months64.350.9*40.323.9*9 months70.452.1*62.022.0**12 months75.240.3**77.926.4**vs. Intervention：* p<0.05; ** p<0.01

###

### The feasibility of implementing a salt reduction program

In 1988, the PI of the present study conducted a small-scale trial to evaluate the feasibility and effect of implementing a salt reduction program in 16 households (38 villagers) in Hanzhong, Shaanxi Province. The results show that, the mean salt intake reduced to 6.1±1.7g/day from 11.1±3.7g/day, after 12-weeks intervention. Average reduction in daily salt intake was 5.0 g, of which 3.9 g/day came from reduced consumption of high-salt food such as preserved vegetables and beans. The urinary sodium and urinary sodium/potassium ratio from 8-hour nightly urinary excretion were all reduced; however, the reductions were not statistically significant. Compared with baseline, the reductions of SBP and DBP were 12.5mmHg and 7.8mmHg (p<0.01 for both). These results demonstrate that simple, clear, and specific dietary instructions on salt reduction can effectively help rural residents reduce their salt intake. Unfortunately, no control group was included in this study.

In 2004-2005 we conducted, in rural China, a randomized trial including 608 high risk individuals recruited from 6 townships (and some 30 villages) in rural and suburban Northern China. Participants were assigned at random to receive either a low-sodium, high potassium salt substitute or continued normal salt. At completion of the 12 months of follow-up systolic blood pressure was 5.4mmHg (95% confidence interval 2.3 to 8.5) lower in the salt substitute group compared to the usual salt group, with evidence that the blood pressure reduction was increasing with time (p=0.001, Figure 1). Adherence to the salt substitute was very high with 98% of participants reporting using the study salt/salt substitute for all or nearly all of their food preparation during the study period.


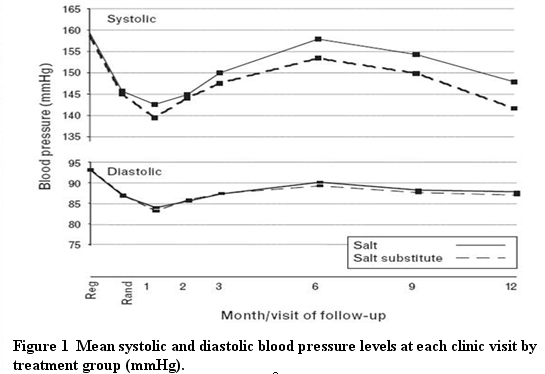


We conducted another randomized double-blind controlled trial on salt substitute among 220 hypertensive patients and their 348 family members in rural Beijing from 2005-2006. After the one-year intervention, systolic blood pressure reduced by 4.1 (0.8, 7.4) mmHg (measured clinically), 3.1 (0.4, 5.8) mmHg (measured by home monitoring), and 2.4 (-2.4, 5.7) mmHg (ambulatory monitoring) respectively. Among family members, systolic BP by home monitoring reduced by 1.5 (-0.4, 3.4) mmHg, of which family members with hypertension had a reduction of 2.7 (-1.9, 7.2) mmHg and with normal blood pressure, only 0.5 (-1.2, 2.1) mmHg. Reduction in systolic BP was bigger with older age. Further analysis showed that withdrawal from medication was more pronounced in the salt substitute group than control group (normal salt), reaching statistical significance by the 3^rd^ month (p<0.05). During the trial, all (100%) people in the salt substitute group reported consuming the salt provided to them “all” or “most” of the time and 98.2% of the participants in the control group (normal salt) reported so. No between-group difference in compliance was detected (p>0.05).

A similar 3-month randomized single-blind trial conducted in Tibet in 2009 among 284 hypertensive patients showed a bigger reduction [-8.8 mmHg (95% CI: -19.6 to -1.0) systolic and -4.5 mmHg (95% CI: -8.5 to -0.6) diastolic respectively] in this high-altitude area with very high prevalence of hypertension and higher average baseline blood pressure level.

There is currently significant interest in the potential for salt substitutes to decrease vascular disease in China and in Beijing there is a directive requiring greatly increased sales of salt substitute by the Beijing Salt Corporation. Against this background there is a high likelihood that it will be possible to make salt substitute more widely available in rural China and this will be a core component of the salt reduction program.

## SIGNIFICANCE OF THE PROPOSED STUDY

Rural China suffers an enormous burden of avoidable death and disability as a consequence of premature vascular disease.[48] There are very well established interventions that could avert much of this burden if the practicalities of how to deliver care to large numbers at low cost can be resolved. Examples of such interventions include blood pressure reduction, salt intake reduction, use of aspirin, and regular follow-up physician visits. What are not clear are the combined effects of these measures in resource-poor settings in rural China, and feasibility of widespread adoption. To obtain such evidence requires a local study done under the unique operational circumstances of rural China and within the resource constraints typical of this setting. The large-scale cluster-randomized controlled proposed here will precisely and reliably define the effect of two highly plausible intervention strategies on important clinical outcomes. The evidence provided by the project will form the basis for policy setting that has the potential to greatly reduce the occurrence of vascular disease in rural China and take an important step towards balancing the rural urban divide in health and healthcare.

# STUDY DESIGN

This project will be a large-scale, factorial, cluster-randomized, controlled trial conducted in rural China with the township as the unit of investigation. The two main interventions to be evaluated are: 1) a primary-care based high cardiovascular risk management package delivered by village doctors; 2) a community-based salt reduction program delivered mainly by community health educators.

## PROVINCES, TOWNSHIPS AND VILLAGES TO BE INCLUDED

The study will be done in 5 Northern Provinces utilizing established collaborations with local academic institutions and governments. The five provinces to be included are Hubei, Liaoning, Ningxia, Shanxi, and Shaanxi. The number of counties in the Provinces ranges from 8 (Ningxia) to 136 (Hebei) and the average number of townships in each county ranges from 12 to 22. These provinces are known to have high rates of vascular disease with a substantial burden caused by high blood pressure. [10]

Two counties will be selected from each Province on the basis of their willingness to participate in the project, their proximity to the local research team and their being broadly representative of the socioeconomic development level of the Province. In each county, 12 townships will be invited to participate in the study with a projected total of 120 townships. Selection of townships will be determined by a number of factors such as convenient transportation and preferences of the country bureau of health. It is important to note that the non-random selection of the counties and townships is not a significant issue for a research project of this design. The key is that assignment of townships to intervention and control conditions is by chance alone and this will be achieved by the randomization process.

All village primary care providers in the same township are managed administratively and supported technically (especially in public health and health education) by the township healthcare center where they regularly meet and receive training and supplies. A design that sought to deliver intervention and control to villages within the same township would be at high risk of contamination because of the township-based organizational structure for healthcare delivery. Therefore, township is chosen as the cluster.

Although the townships are the units for randomization, only 1 village from each township will be selected to participate in the study. In this way, the possible contaminations will be minimized best. The village to be selected should be as central as possible to the geographic area covered by the township, but should not be the Central Village where the township government and healthcare center locate, and the total population should be between 1000 t0 2500. The reasons are to reduce the variance between villages, increase the balance between the intervention and control groups, and decrease the complexity and difficulties that we may have in implementing the intervention and evaluation during the study.

In the selection process, townships can not determine whether they will be in the intervention or control group, which will be governed by the randomization process only. However, if the primary care provider in the selected village declines to participate in the study, the next most geographically central village will be offered participation until a consenting village is found.

## RANDOMIZATION

A stratified block randomization will be used to assign the townships into 4 groups: high CVD risk management package only, salt reduction only, both package and salt reduction, and usual care (neither). Randomization will be stratified by county. In each county, the 12 townships will be divided into 3 blocks according to geographic proximity. In each block, the 4 townships will be randomly assigned to receive both interventions, either intervention, or neither. For the entire study, there will be 60 townships with ‘package’ and 60 townships with ‘no package’ (with or without salt reduction) and 60 townships with ‘salt reduction’ and 60 townships with ‘no salt reduction’ (with or without “package intervention”). There will be 30 townships receiving both the package and the salt reduction interventions, 30 receiving package only, 30 salt reduction only, and 30 receiving neither interventions. All groups will receive the usual care.

## INTERVENTION AND CONTROL

INTERVENTION

For the package intervention, the period of intervention will be 2 years; and for the salt reduction, approximately 1.5 years due to the staged onset of the two interventions.

### ‘Package’ intervention

The intervention package will be based upon the application of the core strategies described in the national practical guidelines for hypertension and cardiovascular disease management at grass-root levels in China, with further adaptation to ensure the interventions are feasible and affordable in rural China. Broadly the package will encourage the opportunistic identification of a set of high-risk individuals and then seek to improve their clinical management through the application of simplified guidelines and a defined follow-up schedule. The package will focus attention on patients considered at high cardiovascular risk on the basis of their falling into one of the following three groups, regardless of current medication use:

- Physician-diagnosed history of coronary heart disease, ischemic stroke, or hemorrhagic stroke, or
- Older age (50 years or older for men; 60 years or older for women – “50+/60+” hereafter) and having physician-diagnosed Type I or type II diabetes
- Older age (50+/60+) and SBP ≥160 mmHg (note that for simplicity, DBP is not included in the criteria)

Details of the interventions are in Appendix 1. Briefly,

- Implementing the CVD risk management package: Village doctors will screen, classify, treat, and follow-up high-risk cardiovascular patients according to the Package. A flow chart summarizing the main points and procedures will be designed as a wall poster and distributed to all doctors in the intervention group as an aid to help them adhere to the Package (see Appendix 1-A).
- Training on package: The training of the Package will be provided to all the participating village doctors in the intervention group. A cardiologist from each county hospital (10 in total) will be trained centrally in Beijing in both the package itself as well as how to be a trainer. The county hospital cardiologists will then train all village doctors in the intervention villages in their county. This “train the trainers” model is being used since it best emulates the practicalities of how the program might be rolled out if it would be proved effective. All village doctors can only begin intervention after they pass the training examination.
- The village doctors will also be trained and asked to complete a simple case management record (see Appendix 1-B for a sample record) documenting the initial clinical visit and each subsequent follow-up visit for all high-risk patients identified.
- Digitized central database and performance feedback: We will establish a central database and management system in Peking University Clinical Research Institute. An independent monitor will be appointed in each county to perform study quality control and to digitize all case management records every 2 months and upload the data via a secure network to the central database. Village doctors will be provided with regular semi-yearly performance feedback on key performance indicators (see Appendix 1-C) generated by the central database to reinforce their compliance to the package.
- Economic incentive: Observed performance will also determine the additional performance-based provider payments made to the village doctors with specific sums allocated for the achievement of specific clinical goals, especially preventive care that they are not normally compensated for such as blood pressure measurement, regular follow-up, lifestyle recommendations, and hypertension control (the last column of Appendix 1-C includes details on how the village doctors will be paid). The frequency of these payments will be the same as performance feedback – once in half a year.

### ‘Package’ control

Villages in the control group will continue their usual practices without the introduction of the ‘package’.

### ‘Salt reduction’ intervention

The salt reduction intervention will be a multi-faceted program including general community health education, specific targeting of patients at high risk of blood pressure-related disease (those targeted by the package) and a food supply measures designed to promote the sale of salt substitute through the village and township convenience stores.

The community-wide education component will seek to tap into the mechanisms already established in the villages for the dissemination of health information, such as township and village radio and TV broadcast system, public announcement system, bulletin boards in the village offices or village clinics, and promotional pamphlets. This will require the active engagement of the village council and the primary health care provider who will be informed and trained by county level public health experts. These county level staff will in turn be trained by central study staff under the same ‘train the trainer’ model used for the implementation for the ‘package’. The community-wide education will include verbal counselling in small groups, public meetings, visual aids and other mechanisms throughout the two-year intervention period. Specific advice will be given to reduce the quantities of salt added to food during cooking, to limit the amount of salt pickled foods eaten, and to soak salt cured meats in water overnight prior to eating them.

The component targeting the high-risk individuals will be delivered primarily by the village health care providers as part of an expanded chronic disease prevention role. Healthcare providers will be trained to opportunistically screen adult patients presenting for any reason by asking simple questions about disease history and making blood pressure measurements where possible. If a patient is identified as possibly falling into the high risk group they will be given particular encouragement to reduce salt intake and informed about the merits of replacing their salt supply with salt substitute. Community health educators may work alongside village doctors to hold special group counselling sessions for high-risk patients and to conduct home visits to demonstrate how to cook low-salt and tasty dishes.

Finally, there will be a concerted effort to make salt substitute available for sale in all village and township stores in the intervention areas. This will be a low-sodium, high-potassium salt-substitute (consisting of 65% sodium chloride, 25% potassium chloride and 10% magnesium sulphate) and the villagers will be encouraged to use the salt substitute to replace all household salt use (cooking, pickling and discretionary mealtime use). Contacts in the relevant Provincial salt manufacturing corporations will be used to ensure a supply of salt substitute and if possible the salt substitute will be subsidized such that it is the same price as or lower price than usual salt.

### ‘Salt reduction’ control

Villages in the control group will continue their usual practices without the introduction of any salt reduction initiatives.

## OUTCOMES

The primary outcomes will be the same for the package intervention and the salt reduction intervention, which is the mean blood pressure changes between the groups..

The primary outcome for the package intervention will be mean blood pressure level of high-risk patients.

The primary outcome for the salt reduction intervention will be mean blood pressure level of older adults (50+/60+).

### Secondary outcomes for the package intervention

- The proportion of high-risk individuals in the village that are identified and regularly managed by the village doctors. Regular management is defined as visiting the village doctor at least once a month, at least 9 times in a year, and measuring blood pressure and prescribing cardiovascular medicine during each visit
- The proportion of high-risk individuals who had visited the village clinic and received pharmaceutical treatment from the village doctor at least once in the past year
- The proportion of high-risk individuals that are treated with a blood pressure lowering agent
- The proportion of high-risk individuals that are treated with aspirin
- The proportion of high-risk individuals that have systolic blood pressure controlled to less than 140mmHg
- The proportion of high-risk individuals that report having received therapeutic lifestyle changes recommendations from the village doctors
- The mean blood pressure level of older adults (50+/60+) in the village
- The proportion of older adults with hypertension (systolic blood pressure >=140 mm Hg)

### Secondary outcomes for the salt reduction intervention

- The proportion reporting efforts to reduce salt consumption
- The proportion with hypertension
- Mean 24-hour urinary sodium excretion (based on a sub-sample)
- Average daily per capita salt sale volume in the village estimated from salt sale records in the village convenience stores

### Exploratory outcomes

A series of exploratory outcomes will be assessed including fatal and non-fatal cardiovascular events and all-cause mortality.

## OUTCOMES EVALUATION

The methods used to collect the data for outcome assessments will be basically the same for both interventions except urinary sodium excretion (salt reduction only) and case management records (package only). All outcome assessments will be done in exactly the same way in every village, regardless of its assignment to intervention or control.

### Baseline and follow-up surveys

A baseline and a follow-up random sample survey (2 independent samples) of older adults (50+/60+) will be conducted in all of the 120 villages before and after the interventions. In each village, the survey will collect data from 40 consenting adults, half men and half women. Sampling will be done by the China International Center for Chronic Disease Prevention located in Beijing acoording to village rosters collected before the survey. All age-eligible (50+/60+) villagers will receive a random number. The random numbers will be ranked according to ascending order and informed consents will be sought according to the rank orders until 20 men and 20 women in each village consent to participate in the survey. Efforts to make sure that only one older adult from the same household will be recruited in the survey will be made – through a roster including household information or someone (e.g. village chief) who knows the villagers well enough to identify adults from the same household. Blood pressure, weight, height and heart rate will be measured and recorded according to standardized protocol. A brief questionnaire (see Appendix 2) will be administered by survey staff to collect information on disease history, medication use, care seeking patterns, and lifestyle factors. Survey participants will receive a small gift valued at approximately 5 yuan (RMB) as a token of appreciation for their participation in the survey. Five yuan is approximately half of the minimal wage per hour for hourly workers.

High-risk patients in the evaluation surveys will be defined slightly differently from the high-risk definition in the package. It will be defined as having history of heart disease or stroke; or older age (50+/60+) and having diabetes; or older age (50+/60+) and having a measured systolic blood pressure > 160 mmHg; or older age (50+/60+) and currently on anti-hypertension medication but having ever had a self-reported BP measurement > 160 mmHg. See Appendix 2 for details on identification of high-risk patients from the random sample of older adults.

Because this is a cluster-randomized trial, it is not hard to outcome assessors to know the randomized allocation of the villages. However, standardized quality control procedures (such as independent reviews of the survey questionnaires and 10% audits) will be followed to ensure that survey personnel conduct the survey in an objective manner. Measurement of blood pressure will be done by an electronic device with recording functions to minimize the possibility of any tampering of the readings by study personnel. A systematic plan for quality control and assurance including the surveys and other components will be formulated and included in a separate document on the standard operation procedures for the study (a summary of which will be included in the protocol).

### 24-hour urinary sodium

A 20% random sample (8 persons), stratified to have 4 from high-risk individuals and 4 from none high-risk individuals, will be selected from the survey participants to collect 24-hour urine. It will give a subsample of 960 participants, 480 in intervention and 480 in control. All urine in a 24-hour period will be collected for these participants, the volume will be recorded and a sample will shipped to a central lab for assays of sodium, potassium and creatinine according to standardized operating procedures. A freezer with-20ºC will be used to store the urine samples before shipping to the central lab in Beijing.

### Mortality and morbidity surveillance system

All village doctors including those in the control group will be required to record and report all deaths and fatal and non-fatal cardiovascular events. Non-fatal events will be collected using the modified WHO MONICA study protocol for acute coronary heart disease and stroke events. All data on non-fatal, hospitalized, and fatal cardiovascular events from the county emergency and hospitalization records (including township healthcare centers if possible) and all deaths from the Public Security Bureau (and/or County Centers for Disease Control and Prevention) death registrations will be used to confirm diseases and causes of death. With sufficient funding, personnel, and training, the “verbal autopsy” method will also be used to assign causes of death. All events will be reported to the central database in Beijing and be adjudicated by an independent adjudication committee.

### Case management records

These records are an integral part of the cardiovascular disease prevention and control package in the management of high-risk patients in the 60 intervention villages. In addition, these records will also provide a large amount of clinical information for better evaluation of the effects of the intervention and better understanding of the mechanisms and processes through which the package exerts its impacts. Such understanding is crucial for future improvement of the package and its widespread adoption.

### Village doctor survey

In order to assess whether the doctors in the intervention and control groups have comparable characteristics, we will collect basic information such as gender, date of birth, education, income, marital status, and years of clinical practice through a very short survey (see Appendix 3) for all the village doctors in the study. This survey will be conducted before the baseline village older adult survey but after obtaining their informed consent to participate in the study. In the case that they choose not to participate in the study even after group consent has been obtained from village leaders, we will select another village in the same township until we find consenting doctors and will conduct the village doctor survey and baseline older adult survey in the consenting village. The village doctor survey will be conducted only once at baseline; no follow-up survey is necessary.

## PROCESS EVALUATION

Aside from a comprehensive outcome evaluation, a process evaluation is also important.[49] The information on how the program is performed can help the investigators to understand the extent to which the program is implemented as planned and explain the outcomes.[50] Data will be collected by study personnel through surveys at baseline, during, and after intervention of all consenting village doctors on their healthcare knowledge and practice patterns. For the doctors in the “package” intervention program, a more in-depth survey of specific questions related to the program implementation at the end of intervention will also be conducted. For both the package and salt reduction interventions, interviews and focus groups at the end of the intervention will be conducted among key stakeholders involved in the programs including county officials, township health care center leaders, village doctors, community health educators, and villagers.

## ECONOMIC EVALUATION

An economic evaluation will provide an assessment of the cost-effectiveness of each of the intervention strategies. The perspective to be adopted will be that of the health sector and will include:

- Costs to government in establishing and running the program including the costs associated with the provider financial incentives. These data will be extracted from financial statements from the project and partner organizations (e.g. hospitals and village clinics). Adjustment will be made to exclude protocol driven costs associated with conducting research
- Associated costs to individuals and government of medical treatment and medications and the potential cost offsets associated with reductions in illness. Data on use of health care services and medications will be extracted from individual patient follow-up interviews. These resource items will be costed at standard prevailing market rates – recognizing variation in such unit costs across regions. Average costs per individual in each of the intervention groups will be estimated over the period of follow-up.

Cost effectiveness will be assessed initially in terms of cost per unit reduction in blood pressure for high-risk patients (package) or for older-adults (salt). However, additional modeling will be conducted to:

- Account for change in program costs associated with the scaling up of the intervention e.g. economies of scale through rationalization of upfront costs
- Extrapolate these trial based cost effectiveness findings into estimates of cost per life year saved and cost per Disability Adjusted Life Year (DALY) averted. These estimates will be based on evidence from the literature of disease progression and long term treatment effects.

These findings will enable the cost-effectiveness of the interventions tested in this study to be benchmarked against standard criteria such as that of the World Health Organization of a cost per DALY averted of less than three times gross national income.[51] Sensitivity analyses will be conduced to assess uncertainty in study findings associated with variation in study parameters.

## STATISTICAL POWER

The sample size has been estimated to provide good statistical power (alpha=0.05, beta=0.90) to detect plausible effects of each intervention. Common assumptions of both sets of power calculations are: 120 clusters, equal randomization between intervention and control and an intra-cluster correlation coefficient of 0.05. These power calculations also assume that there is no interaction between the effects of the package and the effects of salt reduction.

- For the detection of the effects of the package, it is assumed that 8 high risk individuals (20%) will be identified from the 40 survey participants in each cluster, a total of 960 high-risk patients in 120 clusters. Assuming a standard deviation of systolic blood pressure of 15 mmHg, the power to detect a 2.5 mmHg net difference in systolic blood pressure between the intervention and control group pre-post differences will be > 90%. Assuming about half of the high-risk patients will be managed by the village doctors and no difference for those not managed, a 2.5 mmHg difference means a 5 mmHg difference in those actively managed by village doctors. Previous clinical trials demonstrated that even just single drug treatment can lower systolic blood pressure by at least 10 mmHg on average, our targeted blood pressure lowering strategy in package intervention should produce even larger effect. In addition, our CSSS study showed that a low sodium salt substitute could reduce systolic blood pressure by 5.4 mmHg on average. We have reasons to believe that an effect equal or even larger than 5 mmHg systolic blood pressure difference is highly achievable. On the other hand, to achieve a difference smaller than 5 mmHg in systolic blood pressure among those actively managed is not considered meaningful in terms of policy implications. In other words, a smaller effect size is usually not regarded by policy makers as convincing enough for national policies.
- For the detection of the effects of the salt reduction program, assuming a standard deviation of systolic blood pressure of 15 mmHg, the power to detect a 2.5 mmHg net difference in systolic blood pressure between the intervention and control group pre-post differences will be > 90% for a total of 4,800 older adults in the 120 clusters (40 per cluster).

## ANALYSIS PLAN

The primary analyses will be done by comparing the proportions (F-tests) and means (ANOVA) between the intervention townships and the control townships, separately for each intervention. Not only the point estimates but also estimates of variances will be reported as 95% confidence intervals. We will compare the post-intervention differences in the outcomes between the intervention group and the control group. In addition, we will also evaluate the differences between pre-and-post intervention levels for both the intervention and control group to see if the net differences between the 2 groups are statistically significant.

All analyses will include adjustment for the clustering effect and will be done according to the principle of intention to treat.

In both interventions, the main treatment effect will be assumed to be a fixed effect, while the clusters will be assumed to be random. If there are significant differences in the treatment effect among the clusters, either at a cluster level or an individual level, on any of the baseline characteristics, a multilevel modeling approach will be undertaken.

## DATA MONITORING AND SAFETY

A data and safety monitoring board is not considered necessary since all the interventions in the Package are based on current international guidelines and have been proven to be both effective and safe. The salt substitute and low salt diet have also been shown by our own or other’s previous studies as effective and safe.

A data safety and monitoring plan will be established. The safety of study participants will mainly be assured by having expert consultation available to the village doctors and by monitoring digitized case management records. Village doctors will receive clear instructions to refer patients to hospital-based doctors or specialists, whenever necessary. Both emergency and non-emergency referrals are included in the training. The county cardiologist who will train the village doctors will be available for consultation should such needs arise. The case management records taken by the village doctors in the intervention villages will be digitized and uploaded to a central database on a bi-monthly interval. These records will be regularly reviewed by study cardiologists to detect any abnormal patterns including adverse effects and medically appropriate actions will be taken when necessary.

# PROJECT TIMELINE

The two interventions will be implemented at two different stages with the package intervention starting first according to the following schedule and the salt reduction about 6 months later due to practical concerns of the complexity of starting two interventions at the same time.

- May 2010 - July 2010: ethics Committee submissions and approvals
- May 2010 – August 2010: development of standard operating procedures
- September 2010 –October 2010: baseline surveys and training the trainers
- October 2010 – October 2012: intervention
- November 2012 to Jan 2013: follow-up surveys
- February 2013: study close-out and final evaluation survey
- March 2013 – May 2014: data cleaning, analysis, and report writing

# ETHICS REVIEW AND HUMAN SUBJECT PROTECTION

The project will be reviewed by the Ethics Committee of the Peking University Health Science Center in Beijing, China and the Duke University Health System Institutional Review Board, USA. Ethic review will also be conducted by the National Heart, Lung, and Blood Institute, USA.

### Human subject protection certification and training

All collaborating organizations have received the FWA (Federal-Wide Assurance) from the US Department of Health and Human Services Office of Human Research Protections. All study personnels have received or will receive either on-line or in-person trainings on human subject protections and obtain the CITI certificate before they begin the study.

### Participant informed consent

Participant consent for this project has particular challenges because of the community level nature of the intervention and the requirement to obtain community consent in addition to the usual individual consent. The study team will work with our local collaborators to first introduce the study to the provincial bureau of health. After obtaining their support and consent, we will introduce the study to the county department of health to seek their support and input on the selection of townships in their county to participate in the study. The consent process at each level will be similar: First, an initial group presentation and discussion of the project will be conducted. This will be followed by time for reflection and the opportunity for each stakeholder group to consult with the research team individually as required. A final decision to participate, or not, will be made at a second face-to-face meeting of the parties with the final decision provided to the researchers in writing at the county levels.

Individual consent of participants in the surveys will be done in the usual way. Participant information sheet and consent form for the village doctors are in Appendix 4-A; for survey participants in Appendix 4-B.

There will be an estimated 10,000 high-risk patients to be managed in 2 years in 60 villages. No individually identifiable information in the case management records will be collected or digitized by study personnel. Only the first page of the case management records has individually identifiable information. Such information will be entered by the village doctors and can be viewable by only the village doctors and the study monitor digitizing such paper-based data. All high-risk patients in the package intervention group will be identified by a unique study ID. Patient records transmitted to the Data Management Centre will be identified only by a unique number and date of birth; in other words, no individually identifiable information such as name, address, or personal ID will be included in the digitized database. Therefore, there is no need to obtain informed consents from high-risk patients being managed by the village doctors as part of their enhanced clinical practices.

### Participant risks and benefits

Two types of interventions are proposed: package and salt reduction. No or minimal risks will be imposed by the community education intervention (using means of public announcement system, flyers, and town hall meetings etc.) or by usual care planned for the control group. The package intervention will follow standardized guidelines for the identification and treatment of patients with high cardiovascular risks such as hypertension and cardiovascular diseases. These drugs (such as diuretics and aspirin) have been used in the market for many years and have been proven to be safe in most patients. Adequate trainings will be provided to the village doctors who prescribe these drugs. Outcome assessments will involve a simple set of questions, basic anthropometry, a blood pressure measurement and a 24hr urine collection. No or minimal risks will be imposed by these measurements.

A potential risk to the participant is the inadvertent release of his/her information obtained during the course of the study. This possibility is minimized by having computer-readable data records for statistical analyses identified only by code number and maintaining names, addresses, and other identifying information used in the study (when such information is collected such as in the surveys) in separate databases on secure servers with password protection. Paper records and other identifying information are maintained separately in locked files. and all project personnel (including the data management team) will sign data confidentiality agreement and will be thoroughly indoctrinated in maintaining confidentiality of data. All data collected in this study will be kept strictly confidential with access granted to only authorized personnel. No report or manuscript will contain any information that would allow an individual participant in the study to be identified.

Benefits to individual participants include opportunity to have current cardiovascular risk factors evaluated at no cost, and treatments when needed for the package intervention group, and free education for the community education group. More importantly, results of the proposed study, with small or no risk to individual participants, may have important implications for public health policy and allocation of resources for chronic disease prevention and control in rural China, where low-cost, effective, and sustainable strategies are urgently needed. Thus, potential problems in this research are few, low order, and potential benefits very great.

### Inclusion of women, minorities, and children

There will be equal efforts to enroll men and women; thus by design the survey sample will be drawn to include approximately 50% women. The study will be conducted in 5 regions in Northern China where our 5 local partners are located. One of the regions is in Ningxia Hui People Autonomous Region where a lot of Hui minority people reside. More than 30% of the participants in this region, over 6% of overall sample, will belong to the Hui minority group. There are very few minorities living in the other 4 regions and will result in only small numbers of minorities being recruited into the sample there. The participants in the project will primarily be adults since it is adults that mostly suffer from hypertension and chronic diseases; hence, the topic is not relevant to children and none are to be included. However, the community education intervention aimed at all villagers in the same village may benefit children as well.

### Amendments and additional submissions

Amendments to the protocol, participant information sheet or consent form will be submitted to the ethics committee for approval. Such amendments will only be implemented once ethical approval has been obtained, unless an amendment is being made to eliminate immediate hazards to study participants, which is highly unlikely in this study.

Certain components of this study are not determined at this time or will be informed by the results of the initial phase of the study; thus are not available in this protocol. These include details on

- The salt reduction intervention
- Process evaluation
- Economic evaluation
- Morbidity and mortality surveillance
- Procedures to conduct quality audit of the case management records
- Other measures (if undertaken) to ensure study quality

Additional new submissions for these components will be made at a later date when they are ready.

# REFERENCES

# APPENDICES

Appendix 1: Detailed Description of the CVD Prevention and Management Package

Appendix 1-A: Flow Chart to Identify, Manage, and Follow-up High-Risk Patients

APPENDIX 1-B – Case Management Record

Appendix 1-C: Key Performance Indicators and Payment Schemes

APPENDIX 2 – Questionnaire for baseline and follow-up population surveys

APPENDIX 3 – Questionnaire for village doctors

APPENDIX 4-A – Participant Information Sheet and Consent Form to be used for village doctors

APPENDIX 4-B – Participant Information Sheet and Consent Form to be used at time of baseline and follow-up population surveys and interviews

## APPENDIX 1 – Detailed Description of the CVD Prevention and Management ‘Package’

The intervention package will be based upon the application of the core strategies described in the national clinical guidelines for hypertension and cardiovascular disease management in China, with adaptation to ensure the interventions are practical and affordable in rural China. Broadly the package will encourage the identification of a set of high-risk individuals and then seek to improve their clinical management through the application of simplified guidelines and a defined follow-up schedule. This will be achieved by training the primary care providers in the selected villages and supplying an automated electronic sphygmomanometer to each village clinic. The package also includes built-in performance evaluation and feedback mechanisms and a simple performance-based provider payment scheme.

The initial training of the village doctors is considered the beginning of the intervention. Identification and management of high-risk patients will be on a continual basis and last for 2 years (and hopefully will continue even after the end of the study). Therefore, the duration of intervention is 2 years.

*Identification of individuals at high cardiovascular risk* - The package will focus attention on patients considered at high cardiovascular risk on the basis of their falling into one of the following three groups, regardless of current medication use:

- Physician-diagnosed history of coronary heart disease, ischemic stroke, or hemorrhagic stroke, or
- Older age (50 years or older for men; 60 years or older for women – “50+/60+” hereafter) and having physician-diagnosed Type I or type II diabetes
- Older age (50+/60+) and SBP ≥160 mmHg

These individuals will be identified by training the primary care provider to take a brief medical history and measure blood pressure in all patients 50+/60+. This will be done opportunistically when patients visit the village healthcare clinics for any reason. In addition, the primary care providers will be encouraged to seek out all patients known to have previously suffered from cardiovascular disease using their knowledge of the community and the connections that they have within it. The more time-consuming and costly approach of screening all adults will not be done because it would not be sustainable.

Patients that fall into other groups defined for management under existing guidelines (such as patients with SBP < 160 but ≥140 mmHg or DBP < 100 but ≥90 mmHg) will be notified of their status and receive treatment but their management will not be specifically emphasized within this project. The rationale for this approach is that the interventions tested should be feasible for widespread rollout if they are proved effective and this would not be the case if every patient with an SBP of >140mmHg was to be offered long-term drug treatment.

*Management of individuals at high cardiovascular risk -* The intervention component of the program will train the primary care providers to:

- Give all high-risk patients lifestyle recommendations focusing on the consumption of a low-salt diet and smoking cessation;
- Measure blood pressure for every high-risk patient in every visit
- Prescribe evidence-based medications to high-risk patients, focusing on aspirin, diuretics, beta blockers, calcium antagonists, and ACEI/ARB, as appropriate;
- Implement a follow-up program amongst these patients with the goal of ensuring long-term adherence to the recommended interventions;
- Refer difficult to manage patients to the services provided by the township medical center.

The decision making process for patient management, follow-up and referral will be a central aspect of the primary care provider training and will be reinforced by various printed materials. The details of the proposed management algorithms are provided in Appendix 1-A. These flow charts will be re-designed and printed as large colored posters for village doctors to post on the wall of their clinics for easy reference. Explanations regarding the flow charts: the charts appear to be complex with some redundancy because they are designed 1) to separate the algorithm for screening and identification of high-risk patients from the management of different types of patients; 2) to clearly distinguish high-risk patients who will be recorded in the case management records from those who won’t; 3) to emphasize the fact that these patients need to be regularly followed up (like a management loop) instead of only a one-time visit; and 4) to be used as an easy-to-follow almost fool-proof process chart for clinical decision-making. Although the charts look complex, the hope is that once trained, the village doctors will be able to refer to the charts (and the technical manual) for most of their CVD-related clinical needs.

As part of the package, the primary care providers will be trained in the use of a patient-centered case management record that will be used to record relevant clinical indicators of risk, interventions and follow-up visit data. The village doctors will fill these records out manually and keep them in their clinics for each high-risk patient managed by them. The record will be designed as a 13-page printed booklet including the initial visit page and 12 identical pages for follow-up visits (a new booklet will be used for those who finish all 13 visits). The use of these records will not only improve the standardization and quality of clinical practices at the village level but will also be used to provide the basis for performance report and feedback. In addition, the information collected in these records will be used at the completion of the study to better understand the uptake of the package amongst the villagers. See Appendix 1-B for sample case management record.

The whole intervention package has 3 main integral parts: technical training, performance feedback, and provider payment.

**Technical training**

All the primary care providers in the intervention villages (usually one per village) will be trained to adopt and implement the package. The procedure to conduct the technical training part of the package is as follows: at least one cardiologist from the county hospitals will be trained together centrally in Beijing by the study team for a duration of about 2 days. They will receive training on the package itself as well as how to be a trainer. The cardiologists will be responsible to train the village doctors in the intervention group in their county (at least 5, one per village, could be more if the villages have more than one primary care providers).

These trainers have freedom in how to conduct the training, but participatory type of training methods such as case studies and role plays will be taught and highly encouraged. The suggested format to train the village doctors is to have an initial one-day training followed by a half-day or one-day refresher training a month later with more refresher trainings if necessary. Standardized quizzes will be given at the end of each training session and the village doctors who will not achieve a passing score will receive additional training. The cardiologists will also consent to providing on-site technical consultation to the village doctors they train at least twice a year as well as training new primary care providers in the villages in case of staff turnover. This ‘hands off’ training of the villager doctors (or the “train the trainers” model) in the intervention is proposed to best emulate the practicalities of how the programs might be rolled out were they successful.

**Performance feedback**

One full-time study monitor who is familiar with the local culture and dialects will be recruited locally from each county. Study monitors will visit the villages once in 2 months to use a laptop to enter all case management record data into a secure web-based electronic data base centrally managed by Peking University Clinical Research Institute in Beijing. The central database will have the capacity to automatically generate key performance indicators for each primary care provider. These indicators will be based on the final content of the technical training. See Appendix 1-C for a list of the 8 key performance indicators.

A short performance report will include the indicator levels for the village doctors as well as the average level and the level of the top 10% (“best performers”) as benchmarks. Every 6 months (at approximately 3, 9, 15, 21 months after the initial training), the performance report will be given to the village doctors by study monitors or township physicians/healthcare workers. At other times, any major issues detected from the performance indicators will be communicated to the village doctors on a timely basis by the county cardiologists (their trainers) or township physicians. [We need to have SOP to know what is considered a major issue and how to communicate with the village doctors] This mechanism of periodic and irregular timely performance feedback is an essential component of the intervention package and will be used to reinforce the technical training the village doctors have received and will be tied to the provider payment component.

**Provider payment**

The provider payments will reward the village doctors with specific sums of money for the achievement of specific clinical goals. A sliding scale will be designed to determine how much to pay for various levels of performance. Appendix 1-C includes details on the payment scheme (the last column of the table). The doctors will be paid twice a year at the time when their performance reports will be given. If possible, we will ask the township healthcare physicians to deliver the performance report and the provider payment (in cash or through designated accounts at the Agricultural Bank of China.

To the extent possible, we will approach the county officials to see if they agree to use the fund provided by the central government for management of chronic disease by township and village providers to pay the provider payment. These funds are made available recently as part of the healthcare reform in China. They will need to agree to pay the villages in the intervention group according to the algorithms of the study but the other villages in the same township and elsewhere the usual way. One benefit to the counties for providing such payment out of the governmental fund is to have a performance and evidence-based way to pay the providers. If the county do not have such fund or do not agree to provide such payment, the providers will be paid by the study. All village doctors in the intervention villages will receive payment according to their performance either from the county fund or from the study.

## Appendix 1-A: Flow Chart to Identify, Manage, and Follow-up High-Risk Patients


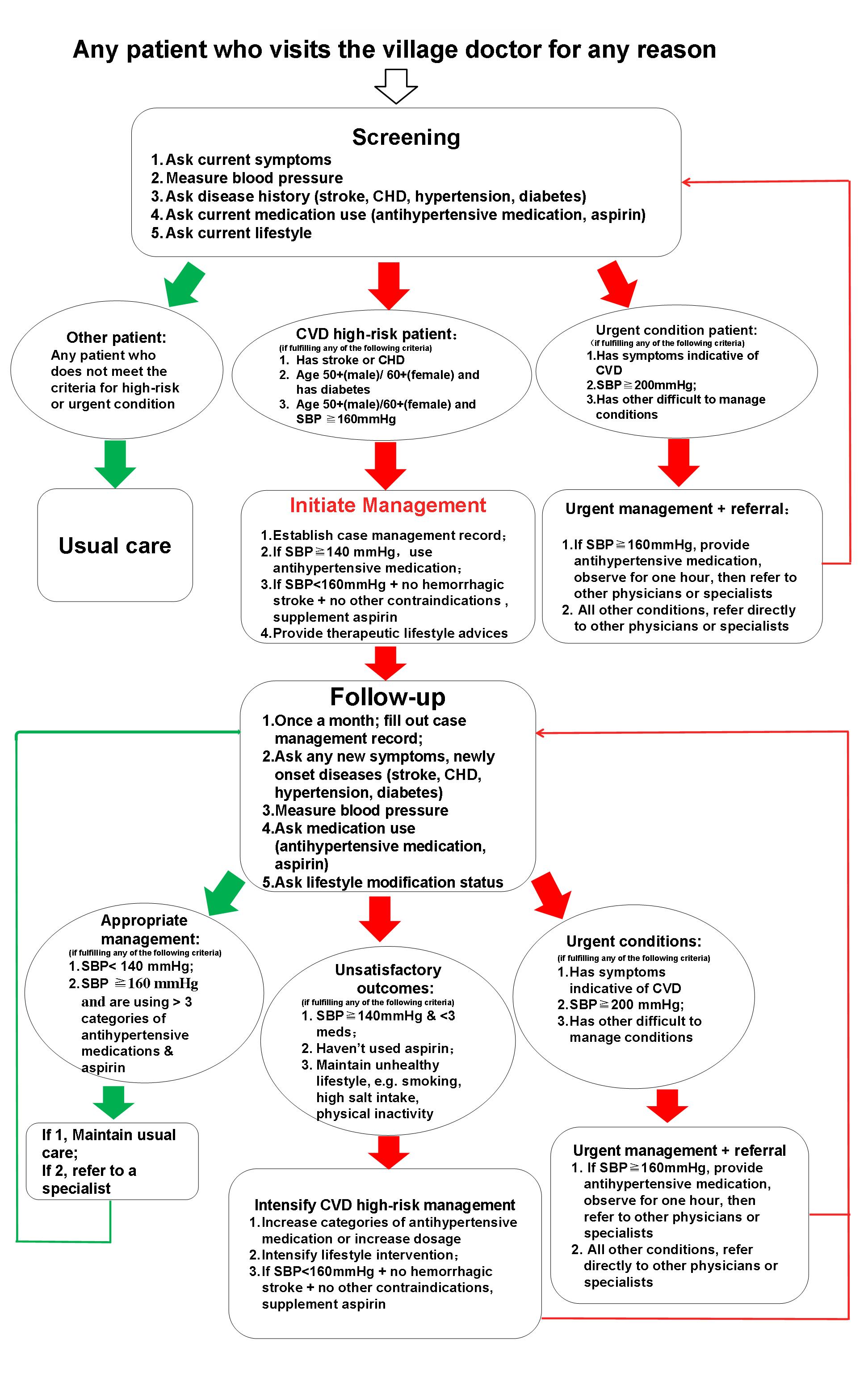


**APPENDIX 1-B – Case Management Record**

**Case Management Record of**

**High-Risk Patients for Cardiovascular Diseases**

Pt. Name

Gender

Date of Birth /

(yyyy) (mm)

Address

Telephone No.

ID No.

Code

Instructions:

1. This record is to be used in the research project of China Rural Health Initiative (CRHI) ONLY. The copyright is possessed by the George Institute, China.
2. This record is to be filled out by village doctors who have joined the research project and been randomized into the intervention group. Please make sure the information provided is true.
3. The following three groups of individuals are defined as high-risk patients for cardiovascular diseases:

**1) The individuals who have past medical history of any one of the following diseases which was diagnosed in county or higher-level hospitals: coronary atherosclerotic diseases (with EKG performed at least once prior to the diagnosis), ischemic strokes, or hemorrhagic strokes;**

**2) Men ≥50 years old, or women ≥60 years old, who have past medical history of diabetes mellitus that was diagnosed in county or higher-level hospitals;**

**3) Men ≥50 years old, or women ≥60 years old, who have systolic blood pressures ≥160mmHg on at least 2 occasions.**

This record is required to be filled out for all of the above high-risk patients. It is not required for other patients.

1. It is required to fill out the follow-up record every month for all the high-risk patients for cardiovascular diseases. If they come to the office more than once in a month, extra records need to be filled out ONLY when changes in treatments are made.
2. Please make sure to record it in “Chief Complaints,” when high-risk patients for cardiovascular diseases visit the office with emergencies.
3. Please make sure to answer all questions with “**□**” by marking “**√**” in appropriate “**□**”. Otherwise, the record is considered incomplete and will not quality for payment.
4. Please make sure to answer all the questions with “ ” by filling out with appropriate information. Otherwise, the CMR is considered incomplete and will not quality for payment.

Thanks for your cooperation!

**Initial Visit**

Date: / / (yy/mm/dd)

**Chief Complaints:**

| **Medical History** |
| --- |
| 1. Does pt have any one of the following diseases (diagnosed in county / higher-level hospitals)？   **CAD**□Yes□No; **Hemorrhagic stroke**□Yes□No; **DM**□Yes□No; **Ischemic stroke**□Yes□No |
| 1. Medications the patient was taking everyday in the past 2 weeks (please record the **name**, **dosage** taken each time, and **times** taken each day, e.g. “Hydrochlorothiazide 12.5mg qd”; if no medication meets the above criteria, please record “NA”). |
| 1. Other medical histories that need to be recorded: |
| **Life Styles** |
| 1. Does the patient take measures to reduce dietary sodium aggressively in everyday life?   □Yes (e.g. do not eat pickled vegetables at all; no MSG and add minimal amount of salt when cooking dishes; unable to eat when the meal is salty.)  □Not so aggressively (e.g. eat some pickled vegetables; take care to reduce salt added in cooking; eat less when the meal is salty.)  □No (e.g. do not pay attention to the amount of salt in food at all.)   1. Is the patient a current smoker？ □Yes □No   If “Yes”, does the patient think about quitting smoking？ □Yes □No   1. Does the patient exercise regularly？(e.g. farming, walking, climbing, etc.)   □Almost everyday □3-5 time/week □1-2 times/week □almost no activities/week |
| **Physical Examination** |
| 1. Blood Pressure (SBP/DBP): / mmHg (This time)   (Optional: / mmHg (Last time), Date: / / (yy/mm/dd)   1. Height: cm; weight: kg; other signs: |
| **Prescription** |
| 1. Referral □Yes □No |
| 1. Therapeutic lifestyle changes (advise the patient to take actions to):   **Reduce salt intake** □Yes □No; **Quit smoking** □Yes □No; **Avoid binge drinking** □Yes □No  **Be physical active** □Yes □No **Control weight** □Yes □No; |
| 1. Pharmaceutical treatment (please record the **name** of the drug, **dosage** taken each time, and **times** taken each day, e.g. “Hydrochlorothiazide 12.5mg qd”; if no drug is prescribed, please record “NA”):   Doctor’s Signature: |

**Follow-up Visits**

Date: / / (yy/mm/dd)

**Chief Complaints**

| **New Conditions and Medication Use** |
| --- |
| 1. From the last visit to the present, did the patient have any one of the following diseases diagnosed (in county or higher-level hospitals)？   **CAD**□Yes□No; **Hemorrhagic stroke**□Yes□No; **DM**□Yes□No; **Ischemic stroke**□Yes□No |
| 1. From the last visit to the present, has the patient been trying to？   **Reduce salt intake** □Yes □No; **Quit smoking** □Yes □No; **Avoid binge drinking** □Yes □No  **Be physical active** □Yes □No **Control weight** □Yes □No; |
| 1. Medications the patient was taking everyday ≥25 days in the past month (please record the **name**, **dosage** taken each time, and **times** taken each day, e.g. “Hydrochlorothiazide 12.5mg qd”; if no medication meets the above criteria, please record “NA”): |
| **Physical Examination** |
| 1. Blood Pressure (SBP/DBP): / mmHg 2. Weight: kg; other signs: |
| **Prescription** |
| 1. Referral □Yes □No |
| 1. Therapeutic lifestyle changes (advise the patient to take actions to):   **Reduce salt intake** □Yes □No; **Quit smoking** □Yes □No; **Avoid binge drinking** □Yes □No  **Be physical active** □Yes □No **Control weight** □Yes □No; |
| 1. Pharmaceutical treatment (please record the **name** of the drug, **dosage** taken each time, and **times** taken each day, e.g. “Hydrochlorothiazide 12.5mg qd”; if no drug is prescribed, please record “NA”):   Doctor’s Signature: |
| **(Optional: the following questions to be asked every 6 months)**   1. Does the patient take measures to reduce dietary sodium aggressively in everyday life?   □Yes (e.g. do not eat pickled vegetables at all; no MSG and add minimal amount of salt when cooking dishes; unable to eat when the meal is salty.)  □Not so aggressively (e.g. eat some pickled vegetables; take care to reduce salt added in cooking; eat less when the meal is salty.)  □No (e.g. do not pay attention to the amount of salt in food at all.)   1. Is the patient a current smoker？ □Yes □No   If “Yes”, does the patient think about quitting smoking？ □Yes □No   1. Does the patient exercise regularly？(e.g. farming, walking, climbing, etc.)   □Almost everyday □3-5 time/week □1-2 times/week □almost no activities/week |

## Appendix 1-C: Key Performance Indicators and Payment Schemes

All indicators are based on the case management records (one record per clinical visit) that the village doctors will fill out for all high-risk patients that they will identify in the village. Performance will be evaluated on a semi-yearly basis.

| **Indicators** | **Definition** | **Measurement** | **Payment Scheme** |
| --- | --- | --- | --- |
| 1. Total number of patients identified | Cumulative number of patients correctly identified by the village doctor since the beginning | The total number of patients who meet the high-risk definition since the first patient being recruited for management. | 1^st^-49^th^ patient, ¥ 3/patient  50^th^-99^th^ patient, ¥ 4/patient  100^th^- patient, ¥ 5/patient |
| 2. Regular management rate | Proportion of high-risk patients regularly managed by the village doctor in the recent half year | The number of high-risk patient with > 5 monthly follow-up records in the past 6 months, divided by the total number of high-risk patients (indicator no. 1) | For patients with > 4 (not 5) monthly follow-up records ONLY  (i.e., no payment for any patients with < 3 records):  ¥ 1/patient/monthly follow-up |
| **The following payments will be made only when 60% regular management rate (indicator no. 2) is reached.** | | | |
| 3. Hypertension control rate | Proportion of hypertensive patients with systolic blood pressure <140 mm Hg in the past 6 months | Among those regularly managed, the number of hypertensive patients with > 5 monthly SBP <140 mmHg (the higher reading of the two measurements in each visit) in the past 6 mo, divided by the total number of hypertensive patients managed | ¥ 2/patient for those who meet the measurement criteria |
| 4. Life style modification rate | Proportion of high-risk patients who indicated making life-style modifications in the past 6 months | The number of high-risk patients with > 5 monthly indicating a practice of both “salt reduction” and “no smoking” in the past 6 months, divided by the total number of high-risk patients identified | ¥ 1/patient for those who meet the measurement criteria |
| 5. Diuretics compliance rate | Proportion of hypertensive patients who report taking diuretics in the past 6 months | The number of hypertensive patients with > 5 monthly indicating that “diuretics” have been taken in the past 6 months, divided by the total number of hypertensive patients | ¥ 1/patient for those who meet the measurement criteria |
| 6. Aspirin compliance rate | Proportion of high-risk patients (hemorrhagic stroke excluded) who report taking aspirin in the past 6 months | The number of high-risk patients (hemorrhagic stroke excluded) with > 5 monthly indicating that “aspirin” has been taken in the past 6 months, divided by the total number of high-risk patients (hemorrhagic stroke excluded) | ¥ 1/patient for those who meet the measurement criteria |
| **The following 2 indicators will be included in the performance report but no payment will be provided.** | | | |
| 7. β-1 blocker compliance rate | Proportion of coronary artery disease patients who report taking β-1 blockers in the past 6 months | The number of coronary artery disease patients with > 5 monthly indicating that “β-1 blockers” have been taken in the past 6 months, divided by the total number of coronary artery disease patients | No payment for this indicator |
| 8. ACEIs/ARBs compliance rate | Proportion of coronary artery disease patients who report taking ACEIs/ARBs in the past 6 months | The number of coronary artery disease patients with > 5 monthly indicating that “ACEIs/ARBs” have been taken in the past 6 months, divided by the total number of coronary artery disease patients | No payment for this indicator |

## APPENDIX 2 – Questionnaire for baseline and follow-up population surveys

**CRHI Survey Questionnaire（2010）**

Hello! My name is . I am a healthcare researcher from (institution). For the purpose of improving health care in your area, we are conducting a health survey to collect information from middle aged or older people in your village. All information collected from this survey will be kept confidential. You are entirely free to decide whether or not to take part in this research study, and there will not be any negative effect to you if you choose not to take part. You are also free to discontinue your involvement with the research study at any time. Thank you for your support and cooperation!

Date (2010/mm/dd) **2010/□□/□□**

Time (hour: minutes) **□□:□□**

| **A. General information** | | | | | | | | | | | | | |
| --- | --- | --- | --- | --- | --- | --- | --- | --- | --- | --- | --- | --- | --- |
| A.1 Name:  A.2 Sex: □_1._Male □_0._Female  A.3 ID**□□□□□□□□□□□□□□□□□□**  A.4 Years of age  A.5 Years of schooling | | | | | | | | | | | | | |
| **B. Lifestyle information** | | | | | | | | | | | | | |
| B.1 Have you smoked during the past 3 months? (multiple choice)  □_1._Yes, cigarette B.1.1 On average, how many cigarettes per day  □_2_.Yes, tobacco leaf B.1.2 On average, how much “Liang” per day  □_3._Other (go to B.2)  □_4_ No (go to B.2) | | | | | | | | | | | | | |
| B.2 Have you drunk alcohol during the past 3 months? (multiple choice) | | | | | | | | | | | | | |
| □_1_. Chinese white wine | | | □_2_. Beer | | | | | | | □_0_. No  (go to B.3) | | | □_3._Other  (go to B. 3) |
| B.2.1How often?  □_1_. <Once/week  □_2_. 1-2times/week  □_3_. 3-5times/week  □_4_. 1-2times/day  □_5_. >Twice/day | B.2.2 How much per tima?  □_1._<１Liang  □_2._1-3 Liang  □_3._3-5 Liang  □_4._>halt Jin  (note:1Liang=50g≈50ml ;1 Jin=500g≈0.5L) | | B.2.4 How often?  □_1_. <Once/week  □_2._1-2times/week  □_3._3-5times/week  □_4_. 1-2times/day  □_5_. >Twice/day | | | | | B.2.5 How much per time?  □_1._<1bottle  □_2._2-5bottles  □_3._6-10bottles  □_4._>10bottles  (1 bottles=750ml) | |  | | | |
| B.2.3 How many times did you drink alcohol more than half Jin? | | | B.2.6 How many times did you drink alcohol more than 10 bottles? | | | | | | |  |  |  |  |
| B.3 Have you done any farming activities during the past 3 months? （30 minutes or above per time）  □_1_. Almost everyday  □_2_. 3-5days/week  □_3_. 1-2days/week  □_4_. Almost none | | | | | | | B.4 Have you exercised during the past 3 months?  （30 minutes or above per time）  (e.g., waling、running、dancing，Taiqi）  □_1_. Almost everyday  □_2_. 3-5days/week  □_3_. 1-2days/week  □_4_. Almost none | | | | | | |
| **Physical examination (PE.1). Blood pressure and heart rate measurement (1st)** | | | | | | | | | | | | | |
| PE.1.1 Blood pressure（Systolic/Diastolic）:  PE.1.1.1**□□□**/ PE.1.1.2**□□□** mmHg | | | | | | | PE1.2 Heart rate:  PE.1.2.1**□□□**/minute | | | | | | |
| B.5 How often do you usually eat pickled foods?  □_1_At least once a day  □_2_3-6 times/week  □_3_1-2 times/week  □_4_Sometimes  □_5_Almost none | | | | | | | B.6 How do you do if the food is tasteless when you dine?  □_1_ Always eat pickle/add soy source/add salt  □_2_ Sometimes eat pickle/add soy source/add salt  □_3_Don’t mind | | | | | | |
| B.7 High salt intake will  □_1_ Bad for health  □_2_ Good for health  □_3_ No effect on health  □_4_ Don’t know | | B.8 Which of the following will reduce blood pressure?  □_1_Medicine  □_2_Smoking  □_3_Est less salt  □_4_Eat more food | | | B.9 How much daily salt intake is recommended for adults?  □_0_ Do not eat salt  □_1_≤ 6 g  □_2_＞ 6 g  □_3_ Don’t know | | | | | | | | |
| **C. Disease history** | | | | | | | | | | | | | |
| C.1 Do you have diabetes?  □_1_. Yes □_0_.No □_9._Don’t know (if No or Don’t know, go to C.2)  D.1.1 Diagnosed by county or higher levels hospitals?  □_1_. Yes □_0_. No □_9_. Don’t know  C.2 Do you have stroke?  □_1_. Yes □_0._No □_9_. Don’t know　(if No or Don’t know, go to C.3)  C.2.1 Diagnosed by county or higher levels hospitals?  □_1_. Yes □_0_. No □_9_. Don’t know  C.2.2 Ischemic stroke or hemorrhagic stroke?  □_1_. Ischemic stroke □_2_. Hemorrhagic stroke □_3_. Don’t know  C.3 Do you have coronary heart disease?  □_1_. Yes □_0_. No □_9_. Don’t know (if No or Don’t know, go to C.4)  C.3.1 Diagnosed by county or higher levels hospitals?  □_1_. Yes □_0_. No □_9_. Don’t know  C.3.2 Had electrocardiograph evidence of CHD?  □_1_. Yes □_0_. No　 □_9_. Don’t know  C.4 Do you have hypertension?  □_1_. Yes □_0_. No □_9_. Don’t know  C.4.1 How many years have you had hypertension? Years □_9_. Don’t know  C.5 Have you had your blood pressure measured after age 50 (male)/60 (female)?  □_1_. Yes □_0._No □_9_. Don’t know (if No or Don’t know, go to C.6)  C.5.1 Please provide the highest systolic blood pressure **□□□**mmHg | | | | | | | | | | | | | |
| C.6 During the past 12 months, did you see your village doctor once a month?  □_1_. Yes  □_0_. No | | | | | | | | | C.7 Does your village doctor measure your blood pressure during visits?  □_1_. Never see village doctor  □_2_. Never measure  □_3_. Sometimes(occasionally)  □_4_. Almost every time  □_5_. Every time | | | | |
| **Physical examination (PE.2). Blood pressure and heart rate measurement (2nd)** | | | | | | | | | | | | | |
| PE.2.1 Blood pressure（Systolic/Diastolic）  PE.2.1.1**□□□**/ F.1.2**□□□** mmHg | | | | | | PE.2.2 Heart rate:  PE.2.2.1**□□□**/minute | | | | | | | |
| **Physical examination (PE.3). Body height and weight measurement** | | | | | | | | | | | | | |
| PE.3.1 Height **□□□.□** cm | | | | | | PE.3.2 Weight **□□□.□**kg | | | | | | | |
| | **CL．CVD high-risk patient confirmation checklist (CL) by the interviewer** | | | --- | --- | | CL.1 Have confirmed diabetes（C.1.1 answer Yes） | □_1._Yes □_0._No | | CL.2 Have confirmed stroke（C.2.1 answer Yes） | □_1._Yes □_0._No | | CL.3 Have confirmed CHD（Both C.3.1 and C.3.2 answer Yes） | □_1._Yes □_0._No | | CL.4 Have reported SBP at older age ≧ 160mmHg （C.5.1） | □_1._Yes □_0._No | | CL.5 Twice systolic measurements ≧160mmHg （PE.1.1 and PE.2.1） | □_1._Yes □_0._No | | **If any of the above is ticked YES, go to D section; otherwise, end survey** | | | | | | | | | | | | | | | |
| **D. Medication use and medical care (high-risk patients ONLY)** | | | | | | | | | | | | | |
| D.1 When was the last time that you saw your village doctor for your heart disease, stroke, diabetes or hypertension problem(s)? days ago  During that visit, did village doctor provide the following recommendations?  D.1.1 Reduce salt intake □_1._Yes □_0._No  D.1.2 Quit smoking □_1._Yes □_0._No  D.1.3 Avoid binge drinking □_1._Yes □_0._No  D.1.4 Be physically active □_1._Yes □_0._No  D.1.5 Control weight □_1._Yes □_0._No | | | | | | | | | | | | | |
| D.2 During the past 12 months, due to your heart disease, stroke, diabetes or hypertension problem(s)  D.2.1 How many times have you been in hospital?  （if no，go to D.3）  D.2.2 How many days have you been in hospital?  D.2.3 How much have you spent for hospitalization? RMB | | | | | | | | | | | | | |
| D.3 How many months have you taken anti-hypertensive medication (AHM) during the past 12 months?  □_1._≥9 months  □_2._2-8 months  □_3._＜2 months  □_4_ Not a long-term user  □_5_. Don’t know if have taken AHM  （if not a long-term user or don’t know, go to D.4） | | | | D.3.1Who prescribed the AHM?  □_1_. Buy by self  □_2_. TCM doctor  □_3_. Specialist  □_4_. Village doctor and specialist  □_5_. Village doctor | | | | | | | | D.3.3 Where do you but AHM?  □_1_. Village clinic  □_2_. Township center  □_3_. County hospitals  □_4_. Pharmacies | |
|  |  |  |  | D.3.2. Has village doctor adjusted the treatments, either dosage or number of AHM for you?  □_1._Yes □_0._No | | | | | | | | | |
| D.4 How many months have you taken aspirin during the past 12 months?  □_1._≥9 months  □_2._2-8 months  □_3._＜2 months  □_4_ Not a long-term user  □_5_. Don’t know if have taken AHM  （if not a long-term user or don’t know, go to D.5） | | | | D.4.1 Who prescribed the aspirin?  □_1_. Buy by self  □_2_. TCM doctor  □_3_. Specialist  □_4_. Village doctor and specialist  □_5_. Village doctor | | | | | | | D.4.2 Where do you but aspirin?  □_1_. Village clinic  □_2_. Township center  □_3_. County hospitals  □_4_. Pharmacies | | |
| D.5 Have you taken any medication for your heart disease, stroke, diabetes or hypertension problems(s) during the past 1 month?   | □_1._Yes □_9._Don’t know | □_0._No (if No, end survey) | | --- | --- |  | D.5.1 Medicine (brand name) | D.5.2 Use in all days?  (>25days in the last 30 days) | D.5.3 Prescribed by village doctor? | | --- | --- | --- | | 1. | □_1._Yes □_0._No | □_1._Yes □_0._No | | 2. | □_1._Yes □_0._No | □_1._Yes □_0._No | | 3. | □_1._Yes □_0._No | □_1._Yes □_0._No | | 4. | □_1._Yes □_0._No | □_1._Yes □_0._No | | 5. | □_1._Yes □_0._No | □_1._Yes □_0._No | | **（End of survey）** | | | | | | | | | | | | | | | | |
| Interviewer impressions: interviewee understands questions  □_1_Well □_2_Acceptable □_3_Does not understand | | | | | | | | | | | | | |
| Time of completion：**□□:□□**  Interviewer signature： | | | | | | | | | | | | | |

Date of quality review: 2010 __ month__ day time: **□□:□□**

Quality reviewer signature: _____________________

## APPENDIX 3 – Questionnaire for village doctors

|  | |
| --- | --- |
| 1. Name: | 2. Sex: □Male □Female |
| 3. Date of birth (yy/mm/dd): □□/□□/□□ | |
| 4. Education:  □Vocational school □2-year college □3-year college □University | |
| 5. Time spending on training program at county (days) and at township (day). | |
| 6. Residential location: □This village □Other village □Other | |
| 7. Martial status: □Married □Other | |
| 8. What percentage of your total workload contributes to be as a village doctor? % | |
| 9. Years of clinical practice as a village doctor Years | |
| 10. What is your total personal monthly income on average?  □<500 RMB  □500-1000 RMB  □1000-2000 RMB  □2000-3000 RMB  □>3000 RMB | |

## APPENDIX 4-A – Participant Information Sheet and Consent Form to be used for village doctors

You are being invited to take part in this research study because the China Rural Health Initiative (CRHI) will be conducting a heart disease and stroke prevention and control study in your township. Please read through this consent form carefully and take your time making your decision. As study interviewer discusses this consent form with you, please ask him/her to explain any words or information that you do not clearly understand. We encourage you to talk with your family or friends before you decide to take part in this research study. You are entirely free to decide whether or not to take part in this research study, and there will not be any negative effect to you if you choose not to take part. You are also free to discontinue your involvement with the research study at any time.

**WHY IS THIS RESEARCH STUDY BEING DONE?**

Heart disease and stroke are the leading causes of death in China. High blood pressure is the leading modifiable risk factor for these diseases whereas high salt intake is a major cause of high blood pressure. In rural and northern regions in China, high salt intake, high blood pressure and stroke are common and serious publc health problems. In order to ease such great disease burdens in rural China, a set of management programs have been designed and will be implemented.

The purpose of this research study is to objectively evaluate the effectiveness of a simplified heart disease and stroke prevention and control package delivered by village doctor and a community-based salt reduction program in rural China.

**HOW MANY PEOPLE WILL TAKE PART IN THIS RESEARCH STUDY?**

A total of 120 townships in 5 provinces (one village in each township) will be involved in the study. All village doctors in these villages (approximately 120 village doctors) will take part in this research study .

**HOW LONG WILL THIS RESEARCH STUDY LAST?**

Approximately 2.5 years.

**WHAT IS INVOLVED IN THE RESEARCH STUDY?**

A total of 120 townships will be randomized into 4 groups, 30 townships in each group. Once the randomization is determined at the township level, your village will be randomly assigned to either one of the following 4 groups: 1) Package intervention group, 2) Salt reduction intervention group, 3) Package plus salt reduction intervention group, and 4) Control group. The randomization procedure will be processed by computerized program.

- If your village has been assigned to the package intervention group and you agree to take part in this research study, you will be asked to sign this consent form and will be required to

1. Complete a short questionnaire about your personal information which includes questions like name, sex, age, education background, and years of engaging in this profession as a village doctor.
2. Be trained to use the simplified heart disease and stroke management guideline to screen, manage and follow-up high-risk patients in your village. You will receive regular performance feedback and related incentives.
   - If your village has been assigned to the salt reduction intervention group and you agree to take part in this research study, you will be asked to sign this consent form and will be required to
3. Complete a short questionnaire about your personal information which includes questions like name, sex, age, education background, and years of engaging in this profession as a village doctor.
4. Support the county health educators to conduct some salt reduction programs in your village. Most of the interventions will be conduced by the health educators.

- If your village has been assigned to the package plus salt reduction intervention group and you agree to take part in this research study, you will be asked to sign this consent form and will be required to

1. Complete a short questionnaire about your personal information which includes questions like name, sex, age, education background, and years of engaging in this profession as a village doctor.
2. Be trained to use the simplified heart disease and stroke management guideline to screen, manage and follow-up high-risk patients in your village. You will receive regular performance feedback and related incentives.
3. Support the county health educators to conduct some salt reduction programs in your village. Most of the interventions will be conduced by the health educators..

- If your village has been assigned to the control group and you agree to take part in this research study, you will be asked to sign this consent form and will be required to

1. Complete a short questionnaire about your personal information which includes questions like name, sex, age, education background, and years of engaging in this profession as a village doctor.
2. Continue your clinical practices as usual.

**WHAT ARE THE RISKS OF THE RESEARCH STUDY?**

- - If your village has been assigned to the package intervention group and you agree to take part in this research study, you will need to spend extra time and energy in addition to your routine to follow the guidelines for screening, management, and follow-up of high-risk patients.
  - If your village has been assigned to the salt reduction intervention group and you agree to take part in this research study, you will need to spend extra time and energy in addition to your routine to support the salt reduction program.
  - If your village has been assigned to the package plus salt reduction intervention group and you agree to take part in this research study, you will need to spend extra time and energy in addition to your routine to follow the guidelines for screening, management, and follow-up of high-risk patients and to to support the salt reduction program.
  - If your village has been assigned to the control group and you agree to take part in this research study, there are no physical and psychological risks associated with this research study.

**ARE THERE BENEFITS TO TAKING PART IN THE RESEARCH STUDY?**

- - If your village has been assigned to the package intervention group and you agree to take part in this research study, you will receive free training programs, performance feedback, and incentives which may help you to improve the quality of your clinical practice on hypertension and cardiovascular disease management.
  - If your village has been assigned to the salt reduction intervention group and you agree to take part in this research study, you will learn some health education skills on salt reduction which may strengthen the impact of your future clinical practice on hypertension prevention and control and community outreach.
  - If your village has been assigned to the package plus salt reduction intervention group and you agree to take part in this research study, you will receive free training programs, performance feedback, and incentives which may help you to improve the quality of your clinical practice on hypertension and cardiovascular disease management; also you will learn some health education skills on salt reduction which may strengthen the impact of your future clinical practice on hypertension prevention and control and community outreach.
  - If your village has been assigned to the control group and you agree to take part in this research study, there are no benefits associated with this research study. However, we hope that in the future the information learned from this study will benefit you, patients in your clinic and farmers in your village, and other people.

**WILL MY INFORMATION BE KEPT CONFIDENTIAL?**

Study records that identify you will be kept strictly confidential as required by law. Peking University Clinical Research Institute will provide safeguards for privacy, security, and authorized access. Except when required by law, you will not be identified by name, address, telephone number, or any other direct personal identifier in study records disclosed outside of Peking University Clinical Research Institute. All data will only be used for the purpose of academic research; and will not be released in any way or leak to the third party

**WHAT ARE THE COSTS?**

Study sponsor will be responsible for all of the cost associated with this research study. There will be no additional costs to you as a result of being in this research study.

**WHAT ABOUT COMPENSATION?**

There is no compensation associated with this research study.

**WHAT ABOUT MY RIGHTS TO DECLINE PARTICIPATION OR WITHDRAW FROM THE RESEARCH STUDY?**

You may choose not to be in the research study, or, if you agree to be in the research study, you may withdraw from the research study at any time. If you withdraw from the research study, no new data about you will be collected for study purposes other than data needed to keep track of your withdrawal. All data that have already been collected for study purposes will be sent to the Peking University Clinical Research Institute.

Your decision not to participate or to withdraw from the research study will not involve any penalty or loss of benefits to which you are entitled, and will not affect your current employment status. If you do decide to withdraw after initially agreeing to participate, we ask that you let interviewer know that you are withdrawing from the study in writing. You will be asked to fill out a simple withdrawal form stating the date and reason for withdrawal.

**WHOM DO I CALL IF I HAVE QUESTIONS OR PROBLEMS?**

For questions about your rights as a research participant, or to discuss problems, concerns or suggestions related to the research, please contact the Beijing study coordinating center Ms Jesse Hao

Telephone: +86 10 8280-0577 EXT 310

Facsimile: +86 10 8280-0177

Email: [jhao@george.org.cn](mailto:jhao@george.org.cn)

Post address: Suit 1302, Tower B, Horizon Tower. No. 6, Zhichun Road, Haidian District, Beijing, 100088, P.R. China.

Or you can contact your local study researchers

Liaoning 024-83282632

Shaanxi 029-82655107，

Hebei 0311-86573288

Ningxia 0951-6980144,

Shanxi 0355-3033238

**STATEMENT OF CONSENT**

"The purpose of this study, procedures to be followed, risks and benefits have been explained to me. I have been allowed to ask questions, and my questions have been answered to my satisfaction. I have been told whom to contact if I have questions, to discuss problems, concerns, or suggestions related to the research, or to obtain information or offer input about the research. I have read this consent form and agree to be in this study, with the understanding that I may withdraw at any time. I have been told that I will be given a signed copy of this consent form."

__________________________________________ ___________

Signature of Subject Date

__________________________________________ ___________

Signature of Person Obtaining Consent Date

## APPENDIX 4-B – Participant Information Sheet and Consent Form to be used at time of baseline and follow-up population surveys and interviews

You are being invited to take part in this research study because the China Rural Health Initiative will be conducting a cardiovascular disease prevention and control package (intervention) delivered by your village doctor. Please read through this consent form carefully and take your time making your decision. As study interviewer discusses this consent form with you, please ask him/her to explain any words or information that you do not clearly understand. We encourage you to talk with your family or friends before you decide to take part in this research study.

**WHY IS THIS STUDY BEING DONE?**

The purpose of this research study is to objectively evaluate the effectiveness of a cardiovascular disease prevention and control program and a salt reduction program.

**HOW MANY PEOPLE WILL TAKE PART IN THIS STUDY?**

Approximately 4800 people will take part in this research study at 120 different villages in 5 provinces.

**WHAT IS INVOLVED IN THE STUDY?**

If you agree to take part in this study, you will be asked to sign this consent form and will be interviewed by researcher and required to answer a series of survey questions about your general information, lifestyle, cardiovascular disease history, medication use and medical care statue at the village clinic. Also, we will measure your body height, weight, blood pressure and heart rates. It will take about 30 minutes to complete all of the procedures.

**WHAT ARE THE RISKS OF THE STUDY?**

There are no physical risks associated with this study.

**ARE THERE BENEFITS TO TAKING PART IN THE STUDY?**

If you agree to take part in this study, there are no direct benefits to you. However, we hope that in the future the information learned from this study will benefit you and other people.

**WILL MY INFORMATION BE KEPT CONFIDENTIAL?**

Study records that identify you will be kept confidential as required by law. Peking University Clinical Research Institute will provide safeguards for privacy, security, and authorized access. Except when required by law, you will not be identified by name, address, telephone number, or any other direct personal identifier in study records disclosed outside of Peking University Clinical Research Institute. All data will only be used for the purpose of academic research, and will not be released in any way or leak to the third party

**WHAT ARE THE COSTS?**

Study sponsors will be responsible for all of the cost associated with this study. There will be no additional costs to you as a result of being in this study.

**WHAT ABOUT COMPENSATION?**

If you agree to take part in this study, you will receive a small gift worth about 5 yuan provided by the study organization.

**WHAT ABOUT MY RIGHTS TO DECLINE PARTICIPATION OR WITHDRAW FROM THE STUDY?**

You may choose not to be in the study, or, if you agree to be in the study, you may withdraw from the study at any time. If you withdraw from the study, no new data about you will be collected for study purposes other than data needed to keep track of your withdrawal. All data that have already been collected for study purposes will be sent to the Peking University Clinical Research Institute.

Your decision not to participate or to withdraw from the study will not involve any penalty or loss of benefits to which you are entitled, and will not affect your access to health care in your community.

**WHOM DO I CALL IF I HAVE QUESTIONS OR PROBLEMS?**

For questions about your rights as a research participant, or to discuss problems, concerns or suggestions related to the research, please contact the Beijing study coordinating center Ms Jesse Hao

Telephone: +86 10 8280-0577 EXT 310

Facsimile: +86 10 8280-0177

Email: [jhao@george.org.cn](mailto:jhao@george.org.cn)

Post address: Suit 1302, Tower B, Horizon Tower. No. 6, Zhichun Road, Haidian District, Beijing, 100088, P.R. China.

Or you can contact your local study researchers

Liaoning 024-83282632

Shaanxi 029-82655107，

Hebei 0311-86573288

Ningxia 0951-6980144,

Shanxi 0355-3033238

**STATEMENT OF CONSENT**

"The purpose of this study, procedures to be followed, risks and benefits have been explained to me. I have been allowed to ask questions, and my questions have been answered to my satisfaction. I have been told whom to contact if I have questions, to discuss problems, concerns, or suggestions related to the research, or to obtain information or offer input about the research. I have read this consent form and agree to be in this study, with the understanding that I may withdraw at any time. I have been told that I will be given a signed copy of this consent form."

__________________________________________ ___________

Signature of Subject Date

__________________________________________ ___________

Signature of Person Obtaining Consent Date
